# Sepsis 2018: Bangkok, Thailand. 1-3 October 2018

**DOI:** 10.1186/s40635-018-0196-z

**Published:** 2018-10-01

## P1 Safety and pharmacodynamic activity of a novel TREM-1 pathway inhibitory peptide in septic shock patients: phase IIa clinical trial results

### Bruno François^1^, Xavier Wittebole^2^, Ricard Ferrer^3^, Jean-Paul Mira^4^, Thierry Dugernier^5^, Sébastien Gibot^6,7^, Marc Derive^8^, Peter Pickkers^9^, Jean-Jacques Garaud^8^, Miguel Sanchez^10^, Margarita Salcedo-Magguilli^8^, Pierre-François Laterre^2^

#### ^1^Medical-Surgical ICU department and Inserm CIC1435, CHU Limoges, France; ^2^Department of Critical Care Medicine, St Luc University Hospital, Université Catholique de Louvain, Brussels, Belgium; ^3^ICU department, Vall d'Hebron University Hospital, Barcelona, Spain; ^4^Medical ICU, Cochin Hotel-Dieu, AP-HP, Paris, France; ^5^ICU department, Clinique St. Pierre, Ottignies, Belgium; ^6^Medical ICU department, Hospital Central, CHU Nancy, France; ^7^Inserm U1116, Nancy Medical Faculty, Lorraine University, France; ^8^Inotrem SA, Paris, France; ^9^ICU department, Radboudumc Hospital, Nijmegen, The Netherlands; ^10^ICU department, Hospital Clínico San Carlos, Madrid, Spain

##### **Correspondence:** Bruno Francois (Bruno.Francois@chu-limoges.fr)

Background

Nangibotide (LR12) peptide is a specific TREM-1 inhibitor. In preclinical septic shock models, nangibotide was able to restore appropriate inflammatory response, vascular function, and improved survival. In phase I, nangibotide was found to be safe and well tolerated up to the highest dose (6 mg/kg/h).

Materials and methods

International, multi-center phase IIa randomized, double-blind, two-stage, placebo-controlled study (NCT03158948). Main inclusion criteria were septic shock according to Sepsis 3 definition and nangibotide to be initiated within 24 hours of shock onset. Patients were randomized to receive either placebo, 0.3, 1 or 3 mg/kg/h of nangibotide. Stage-1 investigated ascending doses. In stage-2 patients were randomized to complete 12 patients in each group. Study drug was infused until end of vasopressors + 12h or up to 5 days. Safety data were reviewed by an independent Data Safety Monitoring Board (DSMB). Primary endpoint was safety and tolerability. Patient follow-up period was 90 days.

Results

50 patients were randomized and 49 treated (1 patient died before dosing). All groups were well balanced in terms of baseline characteristics, except for APACHE II score which tend to be non-significantly lower in placebo group. Primary infection source was 40% abdominal, 50% pulmonary and 10% urinary.

Nangibotide was safe and well tolerated in all groups. During the trial, the DSMB did not raise any safety concern. The number of SAEs/AEs and the number of patients with SAEs/AEs was comparable between all groups (Table 1). Most frequent AEs were atrial fibrillation, anemia, pleural effusion and thrombocytopenia.

Ventilator and vasopressors free days alive were similar in all groups (Table 2). All-cause mortality at day-28 was 14% (5/37) in pooled nangibotide groups and 25% (3/12) in placebo group. In the subgroup with sTREM-1 levels above median, the day-5 mortality was calculated as 40% (2/5) and 20% (4/20) in placebo and nangibotide groups respectively. A trend toward a decrease in circulating levels of endothelium injury markers was observed in nangibotide-treated patients.

Conclusion

Nangibotide was shown to be safe and well tolerated in septic shock patients. Although this small exploratory study was not powered to conclude on efficacy, a signal with non significant lower mortality was observed in the nangibotide group. These results support the need of a larger study to demonstrate the role of nangibotide in the treatment of septic shock.

**Table 1 (abstract P1).** Treatment emergent adverse event and serious adverse event

Patients with at least one	PlaceboN=12	0.3N=13	1.0N=12	3.0N=12	TotalN=49
TEAE	10 (83.3%)	12 (92.3%)	12 (100.0%)	11 (91.7%)	45 (91.8%)
Severe TEAE	8 (66.7%)	6 (46.2%)	5 (41.7%)	4 (33.3%)	23 (46.9%)
TEAE related to the study drug	2 (16.7%)	-	2 (16.7%)	-	4 (8.2%)
Serious AE incl. death	7 (58.3%)	4 (30.8%)	2 (16.7%)	4 (33.3%)	17 (34.7%)

**Table 2 (abstract P1).** Vasopressors and ventilator free days

Days alive and free of	PlaceboN=12	0.3N=13	1.0N=12	3.0N=12	TotalN=49
Vasopressors median (min/max)	25.5 (0/35)	25.0 (0/33)	25.5 (0/31)	23.0 (0/28)	24.0 (0/35)
IMV median (min/max)	24.0 (0/36)	22.0 (0/35)	25.5 (0/30)	24.0 (0/36)	23.0 (0/36)

## P2 Difference of lactate level for predict mortality between cirrhosis and non-cirrhosis septic shock patient

### Surat Tongyoo, Thavinee Trainarongsakul, Tanuwong Viarasilpa, Chairat Permpikul

#### Department of Medicine, Faculty of Medicine, Siriraj Hospital, Mahidol University, Bangkok, Thailand

##### **Correspondence:** Surat Tongyoo (surat.ton@mahidol.ac.th)

Background

Serum lactate is an important marker of tissue perfusion. Impaired hepatic function especially among cirrhosis patients could decrease lactate elimination and result in higher lactate level during septic shock. Whether serum lactate level could be used to predict outcome of cirrhosis patients with septic shock had not been identified. This study was thus aimed to evaluate the utility of serum lactate to predict mortality, comparing between cirrhosis and non-cirrhosis septic shock patients.

Materials and methods

This is a retrospective study, included adult septic shock who were admitted at Siriraj hospital between April 2011 and December 2017. Patients’ baseline information, severity score and lactate level were recorded. The diagnosis of cirrhosis was documented if there was an evidence of small sized liver with diffuse surface nodularity detected by ultrasound, computed tomography or magnetic resonance imaging. The primary outcome was hospital mortality.

Results

From 777 enrolled septic shock patients, 91 were previously diagnosed cirrhosis. The patients’ age, gender, baseline arterial pressure and severity score were not different between cirrhosis and non-cirrhosis patients. Cirrhosis patients had significant higher body mass index, lower temperature and slower heart rate. Baseline serum lactate and at shock reversal were significantly higher in cirrhosis patients (baseline, 7.5+6.1 vs 4.3+3.7 mmol/L; P<0.001 and at shock reversal 4.5+4.1 vs 3.2+3.0 mmol/L; P=0.01). Lactate clearance was not different (27.3+24.7 vs 23.5+21.5 percent; P=0.35) and so was hospital mortality (36.3% in cirrhosis vs 30.1% in non-cirrhosis; P=0.23). The receiver operating characteristic (ROC) curve analysis identifies that baseline lactate is the most reliable prognostic factor for hospital mortality according to the area under the curve (AUC). (Table 1) The cut-off point to predict hospital mortality was 5 mmol/L for cirrhosis and 4 mmol/L for non-cirrhosis patients.

Conclusions

Baseline serum lactate in cirrhosis septic shock patients was higher than non-cirrhosis patients. The cut-off point of baseline lactate to predict hospital mortality was 5 mmol/l and 4 mmol/l among cirrhosis and non-cirrhosis septic shock patients.

References

1. Rhodes A, Evans LE, Alhazzani W, Levy MM, Antonelli M, Ferrer R, et al. Surviving sepsis campaign: International guidelines for management of sepsis and septic shock: 2016. Intensive Care Med. 2017;43(3):304-377.

**Table 1 (abstract P2).** ROC curve analysis identify cut-off point of lactate to predict hospital outcome

Parameter	AUC(95%CI)	Cut-off point	Sensitivity	Specificity	Youden’s index
Non-cirrhosis					
-Baseline lactate	0.63(0.57-0.66)	4	0.5	0.66	0.16
-Following lactate	0.62(0.56-0.68)	2	0.8	0.44	0.24
-Lactate clearance	0.56(0.50-0.62)	10	0.7	0.43	0.13
Cirrhosis					
-Baseline lactate	0.65(0.44-0.85)	5	0.78	0.61	0.39
-Following lactate	0.60(0.35-0.84)	5	0.67	0.68	0.35
-Lactate clearance	0.54(0.29-0.78)	20	0.52	0.56	0.08

## P3 Septic shock outcomes among octogenarians

### Chairat Permpikul, Surat Tongyoo

#### Division of Critical Care Medicine, Department of Medicine, Faculty of Medicine Siriraj Hospital, Mahidol University, Bangkok, Thailand

##### **Correspondence:** Surat Tongyoo (surat.ton@mahidol.ac.th)

Background

Thailand is moving toward aged society. It is interesting whether octogenarian, elderly aged 80 years old or older, with septic shock has different clinical characteristics and outcomes as compared with younger patients. We report here the information from our patient cohort.

Materials and methods

This is a retrospective study, included adult septic shock patients who were admitted at Siriraj hospital between April 2011 and December 2017. Patients’ baseline information, severity score, treatment modality and outcomes were retrieved. Octogenarian was defined by the age of 80 years or older at the day of admission. All patients received septic shock resuscitation, following standard guideline.1 The primary outcome was 30 days mortality.

Results

A total of 782 septic shock patients were enrolled. Of these 133 were 80-year-old or older. When compared with younger patients, octogenarian had lower APACHE II score and higher comorbidities which included: hypertension, coronary artery disease, previously stroke, cirrhosis, and chronic kidney disease. Pneumonia was the leading site of infection in both groups but occurred in higher proportion in octogenarian (46.6% vs 29.7%, P 80 years old as the independent predictor of death within 30 days, as well as other parameters as shown in Table 1.

Conclusions

Octogenarian with septic shock had lower disease severity but higher comorbidities. This group had higher mortality, both at 30 day and at discharge.

References

1. Rhodes A, Evans LE, Alhazzani W, Levy MM, Antonelli M, Ferrer R, et al. Surviving sepsis campaign: International guidelines for management of sepsis and septic shock: 2016. Intensive Care Med. 2017;43(3):304-377.

**Table 1 (abstract P3).** Multivariate analysis for predictive factor associated with 30-day mortality

Parameter	Relative risk	95% confidence interval	P value
Age > 80 years old	2.23	1.01-4.96	0.05
Cirrhosis	5.05	1.91-13.38	0.001
Pneumonia	2.00	1.01-4.05	0.05
Positive hemoculture	3.10	1.46-6.55	0.003
Ventilator support	4.91	2.29-10.55	<0.001
Adrenaline requirement	3.02	1.11-8.20	0.03

## P4 Effect of low dose corticosteroid in septic shock resuscitation: sub-group analysis result of a randomized controlled trial

### Surat Tongyoo, Chairat Permpikul

#### Department of Medicine, Faculty of Medicine, Siriraj Hospital, Mahidol University, Bangkok, Thailand

##### **Correspondence:** Surat Tongyoo (surat.ton@mahidol.ac.th)

Background

Efficacy of low dose hydrocortisone administrated during septic shock resuscitation had been long debated. Despite recent two large randomized controlled trials, steroids benefit is still inconclusive [1,2]. The aim of this study is to evaluate the effect of hydrocortisone on resuscitation outcomes, comparing between hydrocortisone and placebo.

Materials and methods

This is a retrospective study, included participants who were enrolled in the previously reported randomized controlled trial of hydrocortisone treatment in early sepsis-associated acute respiratory distress syndrome [3]. The patients who met criteria of septic shock, according to Sepsis-3 definition [4] were included in sub-group analysis. The treatment group received hydrocortisone 50 mg intravenously every 6 hours for 7 days, while the control group received normal saline in identical volume. The primary outcome was rate of survival without organ support at 28 days after septic shock diagnosis.

Results

Of 197 patients enrolled in the previous study, 154 patients met septic shock criteria. Seventy-eight patients were randomized to receive hydrocortisone and 76 patients were in the placebo group. The patients’ age, gender, underlying conditions, source of infection, baseline arterial pressure and severity score were not different. Mean APACHE II score was 22.3+5.8, mean SOFA score was 11.6+3.1 and mean initial lactate was 4.0+2.4 mmol/L. The rate of survival with no organ support at 28 days was non-significantly higher in hydrocortisone group (61.5% vs 48.7%, relative risk 1.28 [95%CI 0.93-1.76]; P=0.12). The secondary outcomes, as shown in Table 1, were not different; except vasopressor dependent day which was shorter in hydrocortisone group (4.8+3.0 vs. 6.8+5.7 days; P=0.008). As for adverse events, hyperglycemia occurred more in hydrocortisone group (83.8% vs. 68.9%; P=0.03) while the rate of re-infection, cardiac arrhythmia and gastrointestinal bleeding was the same.

Conclusions

Hydrocortisone administration during septic shock resuscitation associated with shorter vasopressor dependent day without any benefit in other outcomes.

References

1. Annane D, Renault A, Brun-Buisson C, Megarbane B, Quenot JP, Siami S, Cariou A, Forceville X, Schwebel C, Martin C, et al. Hydrocortisone plus fludrocortisone for adults with septic shock. New Engl J Med 2018, 378: 809–818.

2. Venkatesh B, Finfer S, Cohen J, Rajbhandari D, Arabi Y, Bellomo R, Billot L, Correa M, Glass P, Harward M, et al. Adjunctive glucocorticoid therapy in patients with septic shock. N Engl J Med 2018, 378:797–808.

3. Tongyoo S, Permpikul C, Mongkolpun W, Vattanavanit V, Udompanturak S, Kocak M, Meduri GU. Hydrocortisone treatment in early sepsis associated acute respiratory distress syndrome: results of a randomized controlled trial. Crit Care 2016, 20: 329.

4. Singer M, Deutschman CS, Seymour CW, Shankar-Hari M, Annane D, Bauer M, Bellomo R, Bernard GR, Chiche JD, Coopersmith CM, et al. The Third International Consensus Definitions for Sepsis and Septic Shock (Sepsis-3). JAMA 2016, 315: 801-810.

**Table 1 (abstract P4).** Primary and secondary outcome comparing between hydrocortisone and placebo groups

Outcome	Placebo *(n=76)*	Hydrocortisone *(n=78)*	Relative Risk *(95%CI)*	P
Primary outcome: Survive with no organ support at 28 day (%)	37 (48.7)	48 (61.5)	1.28 (0.93-1.76)	0.12
Secondary outcome 28 day mortality (%)	24 (31.6)	19 (24.4)	0.78 (0.59-1.18)	0.32
Hospital mortality (%)	35 (46.1)	33 (42.3)	0.93 (0.67-1.33)	0.64
Ventilator dependent days	14.6+9.3	12.0+8.0		0.07
Days survive without ventilator in 28 days	9.2+9.7	12.1+9.4		0.07
Vasopressor dependent days	6.8+5.7	4.8+3.0		0.008
Days survive without vasopressor in 28 days	17.1+9.4	19.3+8.5		0.12
Renal replacement therapy (%)	19 (25)	21 (26.9)	1.05 (0.70-1.57)	0.79
RRT dependent days	2.1+4.5	2.1+4.8		1.0

## P5 THAI-ICU score as a simplified severity score for critically ill patients: result from SEA-AKI study group

### Theerapon Sukmark^1^, Kearkiat Praditpornsilpa^2^, Kriang Tungsanga^2^, Kamol Khositrangsikun^3^, Petchdee Oranrigsupak^4^, Somchai Eiam-ong^2^, Nattachai Srisawat^2,5^

#### ^1^Thungsong Hospital, Nakhon Si Thammarat, Thailand; ^2^Division of Nephrology, Department of Medicine, Faculty of Medicine, Chulalongkorn University, Bangkok, Thailand; ^3^Maharaj Nakhon Si Thammarat hospital, Nakhon Si Thammarat, Thailand; ^4^Nan hospital, Nan, Thailand; ^5^Excellence Center for Critical Care Nephrology, Thai Red Cross, Bangkok, Thailand

##### **Correspondence:** Theerapon Sukmark (theerapon_s@hotmail.com)

Background

Intensive care unit (ICU) scoring systems is commonly used worldwide to predict ICU outcome. However, most of standard scores usually need many variables, even some sophisticated laboratory parameters or software to calculate. In resource limited setting, this is one of the major barriers to apply in clinical practice. The objective of this study was to create a simplified ICU scoring system to predict mortality in critically ill patients.

Materials and methods

A prospective multicenter website-based-data-collection cohort, involving the adult patients who admitted to ICU of 17 centers across Thailand during 2013 to 2015. A development cohort and a validation cohort were randomly selected from available enrollment data. The simplified score was created by multivariable regression-based method. Receiver-Operating Characteristic (ROC) analysis was used for evaluating discrimination performance[1]. Hosmer-Lemeshow (H/L) - C statistic and calibration curve were performed to calibration evaluation[2].

Results

In development cohort; 3,458 cases were in analysis. Among those, 2,458 (71 %) and 1,000 (29 %) cases were alive and dead in hospital course, respectively (Fig. 1).

Adjusted odds ratios (95% CI) of predictors including low Glasgow coma score (GCS 10-14), very low GCS (<10), low blood pressure (Mean arterial pressure < 70 mm Hg or need vasopressor), over positive net fluid balance (>1,500 cc), tachypnea (respiratory rate > 24 per minute), low platelet count (<150,000 / *μ*L), and high BUN (BUN > 20 mg/dL) were 4.2 (3.1-5.6), 12.9 (9.4-17.8), 2.3 (1.9-2.8), 1.3 (1.1-1.6), 1.6 (1.3-1.9), 1.9 (1.6-2.3), and 1.8 (1.5-2.2), respectively (Fig. 2).

In simplified THAI-ICU score (6 variables) model, the scores of those predictors were 4, 13, 2.5, 1.5, 1.5, 2, and 2, respectively (Table 1).

In validation cohort of THAI-ICU score model (6 variables); 1,880 cases were in analysis. The AUC (95%CI) of THAI-ICU, APACHE II, and SOFA score were 0.81 (0.78-0.83), 0.76 (0.74-0.79), and 0.77 (0.75-0.80), respectively (Fig. 3).

For discrimination evaluation, at the cutoff value equal to 9 of THAI-ICU score; the sensitivity, specificity, positive likelihood ratio was 72 %, 73 %, and 2.72, respectively (Table 2). For calibration, H/L- C statistic was 13.5, p = 0.2. And also, Brier score (95% CI) was 0.16 (0.15, 0.17) (Fig. 4, Table 2).

Conclusions

We developed a simplified THAI-ICU score which outperformed the standard severity score and can be considered as a powerful tool to predict hospital mortality. The simplicity of THAI-ICU score will increase the possibility to feasibly apply in resource limited setting.

Acknowledgment

The study was on behalf and supported by SEA-AKI study group; Dr. Nattachai Srisawat as the research group leader.

References

1. Bewick V, Cheek L, Ball J. Statistics review 13: receiver operating characteristic curves. Crit Care. 2004;8(6):508-512.DOI: 10.1186/cc3000.PMC1065080.

2. Labarère J, Bertrand R, Fine MJ. How to derive and validate clinical prediction models for use in intensive care medicine. Intensive care medicine. 2014;40(4):513-527.DOI: 10.1007/s00134-014-3227-6

3. Granholm A, Perner A, Krag M, Hjortrup PB, Haase N, Holst LB, Marker S, Collet MO, Jensen AK, Møller MH. Development and internal validation of the Simplified Mortality Score for the Intensive Care Unit (SMS‐ICU). Acta Anaesthesiologica Scandinavica. 2018 Mar;62(3):336-346.

4. Haniffa R, Mukaka M, Munasinghe SB, De Silva AP, Jayasinghe KS, Beane A, de Keizer N, Dondorp AM. Simplified prognostic model for critically ill patients in resource limited settings in South Asia. Critical Care. 2017 Dec;21(1):250.

5. Riviello ED, Kiviri W, Fowler RA, Mueller A, Novack V, Banner-Goodspeed VM, Weinkauf JL, Talmor DS, Twagirumugabe T. Predicting mortality in low-income country ICUs: the Rwanda Mortality Probability Model (R-MPM). PloS one. 2016 May 19;11(5):e0155858.

**Fig. 1 (abstract P5).**
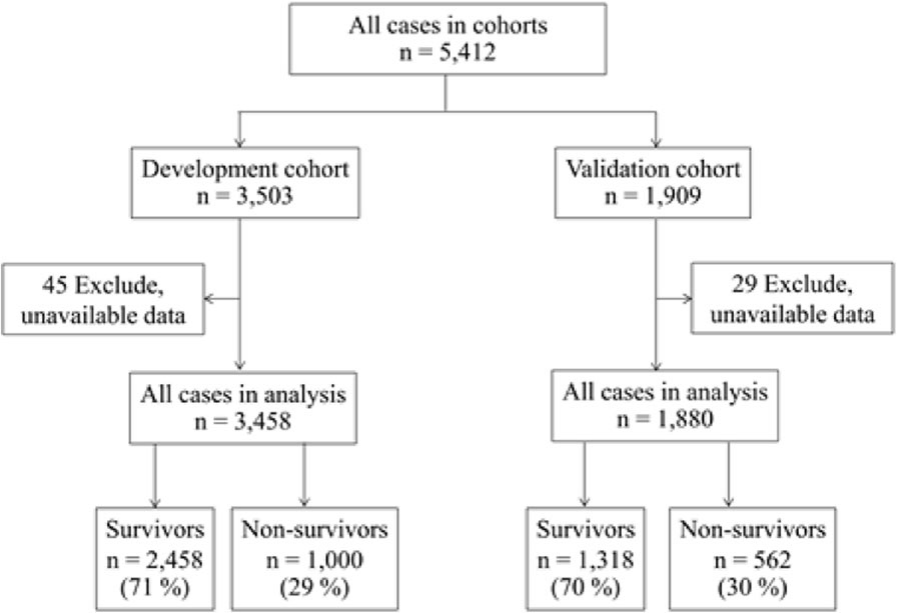
Flow chart of subject disposition for the THAI-ICU Score study, randomly into development cohort and validation cohort

**Fig. 2 (abstract P5).**
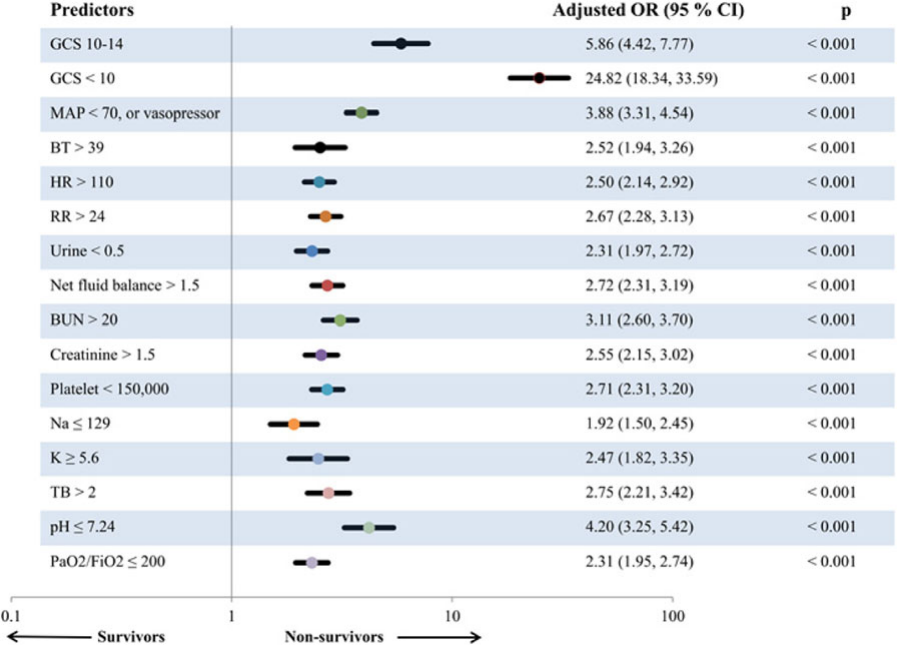
Forest plot in log-10 scale of odds ratio (adjusted for age, gender, body mass index and co-morbidities; including hypertension, diabetes, coronary artery disease, cerebrovascular disease, malignancy, chronic kidney disease) in prediction of non-survivor

**Table 1 (abstract P5).** Creating the THAI-ICU score of development cohort with multivariable regression-based method (n=3,458)

Predictors	6 variables Model			8 variables Model		
	B	Adjusted odds ratio^i^ (95% CI)	Score	B	Adjusted odds ratio^i^ (95% CI)	Score
^a^GCS 10-14	1.43	4.16 (3.10, 5.60)	4.0	1.39	4.03 (2.99, 5.41)	4.0
< 10	2.56	12.94 (9.40, 17.81)	13.0	2.48	11.99 (8.69, 16.54)	12.0
^b^MAP < 70	0.85	2.33 (1.93, 2.81)	2.5	0.81	2.242 (1.85, 2.71)	2.0
^c^Fluid > 1.5	0.26	1.29 (1.06, 1.57)	1.5	0.22	1.25 (1.02, 1.52)	1.0
^d^RR > 24	0.46	1.58 (1.31, 1.90)	1.5	0.42	1.53 (1.26, 1.84)	1.5
^e^Plt < 150,000	0.65	1.91 (1.58, 2.31)	2.0	0.58	1.79 (1.47, 2.19)	2.0
^f^BUN > 20	0.59	1.81 (1.49, 2.19)	2.0	0.57	1.76 (1.46, 2.14)	2.0
^g^TB > 2	-	-	-	0.34	1.40 (1.07, 1.84)	1.5
^h^pH ≤ 7.24	-	-	-	0.52	1.68 (1.24, 2.28)	1.5
Maximum score	-	-	22.5	-	-	23.5

^a^GCS; Glasgow coma score, ^b^MAP; mean arterial pressure (mm Hg), ^c^Fluid; positive net fluid balance (liters), ^d^RR; respiratory rate (beats per minute), ^e^Plt; platelet (cell per microliter),

^f^BUN; blood urea nitrogen (mg/dL), ^g^TB; total bilirubin (mg/dL), ^h^pH; arterial blood pH

^i^adjusted odds ratio for covariates

**Fig. 3 (abstract P5).**
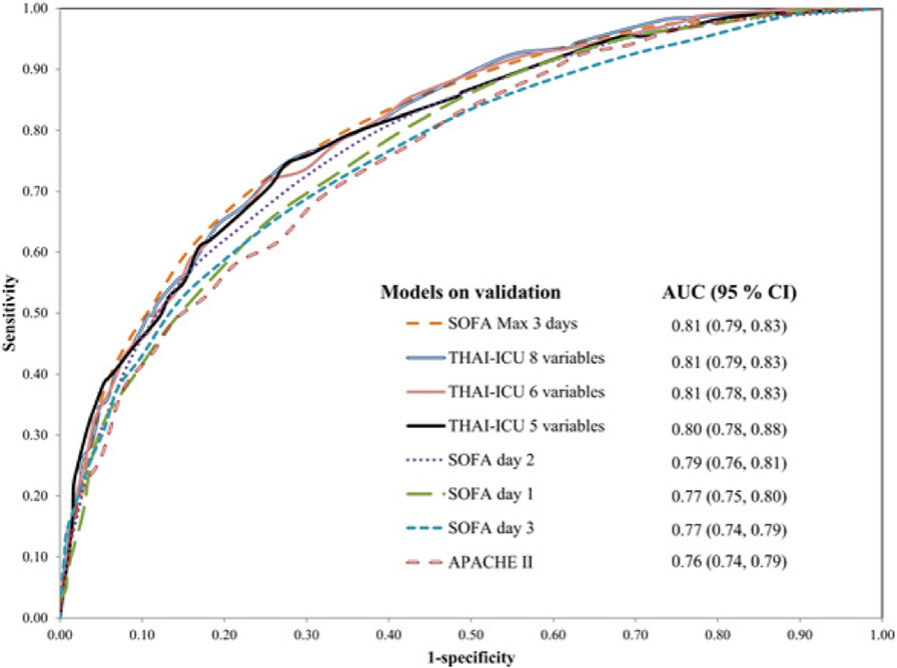
The area under the receiver operating characteristic (ROC) curve for mortality prediction of each model of simplified THAI-ICU scores, comparing with APACHE II and SOFA in validation cohort. AUC; area under ROC curve

**Fig. 4 (abstract P5).**
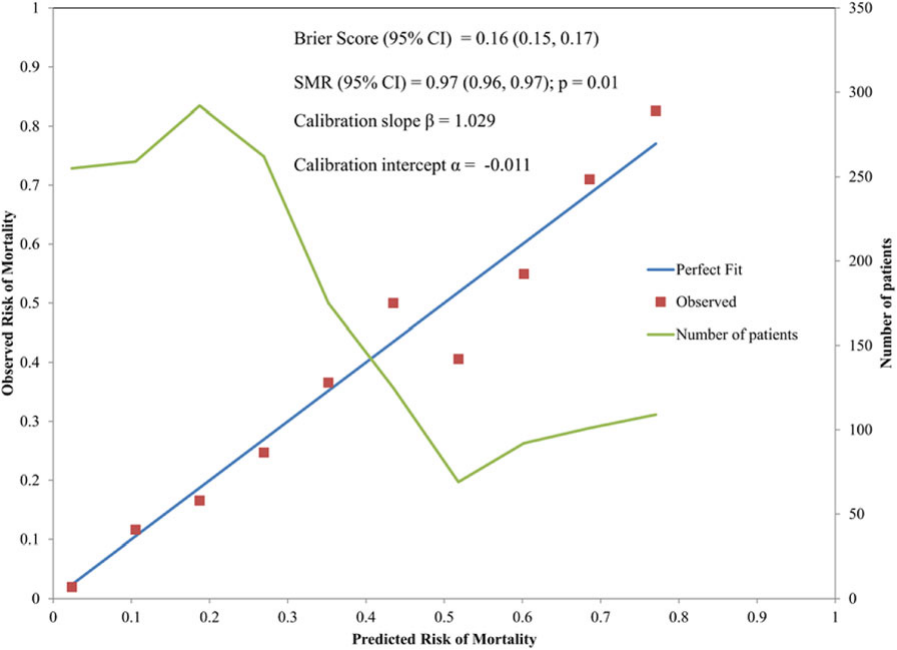
Calibration curve comparing between observed and predicted mortality at each cut-point of THAI-ICU score 6 variables in validation cohort

**Table 2 (abstract P5).** Performance comparison of recently available simplified-ICU severity scores

Model	THAI-ICU 2018	SMS-ICU 2018 [3]	Haniffa et al’s model, 2017 [4]	R-MPM 2016 [5]
Geographic regions	Thailand	Denmark, European countries, Brazil, Canada, Australia	Sri Lanka, India, Nepal, Bagladesh	Rwanda
Centers	17	5 international, multicenter studies and RCTs	21	2
Predicted outcome	Hospital mortality	90-day mortality	ICU mortality	Hospital mortality
Total study population, n	5,412	4,086	3,855	427
Variables	6	7	6	5
AUC (95% CI)	0.81 (0.78, 0.83)	0.72 (0.71, 0.74)	0.77 (0.73, 0.80)	0.81
Sensitivity (%)	72	N/A	70	N/A
Specificity (%)	73	N/A	69	N/A
H/L C- statistic (p)	13.5 (p = 0.20)	9.04 (p = 0.34)	11.31(p = 0.19)	11.94 (p = 0.15)
Nagelkerke’s R^2^	0.323	0.19	N/A	N/A
Brier Score (95% CI)	0.16 (0.15, 0.17)	N/A	0.14 (0.12, 0.15)	0.18

## P6 The role of novel immunologic biomarkers in prediction of outcomes in severe sepsis/septic shock patients

### Chalermchai Komaenthammasophon^1,2^, Nuttha Lumlertgul^1,2^, Sasipha Tachaboon^1,2^, Janejira Dinhuzen^1,2^, Sadudee Peerapornratana^1,2^, Nattachai Srisawat^1,2^

#### ^1^Division of Nephrology, Department of Internal Medicine, Faculty of Medicine, Chulalongkorn University, Bangkok, Thailand; ^2^Excellence Center for Critical Care Nephrology, King Chulalongkorn Memorial Hospital, Bangkok Thailand

##### **Correspondence:** Nuttha Lumlertgul (nlumlertgul@gmail.com)

Background

Innate immune response is an important host response to infection. However, the role of neutrophil chemotaxis activity, CD11b expression on neutrophils, and monocyte HLA-DR (mHLA-DR) expression as novel prognostic biomarkers in patients with severe sepsis/septic shock is still unknown. The purpose of this study was to evaluate the discriminatory characteristics of these biomarkers on mortality.

Materials and methods

This prospective cohort study was conducted at intensive care units, King Chulalongkorn Memorial hospital. Patients with severe sepsis or septic shock were enrolled. We collected blood samples at the time of enrollment to test immunologic biomarkers. Neutrophil function was evaluated by measurement of neutrophils chemotaxis activity and CD11b expression. Monocyte function was assessed by the measurement of mHLA-DR expression, a key marker of immunoparalysis state, and presepsin level. The primary end point was 28 day-mortality.

Results

A total of 136 participants were enrolled. Patients were classified by mortality at day 28 into 2 groups as survivors group (n=59, 43%) and non-survivors group (n=77, 57%). Neutrophil chemotaxis activity was significantly higher in survivors group than non-survivors group (46.7% vs. 41.4%, p=0.027). There was significantly lower CD11b expression on neutrophils in survivors than non-survivors (10.3% vs. 14.8%, p=0.012). There were no significant differences in mHLA-DR expression and presepsin level between survivors and non-survivors. Neutrophil chemotaxis activity predicted 28-day mortality with an area under the receiver operating characteristic curve (AUC-ROC) of 0.76 (95%CI 0.67-0.85), while the HLA-DR, CD-11b expression, and presepsin showed the AUC-ROC of 0.69 (0.60-0.79), 0.71 (0.61-0.80), and 0.62 (0.51-0.72), respectively. Neutrophil chemotaxis activity at the cutoff of 49% showed the sensitivity of 0.51, and specificity of 0.71. By stepwise analysis, combining neutrophil chemotaxis activity with mHLA-DR, CD11b expression, presepsin, and SOFA score provided the highest AUC-ROC of 0.84 (0.77-0.91) in predicting 28-day mortality.

Conclusions

Neutrophil chemotaxis activity appeared to be a promising novel immunologic biomarker in predicting 28-day mortality in patients with severe sepsis or septic shock.

Acknowledgements

The investigator-initiated study was funded by an unrestricted research grant from Ratchadapiseksomphot endowment fund, Faculty of Medicine, Chulalongkorn University. Statistical analysis was conducted by Miss Pimnara Peerawaranun.

## P7 A case report of *Pasteurella multocida* sepsis incidentally found with multiple myeloma

### Takanori Sekito^1^, Masami Ishikawa^*2*^

#### ^1^Department of General Medicine, Kure Kyosai Hospital, Hiroshima, Japan; ^2^Department of Emergency and Critical Care Medicine, Kure Kyosai Hospital, Hiroshima, Japan

##### **Correspondence:** Takanori Sekito (t.sekito410@gmail.com)

Background

*Pasteurella multocida* (PM) is a common cause of zoonotic infection following bites or scratches from dogs or cats. However, PM sepsis rarely occurs in healthy adults. We encountered a case of PM sepsis probably due to multiple myeloma (MM).

Patient

A 69-year-old female was transported to the emergency room with symptoms relating to polymyalgia and polyarthralgia. On first presentation, she reported no notable past medical history, including MM. Clinical course: She was alert and oriented, with vital signs as follows: blood pressure, 99/59 mmHg; heart rate, 102 bpm; respiratory rate, 34/min; SpO2 92% (room air); and body temperature, 38.9 °C. Hematological investigations indicated anemia, a low platelet and leukocyte count, an elevated D-dimer value and an elevated C-reactive protein level. Based on these results, she was diagnosed with sepsis and disseminated intravascular coagulation (DIC) and admitted into the intensive care unit for general management. Meropenem were initially administrated for sepsis and Danaparoid sodium was administered to treat the DIC. A blood cultivation test grew PM and the antibiotic prescription was tailored to Cefazolin. Electrophoresis was performed and showed IgG κ type M-protein during hospitalization. Because her fever recurred, the antibiotics were tailored to Ceftriaxone. Her vital signs gradually normalized and her general condition, including the polymyalgia and polyarthralgia, gradually improved. On hospital day 29, she had resumed activities of daily living and was discharged, with anemia and M-proteinemia remaining. Post-discharge, bone marrow aspiration was performed at follow-up showing more than 35% of plasma cells and symptomatic MM was diagnosed.

Discussion and conclusion

PM is usually carried by cats and dogs. PM infection in humans is often associated with direct animal contact such as an animal bite, scratch, lick, or kiss, and PM is likely to occur in older people and immunocompromised hosts. Our patient had a cat at home but denied any history of a cat bite. She was a compromised host because of the MM which likely led to the PM infection and sepsis. Possible underlying immunocompromised conditions should be considered when diagnosing PM sepsis, even where patients have no obvious past medical history on initial presentation.

Consent

Written informed consent was obtained from the patient for publication of this case report and accompanying images. A copy of the written consent is available for review by the Editor-in-Chief on request.

## P8 Diagnostic and treatment of bone and joint sepsis in newborns

### Gennadiy Khanes^1^, Tetiana Ivanova^1^, Olga Lyutko^2^

#### ^1^Ukrainian National Specialized Pediatric Hospital OkhMatDyt, Kyiv, Ukraine; ^2^Research Institute of Orthopedics of Medical Sciences of Ukraine, Kyiv, Ukraine

##### **Correspondence:** Gennadiy Khanes (dhanes188@gmail.com)

Background

Bone and joint sepsis (osteomyelitis and arthritis) account for up to 20% of all purulent-septic pathology in newborns. Studies of the last 10 years show that in the development of bone-joint sepsis an important role is played by intranatal and contamination infection, which leads to coagulation disorders. An important role in the development of bone-joint sepsis in infants is played by (physiological) immunodeficiency and, as a consequence, immunity disorders.

Materials and methods

For 10 years (2007-2017), 300 newborns with bone-joint sepsis were treated. The leading role in the diagnosis of bone-joint neonatal sepsis is played by the evaluation of the inflammatory process with the help of general tests of CRP, PCT, a general blood test - % monocytes and coagulation disorders.

Locally - an assessment of the inflammatory process with the help of an ultrasound examination and an LE-test. From 12-14 days of the disease, the x-ray examination retains its significance.

Results

In 44.1% of patients we have positive microbiological tests, wherein a variety of isolated Staphylococcus and Streptococcus, which are 100% of the sensitive glycopeptide. MSSA also sensitive to rifampicin, amoksiklav and cefoxitin. Enterococci are sensitive to glycopeptides, linezolid, piperocillin / tazobactam, amoksiklav. The greatest resistance to antibiotics showed *Enterococcus faecium* and Bacillus spp.

Conclusions

In the success of the treatment of bone-joint sepsis is adequately selected in the first hours of admission antibiotic therapy (the first three hours - a cure of 98%). Staphylococci are the main etiological factor of bone and joint sepsis in young children. To clarify the etiology of the disease serology are important.

## P9 Development and validation of nurse-directed weaning protocol in a mixed ICU setting - Theptarin weaning protocol

### Plianpan Panitta^1^, Trakarnvijitr Sirireudee^1^, Chongartklang Saifon^1^, Wongasa Patsaraporn ^1^, Ketwaengkuang Puntisa^1^, Chotjirat Anocha^1^, Thewjitcharoen Yotsapon^2^, Himathongkam Thep^2^

#### ^1^Intensive Care Unit, Theptarin Hospital, Bangkok, Thailand; ^2^Department of internal medicine, Theptarin Hospital, Bangkok, Thailand

##### **Correspondence:** Plianpan Panitta (kobicu08@gmail.com)

Background

Prolonged mechanical ventilation of critically ill patients is associated with adverse clinical outcomes. Therefore, mechanical ventilation should be discontinued as soon as patients are capable of breathing independently. Studies have shown that weaning protocols lead to a decrease in duration of mechanical ventilation and complications associated with mechanical ventilation. This study aims to develop and validate a weaning protocol to use in a mixed ICU setting based on 4 components to achieve successful weaning process from the first step of readiness to wean criteria until the final step of structured monitoring after extubation.

Materials and methods

A nurse-directed protocol for weaning from mechanical ventilator (use only in patients not to expect extubation within 48 hours) was developed based on extensive literature review and then three critical care physicians validated the content of each parameter. The Theptarin weaning protocol composed of 4 components (readiness, during weaning, before ex-tubation, and after ex-tubation) divided in 27 items. Finally, the protocol was evaluated in validation analysis with a retrospective cohort of patients who required mechanical ventilation more than 48 hours in the ICU of Theptarin hospital (6 beds mixed ICU setting) from 2013-2018.

Results

In our retrospective cohort of 96 patients (females 58%, mean age 78.6±12.3 yrs, median APACHE II scores 18, median length of ICU stay 13 days), the successful in the first attempt extubation was achieved in 76.0%. In patients who violated the pre-determined readiness to wean criteria could successfully weaning only 60% when compared with 100% in patients who fulfilled all criteria for the first component of Theptarin weaning protocol. The sensitivity, specificity, negative predictive value, and positive predictive value of the protocol were 53.4%, 100%, 59.6%, and 100%, respectively. Regarding the readiness to wean criteria, the laboratory item tended to less predict for failure of weaning process when compared with other items.

Conclusions

Our validated Theptarin weaning protocol could identify patients who were ready to wean from mechanical ventilator in a timely manner and could be applied in a mixed ICU setting with empowerment of ICU nurses. The implementation of this simple nurse protocol into the routine weaning practice and periodic audits should be done to demonstrate outcome benefits for patients.

## P10 The levels of plasma Calprotectin and other biomarkers in early sepsis

### Aleksandra Havelka^1,2^, Anders Larsson^3^, Joachim Johansson^4^, Jonas Tydén^4^, Miklos Lipcsey^5^

#### ^1^Department of Molecular Medicine and Surgery, Karolinska Institute, Stockholm, Sweden; ^2^Gentian Diagnostics AB, Stockholm, Sweden; ^3^Department of Medical Sciences, Uppsala University, Uppsala, Sweden; ^4^Department of Anaesthesia and Intensive Care Östersund, Umeå University, Umeå, Sweden; ^5^Department of Surgical Sciences, Anaesthesiology and Intensive Care, Uppsala University, Uppsala, Sweden

##### **Correspondence:** Aleksandra Havelka (aleksandra.havelka@gentian.no)

Background

Sepsis is a condition with high mortality where early treatment improves outcome suggesting that rapid identification of patients with sepsis is important. Calprotectin is a novel biomarker that can be analysed in routine laboratory with short turnaround times. We set out to determine the levels of plasma Calprotectin in patients with and without sepsis on intensive care (ICU) admission and as a marker of mortality day 30 post ICU admission.

Materials and methods

All adult patients with arterial cannula that where primarily admitted to the mixed ICU at Östersund General Hospital between the 1st of February 2012 and 31st of January 2013 were screened for discharge diagnoses. Plasma taken on admission from patients was analysed for Calprotectin, and the more investigated sepsis biomarkers Heparin Binding Protein (HBP) and Procalcitonin. We compared levels of these biomarkers in patients with sepsis (defined according to Sepsis 2) with levels in patients admitted for trauma, i.e. controls with inflammation, patients with other medical conditions (OMC), i.e. medical admissions with no infection, and finally with medical and surgical patients presenting without these pre-specified diagnoses (Miscellaneous conditions).

Results

328 patients met the inclusion criteria. 83 patients had sepsis, 33 trauma, 77 OMC as discharge diagnosis and 118 Miscellaneous conditions. Mortality at 30 days was 20% for sepsis patients, 9% for trauma patients, 21% for patients with OMC and 23% for patients with Miscellaneous conditions. The levels of Calprotectin were higher in sepsis vs. trauma patients (p<0.001, Fig. 1), sepsis vs. OMC (p<0.01), sepsis vs. Miscellaneous conditions (p<0.01) and was higher in patients who did not survive to 30 days (p<0.01). HPB levels were higher in sepsis vs. trauma patients (p<0.001, Fig. 2), sepsis vs. OMC (p<0.001), but were comparable sepsis vs. Miscellaneous conditions (n.s.). HBP was higher in patients that were dead at 30 days (p<0.001). Plasma Procalcitonin did not differ between the groups or for outcome (Fig. 3).

Conclusions

Calprotectin may be superior to Procalcitonin and HBP in indicating patients with sepsis as Calprotectin, unlike HBP and Procalcitonin, was consistently higher in sepsis patients compared to patients with other diagnoses with and without systemic inflammation. Calprotectin and HBP showed predictive ability regarding 30 days mortality. Procalcitonin did not.

**Fig. 1 (abstract P10).**
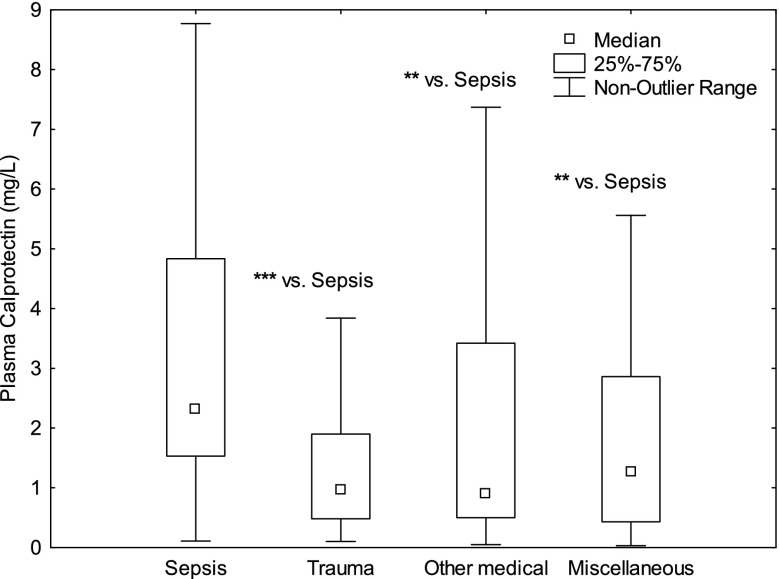
See text for description

**Fig. 2 (abstract P10).**
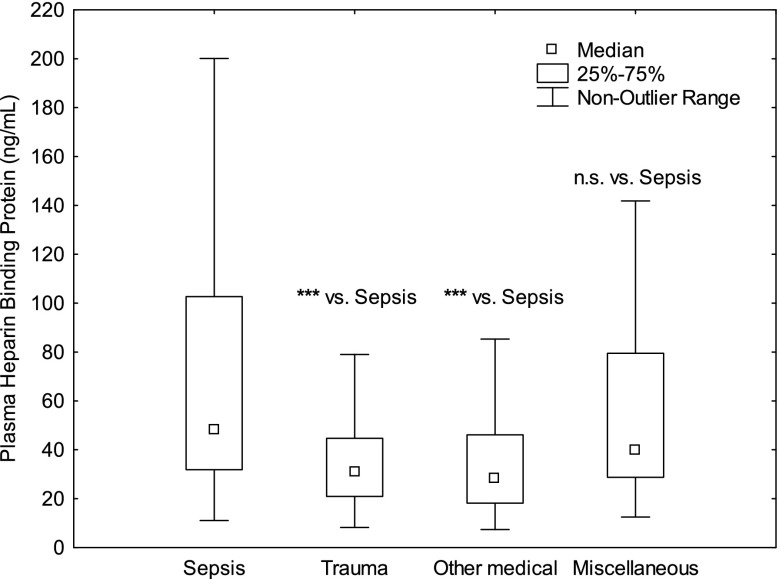
See text for description

**Fig. 3 (abstract P10).**
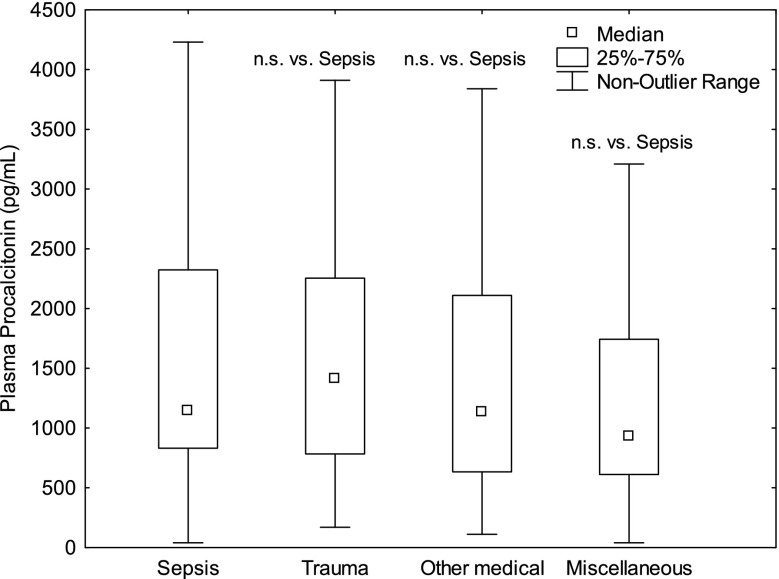
See text for description

## P11 Prognostic value of DNA-traps level in blood smears from patients with sepsis

### Darya V Mosalskaya^1^, Alexander S Gur’ev^1,2^, Andrey F Lopatin^1^

#### ^1^M.F.Vladimirsky Moscow Regional Clinical and Research Institute (MONIKI), scientific-research laboratory, Moscow, Russian Federation; ^2^Medtechnopark LTD, Moscow, Russian Federation

##### **Correspondence:** Andrey F Lopatin (coherneph@mail.ru)

Background

In 2004 a novel suicidal antimicrobial strategy of human neutrophils was described, named NETosis (NET – Neutrophil Extracellular Trap) [1]. NETosis is the particular case of ETosis (ET – Extracellular Trap), which is specific for a variety of human phagocytes. During ETosis phagocytes are transformed, resulting in subcellular structures formation, such as so-called extracellular DNA-traps (DTs), consisted of nuclear DNA with immobilized antimicrobial proteins and enzymes. DTs efficiently bind and kill microorganisms, however they are toxic to host cells. DTs play important role in thromboses, inflammatory and autoimmune diseases [2]. DTs are known to be important in the pathogenesis of sepsis [3]. At the same time standard methods for determination of ETosis-transformed phagocytes in human blood are not developed yet [4]. New method [5] was proposed for ETosis-transformed phagocytes determination in blood of patients with sepsis, based on DTs calculation in standardized thin blood smears. However, additional studies are needed to confirm its clinical usefulness.

The aim of this study is to evaluate the connection between increased DTs level (in blood smears from patients with sepsis) with outcome of the disease.

Materials and methods

DTs levels were determined in blood smears of 51 patients in intensive care unit with verified sepsis. 55 healthy donors were in control group. Peripheral venous blood was collected into tubes containing EDTA, standardized thin blood smears were prepared using 2 μl of blood, and stained with Giemsa stain. Levels of DTs were calculated using automated microscopy system MECOS-C2 (Moscow, Russia). Blood of the patients was studied several times during staying in intensive care unit; the maximal DTs levels during all time of observation were analyzed.

Results

Among 51 patients with sepsis 20 died and 31 recovered. The most informative indicator was the maximal DTs level during all time of observation. In group of all patients this indicator was in average 15.1% (σ=14.9%, Min-Max: 1–72%). Died patients – 25.3% (σ=18.9%, Min-Max: 5.6%–72%). Recovered patients – 8.7% (σ=5.3%, Min-Max: 1%–22.5%). Healthy donors – 5.7% (σ=2.5%, Min-Max: 1.8%–11.9%). The differences between the groups were statistically significant according to Mann-Whitney U-test: donors vs septic patients – p=0.000026*; recovered vs died patients – p=0.01*** (Fig. 1).

Conclusions

In average, maximal DTs level was significantly higher in patients with sepsis than in healthy donors. Besides, maximal DTs level was significantly higher in died patients than in recovered patients. These results allow to conclude, that high level of DTs in patients with sepsis correlates with lethal outcome.

References

1. Brinkmann V, Reichard U, Goosmann C, Fauler B, Uhlemann Y, Weiss DS, Weinrauch Y, Zychlinsky A: Neutrophil extracellular traps kill bacteria. Science 2004, 303(5663):1532-1535.

2. Papayannopoulos V: Neutrophil extracellular traps in immunity and disease. Nat Rev Immunol 2018, 18(2):134-147.

3. O'Brien X, Biron B, Reichner J: Consequences of extracellular trap formation in sepsis. Curr Opin Hematol 2017, 24(1):66-71.

4. Masuda S, Nakazawa D, Shida H, Miyoshi A, Kusunoki Y, Tomaru U, Ishizu A: NETosis markers: Quest for specific, objective, and quantitative markers. Clin Chim Acta 2016, 459:89-93.

5. Gur’ev A, Mosalskaya D, Lopatin A, Rusanova E, Vasilenko I, Volkov A. Determination of phagocytes, transformed during etosis, by morphological method in patients with sepsis. High medical technologies in XXI century: materials of the round table 2017, 57.

**Fig. 1 (abstract P11).**
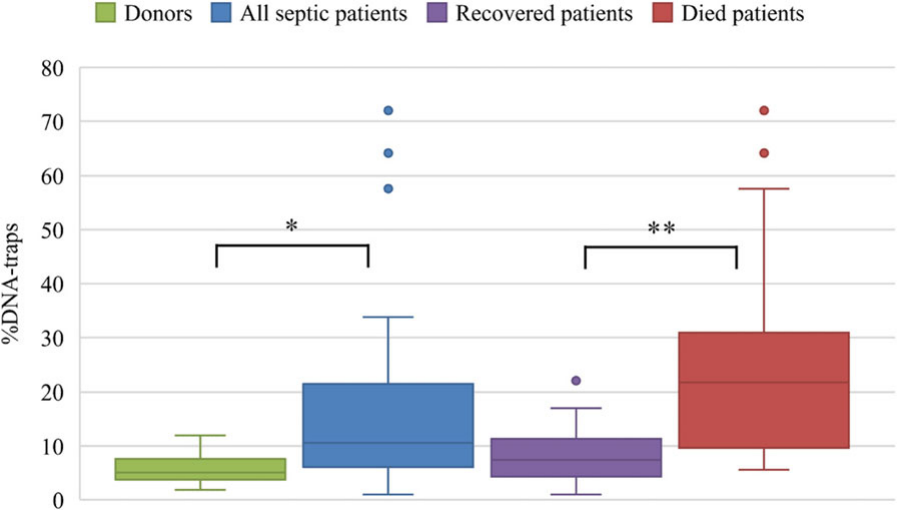
Maximal DTs levels during all time of observation in blood smears from patients with sepsis and healthy donors

## P12 Monitoring the activity of antithrombin in sepsis allows predicting the likelihood of a clinical outcome of the disease

### Ivan V Redkin, Andrey F Lopatin, Vera V Samoylenko, Yury V Skripkin, Valery V Likhvantsev

#### Moscow Regional Scientific Research Clinical Institute, Russia, Moscow

##### **Correspondence:** Audrey Lopatin (mdalopatin@hotmail.com)

Background

Disturbance of hemostasis plays an important and sometimes decisive role in the development of multiple organ failure and the clinical outcome of sepsis. The question - "What diagnostic criterion of hemostasis can be most useful for assessing the dynamics and outcome of sepsis?" - to this day remains open.

Materials and methods

A retrospective, observational, cohort study of the dynamics of inflammatory response and hemostasis in patients with sepsis was performed. 83 patients were included in the study. The diagnosis of sepsis is based on the presence of a foci of infection and SOFA more than 4, confirmed by isolates of pathogens from the blood.

On the outcome of the disease, two groups of patients were formed: 1 - survivors (n = 41), 2 -dead (n- = 42). Laboratory data of patients from both groups were retrospectively studied: 1) at the time of diagnosis of sepsis and 2) the 5th day of the disease. The tests included the determination of blood leukocytes, young forms of neutrophils, lymphocytes, total plasma protein and albumin, plasma creatinine, C-reactive protein, procalcitonin (PCT), platelets, fibrinogen concentration, activity of antithrombin, D-dimer level. In dead patients, pathohistological studies were performed to identify microthrombosis of the vascular bed.

Results

At the stage of sepsis diagnosis in the groups of statistically significant differences by the studied parameters was not revealed. An exception was albumin and PCT, the values of which could be taken to predict the likelihood of an adverse outcome. Albumin (Odds ratio - 2.6 [1.0595 - 6.5038], P = 0.0371); PCT (Odds ratio - 9.2500 [2.7763 - 30.8184], P = 0.0003). However, the ROC analysis data do not allow us to substantiate their recommendation for the prognosis of the outcome of the disease. Albumin Cut-off ≤26 g/l, sensitivity/ specificity - 70%/53%; PCT - Cut-off > 16.2 ng/ml: sensitivity/specificity - 48%/90.

After 5 days (stage 2 of the study), the level of activity of antithrombin was significant for the prognosis of the outcome of sepsis (Odds ratio - 26,4 [8,02 - 8,6,86]); P <0.0001. ROC analysis of the level of activity of antithrombin as a predictor of lethal outcome revealed a high sensitivity (79%) and specificity (88%) for the value (Cut-off) ≤61%. Data of pathohistological studies of deceased patients revealed microcirculatory thrombosis of the vascular bed in 73.6%.

Conclusions

The obtained results show that at the stage of development of sepsis there is a high heterogeneity of indices of inflammatory reaction and hemostasis. The revealed regularity of the dynamics activity of antithrombin in patients with sepsis indicates one of the leading pathophysiological links in the multiple organ failure and allows us to recommend an antithrombin activity test for predicting the dynamics and probability of the outcome of the disease.

## P13 Open label prospective randomised control study of high cut point level of procalcitonin guided antibiotic therapeutic protocol in surgical critically ill patients

### Kaweesak Chittawatanarat, Narain Chotirosniramit, Kamtone Chandacham, Tidarat Jirapongcharoenlap, Rungnapa Peerakam, Mudjalin Areerug

#### Department of Surgery, Faculty of Medicine, Chiang Mai University, Chiang Mai, Thailand

##### **Correspondence:** Kaweesak Chittawatanarat (kchittaw@gmail.com)

Background

Post-operative fever is a common problem. In some situations, it is to distinguish infection from non-infection. Procalcitonin is claimed to be beneficial for identifying infection. However, the cut point value is still not concluded especially on post-operative critically ill patient. To show the benefit of efficacy of procalcitonin in post-operative fever in surgically critical ill patients. The cut point of less than 1 ng/ml is used for guiding physician to discontinue antibiotic. The mortality rate and reinfection rate were analyzed.

Materials and methods

An open label randomized control study was conducted. One hundred hospitalized patients in surgical intensive care unit, sub surgical intensive care unit and trauma intensive care unit of Maharaja Nakorn Chiang Mai Hospital from July 2016 to January 2017 were eligible in this study. The patients were divided into two groups, usual care group (UC) and procalcitonin-guided treatment group (PC).

Results

One hundred patients were included in the study. Baseline characteristics were not statistical significant different between group. Mean antibiotic duration and antibiotic free day were statistical significant different between group [PC vs. UC :8.5 days (IQR 5-17) vs. 14 days (IQR 8-28), (p=0.015); and 18 days (IQR 0-23) vs. 7.5 days (IQR 0-17), (p=0.023) respectively]. 90-day mortality and recurrent infection were not statistical significant different [90 days mortality (12/50) 24% vs. (11/50) 22%, Hazard ratio, HR, (95% confidence interval) 0.91 (0.41-2.03), p=0.818; hospital recurrent infection (14/50) 28% vs. (21/50) 42%, Hazard ratio, HR, (95% confidence interval) 0.62 (0.31-1.21), p=0.161.

Conclusions

The high cut point of procalcitonin level of 1 ng/ml or below 70% of initial level decrease antibiotic usage duration in post-operative surgical critically ill patients without differences on 90 days mortality and hospital re-infection.

Acknowledgements

Faculty of Medicine, Chiang Mai University.

## P14 Whether bacteremia is important in the treatment of sepsis?

### Julia Fuss¹, Anna Voloboyeva²

#### ¹Department of physical rehabilitation, D.Halycky Lviv National Medical University, Lviv, Ukraine; ²Department of intensive care Central Region Hospital in Pustomyty, Pustomyty, Ukraine

##### **Correspondence:** Julia Fuss (juliafuss78@gmail.com)

Background

Sepsis is a generalized infectious disease, has no propensity to self-destruct [1]. At present, the idea of the importance of bacteremia in sepsis is not systematized. There are data based on the frequency of bacterial isolation from the blood of patients with sepsis, which, depending on the severity of the patients, varies from 20 to 80% [2, 3].

Materials and methods

Diagnosis of 96 cases of sepsis with positive hemoculture in patients was carried out. The study included 58 men and 38 women aged 18 to 78 years. All patients were divided into groups depending on the underlying disease. Blood culture from all patients was carried out on nutrient media. The sensitivity of the isolated pathogens to antibacterial drugs was determined by diffusion into agar using discs.

Results

In just 3 years of observation, 155 cases of positive bacteremia, accompanied by an inflammatory reaction syndrome, were detected. In total, 89 strains of Gram-positive pathogens were isolated in monocultures, 27 Gram-negative pathogens in the culture, 19 mixed cultures of Gram-positive and Gram-negative microorganisms, 8 mixed cultures of Gram-positive microorganisms, 5 mixed cultures of Gram-negative microorganisms and 7 mixed cultures of bacteria and fungi. Gram-negative bacteria predominantly prevailed, constituted about 70% of all isolated strains, Gram-negative bacteria - about 29%, the frequency of fungal secretion was low. Among the representatives of Gram-positive bacteria, staphylococci were most often isolated, with *S.aureus* and coagulase-negative staphylococci standing out at approximately the same frequency. Among the representatives of Gram-negative bacteria, enterobacteria were most often isolated (17.7%), non-fermenting bacteria were isolated twice less often (8%). From the point of view the choice of empirical regime of antibacterial therapy (ABT), fundamental importance was causative agent of sepsis belongs to hospital or non-hospital microflora.

Effective ABT allows to reliably decrease the level of mortality in patients with sepsis. The effectiveness of sepsis treatment in different clinical groups of patients varies from 59-82%, according to different authors. On average, the efficiency of adequate ABT, according to our data, was 40%. The lethality of patients receiving adequate ABT, according to our data, is significantly lower than those receiving inadequate ABT (p <0.01). Timely correction of ABT, based on the sensitivity of the isolated pathogen, also significantly reduces lethality (p <0.01).

Conclusions

The initial regimens of ABT patients with sepsis, severe sepsis and septic shock do not correlate with the degree of severity of the systemic inflammatory reaction syndrome.

References

1. Bone R.C. Let us agree on terminology: definition of sepsis: Crit Care Med 1991; 19: 973-6.

2. Diab M, El-Damarawy M., Shemis M. Rapid identification of methicillin resistant staphylococcal bacteremia among intensive care units parients. Medscape J Med 2008; 10: 126.

3. Loffler C.A., Mac Dougall C. Update on prevalence and treatment of methicillin resistance *Staphylococcus aureus* infections. Expert Rev Anti Infect Ther 2007;5:961-81.

## P15 Treatment of purulent wounds with VAC-therapy

### Anna Voloboyeva¹, Julia Fuss²

#### ¹Department of intensive care Central Region Hospital in Pustomyty, Pustomyty, Ukraine; ²Department of physical rehabilitation, D.Halycky Lviv National Medical University, Lviv, Ukraine.

##### **Correspondence:** Julia Fuss (juliafuss78@gmail.com)

Background

In present the problem of treatment purulent-inflammatory diseases and is currently relevant to clinical surgery [1]. Despite significant advances, associated with the expansion deepening of knowledge about etiology, pathogenesis, clinical manifestations of surgical infection based on the modern advances in immunology, microbiology, biochemistry, reduction in number of patients and the severity of purulent surgical diseases is not observed [2]. Proper use of vacuum system can significantly reduce the wound regeneration and the cost of treatment for this category of patients. Vacuum therapy improves the course of all stages of the wound process [3,4].

Materials and methods

The results of treatment of 54 patients with severe surgical soft tissue infection at the age from 18 to 75 years who were treated at the department of purulent surgery were presented. In the treatment of 25 patients, system that created negative pressure was used in the wound in a constant or alternate regimen. The use of vacuum system and therapy with negative pressure was carried out after radical necrectomy. The control group consisted of 29 patients, in which the treatment was not included vacuum therapy. Keeping wounds in the control series was carried out in a semi-open manner using frequent dressings, local application of traditional antiseptics and ointment antibacterial compositions.

Results

In basic group in 19 patients from 25 in wounds identified a number of pathogens and opportunistic microorganisms. The analysis of the results of cytological control in the comparison groups allowed to establish that in the first 3-6 days after the operation, in all types of post-operative wound management, the necrotic type of the cytogram was dominated. The decrease in the level of bacterial insemination of the wound tissues below the critical (103 CFU/g) during NPWT therapy was achieved on average by the 3rd day against 7 days in traditional methods of local wound healing (P <0.05). In all patients, where was used vacuum system, pronounced positive effect of treatment was mostly noted, what optimized the timing of cleansing and healing of wounds. In the control group until the 15th day of observation, complete closure of the defect was noted only in 3 patients (16.7%).

Conclusions

The usage of wound healing technology by negative pressure promotes mechanical elimination from purulent foci of a large number of microbial bodies and products of tissue decay, which slows healing of the wounds, reduces interstitial edema of tissues, improves their lymph and blood circulation.

References

1. Anagnostakos, K. Bacteria identification on NPWT foams: clinical relevance or contamination / K. Anagnostakos, P. Mosser // Wound Care. – 2014. – Vol. 23. – P. 191–194.

2. Orgill, D. P. Negative pressure wound therapy: past, present and future / D. P. Orgill, L. R. Bayer // Int. Wound J. –2013. – Vol. 10, Suppl 1. – P. 15–9.

3. Schintler, M. V. Negative pressure therapy: theory and practice / M. V. Schintler // Diabetes Metab. Res. Rev. –2012. – Vol. 28, Suppl. 1. – P. 72–77.

4. Vacuum-assisted c losure : a new method f or wound control and treatment: animal studies and basic foundation / M. Morykwas [et al.] // Ann. Plastic Surg. –1997. – Vol. 38 (6). – P. 553–562.

## P16 Dynamic arterial elastance to predict mean arterial pressure after decreasing norepinephrine dosage in septic shock

### Nutchanok Niyatiwatchanchai^1^, Chalerm Liwsrisakun^1^, Kaweesak Chittawatanarat^2^, Theerakorn Theerakittikul^1^, Atikun Limsukon^1^, Pattraporn Tajarernmuang^1^, Chaicharn Pothirat^1^

#### ^1^Division of Pulmonary, Critical care and Allergy, Department of Internal Medicine, Faculty of Medicine, Chiang Mai University, Chiang Mai, Thailand; ^2^Department of Internal Surgery, Faculty of Medicine, Chiang Mai University, Chiang Mai, Thailand

##### **Correspondence:** Nutchanok Niyatiwatchanchai (n19_net@hotmail.com)

Background

Norepinephrine(NE) is recommended as the first-choice vasopressor for management of septic shock. There is no standard protocol or hemodynamic parameter for guiding NE tapering. Dynamic arterial elastance (Eadyn), defined as the ratio of pulse pressure variation (PPV) to stroke volume variation (SVV), has recently been proposed as a dynamic indicator of arterial tone.

Materials and methods

A prospective study was conducted in medical intensive care unit, Chiang Mai University hospital. Twenty-one mechanical ventilated patients with septic shock who were planned to decrease the NE dosage were enrolled. Hemodynamic parameters were obtained from FloTrac® monitoring system. Responders were defined as a 15% decrease in mean arterial pressure (MAP) during decreasing NE dosage period.

Results

Nineteen percent of patients (n=4) were responders. All baseline hemodynamic and arterial tone parameters were similar between responders and non-responders. Decreasing NE dosage induced significant variations in HR, SBP, DBP and MAP. Baseline Eadyn failed to predict lowering of MAP during decreasing NE dosage period (AUC = 0.69, 95%CI = 0.42–0.96).

Conclusions

Eadyn, obtained from FloTrac® monitoring system, was unable to predict decrement in MAP during decreasing NE dosage period at the time recovery from septic shock.

## P17 Prognostic value of Pentraxin-3, procalcitonin and lactate in patients with sepsis and septic shock in emergency departments

### Ju-Hyun Song^1^, Hyeri Seok^2^, Won Suk Choi^2^, Sungwoo Moon^1^, Dae Won Park^2^

#### ^1^Department of Emergency Medicine, Korea University Ansan Hospital, Ansan, Republic of Korea; ^2^Division of Infectious Diseases, Korea University Ansan Hospital, Ansan, Republic of Korea

##### **Correspondence:** Dae Won Park (pugae1@hanmail.net)

Background

Pentraxin-3 (PTX3) is an acute phase protein involved in inflammatory and infectious processes. The purpose of this study was to confirm the prognostic value of PTX3 compared to procalcitonin (PCT) and lactate in patient with sepsis and septic shock.

Materials and methods

A prospective, observational study was performed from December 2017 to May 2018 in a tertiary care hospital. Serum levels of PTX-3, PCT, lactate, and interleukin-6 (IL-6) were measured on admission and around outcome developed day.

Results

The study included 91 patients, 47 (51.6%) of whom were male, 45 (49.5%) of whom developed septic shock, and 35 (38.5%) of whom died in the stay of hospital. The level of PTX3, PCT, lactate and IL-6 at admission were significantly increased in septic shock patients compared to sepsis patients (45.4 ± 58.1 vs 100.7 ± 75.7 ng/ml, p<0.001; 7.1 ± 21.4 vs 18.7 ± 29.4 ng/ml, p=0.040; 2.5 ± 2.6 vs 6.2 ± 3.5 mmol/L, p<0.001; 1000.3 ± 2435.1 vs 2079.7 ± 46581.1 pg/ml, p=0.007, respectively). The AUROC of PTX3, PCT, lactate and IL-6 to predict in hospital mortality were 0.675 (95% confidential interval [CI] 0.54-0.81, p=0.019), 0.594 (95% CI 0.44-0.75, p=0.216), 0.648 (95% CI 0.51-0.79, p=0.047), and 0.723 (95% CI 0.59-0.86, p=0.003), respectively. Serial changes of PTX3 value in the death was significantly increased (85.7 ± 71.6 vs 229.3 ± 260.7 ng/ml, p=0.003).

Conclusions

PTX-3 reveals prognostic value for in hospital mortality compared to PCT and lactate. Serial increase of PCT3 might be poor prognostic marker.

## P18 The hemodynamic effects of oXiris hemofilter in septic shock patients requiring renal support: a case series

### Nuttha Lumlertgul^1,2^, Nattachai Srisawat^1,2,3^

#### ^1^Division of Nephrology, Department of Medicine, Faculty of Medicine, Chulalongkorn University, Bangkok, Thailand; ^2^Excellence Center of Critical Care Nephrology, King Chulalongkorn Memorial Hospital, Thai Red Cross Society, Bangkok, Thailand; ^3^The Center for Critical Care Nephrology, CRISMA, Department of Critical Care Medicine, University of Pittsburgh School of Medicine, Pittsburgh, Pennsylvania, USA

##### **Correspondence:** Nuttha Lumlertgul (nlumlertgul@gmail.com)

Background

The excessive release of pro-inflammatory and anti-inflammatory cytokines enables the latter to act as mediators for hemodynamic alterations, metabolic acidosis, and multi-organ failure in severe sepsis. Recently, the oXiris® hemofilter that comprises an AN69 core membrane, polyethyleneimine, and which is grafted with heparin, has been introduced as a novel hemofilter membrane to mitigate inflammatory response during sepsis-associated acute kidney injury (AKI) that requires renal replacement therapy (RRT).

Materials and methods

In the present case series, we retrospectively evaluated the impact of the oXiris hemofilter on hemodynamics and clinically relevant outcome parameters in critically ill patients with septic shock who require continuous RRT and those with at least two organ dysfunctions.

Results

Thirty-five patients were enrolled. There was a nonsignificant trend for improvement of hemodynamic status as shown by increased mean arterial pressure (MAP), decreased norepinephrine dose, inotropic score, and vasopressor dependency index. Blood lactate and base excess also showed significant improvement. oXiris treatment was safe with no device-related adverse events.

Conclusions

Using oXiris hemofilter was feasible, well-tolerated, and should encourage the conduction of randomized controlled trials to evaluate the potential benefits of this therapeutic option.

Acknowledgements

The authors would like to thank all fellows and staff at the Division of Nephrology, Chulalongkorn University and all intensivists who support this research. The authors would also like to thank Miss Dollapas Punpanich for statistical analysis.

## P19 Very high mortality in septic patients with unidentified pathogens and lower respiratory tract infections

### Anders Martinsen^1^, Dag Kvale^2^, Aleksander Rygh Holten^2,3^

#### ^1^Department of Acute Medicine, Oslo University Hospital, Oslo, Norway; ^2^Department of Infectious Diseases, Oslo University Hospital, Oslo, Norway; ^3^Department of Microbiology, Oslo University Hospital, Oslo, Norway

##### **Correspondence:** Aleksander Rygh Holten (aleksander.holten@gmail.com)

Background

Sepsis with unidentified pathogen is common and makes targeted therapy challenging. It is therefore likely that these patients receive less effective therapy than other patients with sepsis.

Materials and methods

All patients with suspected sepsis, taken care of by the medical emergency team in the Emergency department of a Norwegian secondary and tertiary care hospital, were prospectively included in this study. The patients were re-evaluated for sepsis after the hospitalization, sepsis diagnosed if the patient fulfilled both of the following criteria: I) Probable infection II) New or deteriorated organ dysfunction corresponding to a Sequential Organ Failure Assessment (SOFA) Score ≥2 in organ systems not primarily infected. Respiratory distress in relation to respiratory tract infection was for instance not included in the SOFA-calculation.

Results

A total of 487 patients were included from May 2017 to May 2018. Three patients were excluded due to incomplete registrations. 201 (42%) were assessed to have had true sepsis. A pathogen probably responsible for the septic episode was identified in 146 (73%). The overall 30 days mortality for sepsis was 17 %; 12% in patients with an identified pathogen opposed to 31 % for patients without an identified pathogen (p=0.001, see Table 1 below). High rates of lower respiratory tract infection were found among sepsis patients without identified pathogen, compared to patients with identified microbe (56% vs 29%, p<0.001). Moreover, septic patients with focus in the lower respiratory tract without identified pathogen had a significantly higher mortality rate (52% vs 21%, respectively; p=0.007).

Conclusion

Unidentified pathogen is a common feature in patients with lower respiratory tract infections and in our cohort also associated with very high mortality. Efforts should be made to improve early identification of pathogens in such patients.

**Table 1 (abstract P19).** See text for description

	Sepsis with identified pathogen	Sepsis WITHOUT identified pathogen	p-value
Patients	146	55	
Median age (first - third quartile)	73 (58 – 82)	79 (69 – 85)	0.027
Dead within 30 days (%)	17 (12 %)	17 (31 %)	0.001
Lower respiratory tract sepsis (%)	42 (29 %)	31 (56 %)	<0.001
Dead within 30 days with lower respiratory tract sepsis (%)	9 (21 %)	16 (52 %)	0.007
Urinary tract sepsis (%)	52 (36 %)	5 (9 %)	<0.001
Positive blood culture (%)	79 (54 %)	0 (0 %)	<0.001

## P20 Clinical characteristics and outcomes of patients with Leptospirosis Infection admitted to the Medical Intensive Care Unit: results from the 10-years retrospectively review from the university hospital from the South of Thailand

### Atta Ajjimarungsi, Rungsun Bhurayanontachai

#### Division of Critical Care Medicine, Department of Internal Medicine, Faculty of Medicine, Prince of Songkla University, Hat Yai, Songkhla, Thailand

##### **Correspondence:** Rungsun Bhurayanontachai (rungsun2346@gmail.com)

Background

Leptospirosis is the zoonotic infection, which is commonly found in all parts of Thailand. The severe form of this infection, as known as Weil’s syndrome, causes multiple organs dysfunction, such as liver failure, renal failure and respiratory failure. The severe Leptospirosis may leaded to morbidity and mortality after intensive care unit admission. We, hereby, presented the clinical characteristic and outcomes of severe Leptospirosis patients, who were admitted to the medical intensive care unit (MICU) in the university hospital from the South of Thailand.

Materials and Methods

The retrospectively chart review of the patients who was admitted to MICU with leptospirosis was done. Leptospirosis infection was confirmed by the positive microcapsule agglutination test for leptospira antibody from either the first or the second titer. Patients were then dichotomized into ICU-survivor and non-ICU-survivor group. All clinical characteristics included demographic data, clinical presentation and laboratory tests were recorded and compared by either Chi square test or independent t-test as appropriated. P-value < 0.05 was defined as statistically significant.

Results

Of 27 confirmation cases of severe leptospirosis, 77.8% was male with the mean age of 44.6+/-17.4 years old. 55.6% had history of water submersion. The median of presenting symptoms was 3(1,8) days. Fever (100%), myalgia (63%), vomiting (33.3%) and dyspnea (33.3%) were among the common clinical presentation. Mean creatinine level, mean bilirubin level and mean acute lung injury index (ALI) were 4.31+/-2.94 mg/dl, 5.51+/-4.92 mg/dl and 264.3+/-132.6, respectively. In addition, 81.5%, 77.8% and 25.9% of cases required inotropes, mechanical ventilator and dialysis during ICU admission, respectively. The overall ICU mortality was 14.8% (4/27). Female sex, acidemia, more severe lung injury and coma were significantly found in non-survivor case. Other parameters were comparable between groups. The comparison of survivor and non-survivor was shown in Table 1.

Conclusions

Leptospirosis is the lethal tropical infectious disease, which shown significant mortality in severe cases. Hemodynamic instability, acute kidney injury, acute liver injury and acute respiratory failure were among the common presentation required ICU admission. Female, acidemia, lung injury and coma were related to ICU mortality in this cohort.

## P21 Restricted Fluid Resuscitation in Sepsis associated Hypotension (REFRESH); a prospective, multicentre, clinical feasibility trial

### Stephen PJ Macdonald^1,2,3^, Gerben Keijzers^4,5,6^, David M Taylor^7^, Frances Kinnear^8^, Glenn Arendts^1,2,9^, Daniel M Fatovich^1,2,3^, Rinaldo Bellomo^10^, David McCutcheon^1,2,3,11^, John F Fraser^12^, Juan-Carlos Ascencio-Lane^13^, Sally Burrows^2^, Amanda Harley^4^, Matthew Anstey^14^, Ashes Mukherjee^11^

#### ^1^Centre for Clinical Research in Emergency Medicine, Perkins Institute of Medical Research, Perth, Australia; ^2^Medical School, University of Western Australia, Perth, Australia; ^3^Emergency Department, Royal Perth Hospital, Perth, Australia; ^4^Emergency Department, Gold Coast University Hospital, Gold Coast, Australia; ^5^School of Medicine, Bond University, Gold Coast, Australia; ^6^School of Medical Sciences, Griffith University, Gold Coast, Australia; ^7^Emergency Department, Austin Hospital. Melbourne, Australia; ^8^Emergency Department, The Prince Charles Hospital, Brisbane, Australia; ^9^Emergency Department, Fiona Stanley Hospital, Perth, Australia; ^10^Department of Intensive Care, Austin Hospital, Melbourne, Australia; ^11^Emergency Department, Armadale-Kelmscott Memorial Hospital, Perth, Australia; ^12^Critical Care Research Group, The Prince Charles Hospital, Brisbane, Australia; ^13^Emergency Department, Royal Hobart Hospital, Hobart, Australia; ^14^Department of Intensive Care, Sir Charles Gairdner Hospital, Perth, Australia

##### **Correspondence:** Stephen PJ Macdonald (stephen.macdonald@uwa.edu.au)

Background

Surviving Sepsis Campaign guidelines recommend resuscitation with at least 30ml/kg intravenous (IV) fluid in patients with sepsis and hypoperfusion [1]. Trials in developing countries have found higher mortality with larger resuscitation fluid volumes [2, 3]. A clinical trial of a smaller volume of fluid with earlier commencement of vasopressors is warranted, but it is unknown if this intervention is feasible and clinically acceptable in industrialised countries.

Materials and methods

The REstricted Fluid REsuscitation in Sepsis-associated Hypotension (REFRESH) trial (ACTRN126160000006448), a prospective, randomised, open-label, clinical feasibility trial was conducted in the emergency department (ED) of eight Australian hospitals between October 2016 and March 2018. The protocol has previously been published [4]. Inclusion criteria were suspected infection and hypotension (systolic blood pressure <100mmHg despite at least 1000ml IV fluid). Details of the trial intervention are summarised in Fig. 1. The primary outcome was total fluid administered within the first six hours from presentation. A range of process-of-care, feasibility and clinical outcomes were assessed including vasopressor requirement, protocol adherence, organ failure, and mortality at 90 days post randomisation.

Results

There were 99 participants (49 standard and 50 restricted volume) in the intention-to-treat analysis (Fig. 2). Baseline participant characteristics are shown in Table 1. Fluid volume (total for 24 hours from randomisation) and vasopressor use are summarised in Table 2. A significantly lower volume was administered over the first six hours in the restricted volume group (median 30ml/kg vs 43ml/kg, p<0.001) along with earlier commencement of vasopressors (median 1h vs 2h post randomisation, p=0.001). There was no significant difference in the rate of ICU admission, ventilation and acute kidney injury (including dialysis) between the groups. At 90 day follow up 3 of 47 (6%) in the standard arm and 4 of 48 (8%) in the restricted arm had died (P=1.0).

Conclusions

A restricted fluid and early vasopressor regimen among ED patients with suspected sepsis and hypotension led to a significant reduction in fluid volume given over the first 6 hours and at 24 hours. Median dose and duration of vasopressors were lower in the restricted volume group, but this was not statistically significant. There were no differences in clinical outcomes, however the study was not designed to detect these. The results demonstrate the feasibility and clinical acceptability of the trial intervention in the Australian ED setting. A large randomised trial to assess the effect on clinical outcomes should be pursued.

Acknowledgements

On behalf of the REFRESH Investigators. The trial was part-funded by the Emergency Medicine Foundation (Queensland).

References

1. Rhodes A, Evans LE, Alhazzani W, Levy MM, Antonelli M, Ferrer R, Kumar A, Sevransky JE, Sprung CL, Nunnally ME, et al. Surviving Sepsis Campaign: International Guidelines for Management of Sepsis and Septic Shock: 2016. Intensive Care Med 2017, 43:304-377

2. Maitland K, Kiguli S, Opoka RO, Engoru C, Olupot-Olupot P, Akech SO, Nyeko R, Mtove G. Reyburn H, Lang T, et al, on behalf of the FEAST trial group. Mortality after fluid olus in Africal children with severe infection. N Engl J Med 2011, 364:2483-2495

3. Andrews B, Semler MW, Muchemwa L, Kelly P, Lakhi S, Heimburger DC, Mabula C, Bwalya M, Bernard GR. Effect of an Early Resuscitation Protocol on In-hospital Mortality Among Adults With Sepsis and Hypotension. JAMA 2017, 318:1233-1240

4. Macdonald SPJ, Taylor DM, Keijzers G, Arendts G, Fatovich DM, Kinnear FB, Brown SGA, Bellomo R, Burrows S, Fraser JF, et al. REstricted Fluid REsuscitation in Sepsis-associated Hypotension (REFRESH): study protocol for a pilot randomised controlled trial. Trials 2017,18:399

**Table 1 (abstract P21).** Participant characteristics at baseline. Values are median (interquartile range) unless otherwise stated. SBP – systolic blood pressure; APACHE – Acute Physiology and Chronic Health Evaluation; SOFA – Sequential Organ Failure Assessment

	Standard volume N=49	Restricted volume N=50
Age	66 (45, 76)	66 (52, 78)
Male Sex n (%)	30 (61)	31 (62)
Weight (kg)	72 (64, 90)	80 (66, 88)
SBP at randomisation	89 (84, 92)	88 (79, 91)
Lactate	1.8 (1.2, 2.6)	1.7 (1.1, 3.5)
Charlson score	2 (0, 4)	2 (1, 4)
APACHE II score	14 (10, 18)	15 (10, 20)
SOFA score	5 (4, 7)	5 (3, 10)
Non-CVS SOFA score	3 (2, 4)	3 (1, 6)
Creatinine (μmol/L)	130 (80, 170)	106 (75, 160)
Acute Kidney Injury N (%)	30 (60)	26 (52)
Sepsis Source N (%)		
Respiratory	20 (41)	14 (28)
Urinary	9 (18)	16 (32)
Skin/soft tissue	6 (12)	6 (12)
Bloodstream	7 (14)	3 (6)
Abdominal/pelvis	2 (5)	5 (10)
Other/unidentified	5 (10)	6 (12)

**Table 2 (abstract P21).** Fluid and vasopressor use. Values are median (interquartile range) unless otherwise stated. T0 -randomisation; T6 – 6 hours post-randomisation; T24 – 24h post-randomisation

	Standard volume N=49	Restricted volume N=50	P value
Fluid Volume			
Pre-randomisation	1250 (1000, 2000)	1450 (1000, 1500)	0.69
T0-T6 (ml)	1655 (1017, 2500)	938 (592, 1458)	<0.001
T0-T6/kg (ml)	23 (15, 33)	12 (7, 20)	<0.001
Total prerandomisation-T6	3000 (2550, 3900)	2387 (1860, 2750)	<0.001
Total to T6/kg (ml)	43 (35, 49)	30 (23, 39)	<0.001
T6-T24 (ml)	1000 (428, 1743)	1134 (500, 2000)	0.73
Total prerand-T24 (ml)	4250 (3500, 5207)	3543 (2812, 4410)	0.005
Total to T24/kg (ml)	61 (46, 79)	40 (33, 64)	0.005
Vasopressor use N (%)	26 (53)	39 (78)	
Vasopressor in ED N (%)	23 (47)	36 (72)	0.011
Vasopressor at 24h N (%)	19 (39)	24 (48)	0.35
Time to vasopressor commencement from T0 (h)	2 (1, 3)	1 (1, 1)	0.001
*Type of vasopressor*			
Noradrenaline N (%)	23 (47)	30 (60)	0.33
Metaraminol only N (%)	3 (2)	9 (18)	
Central venous access N (%)	20 (41)	26 (52)	0.42
Volume prior to vasopressor commencement (ml)	2000 (2000, 2777)	1400 (1000, 1700)	<0.001
Duration of vasopressor (h)	33 (15, 50)	21 (9, 42)	0.13
Peak vasopressor dose (noradrenaline equivalent) (mcg/kg/min)	0.18 (0.1, 0.43)	0.11 (0.08, 0.22)	0.14
Mean arterial pressure T0-T6 (mmHg)	72±6	73±6	0.31

**Fig. 1 (abstract P21).**
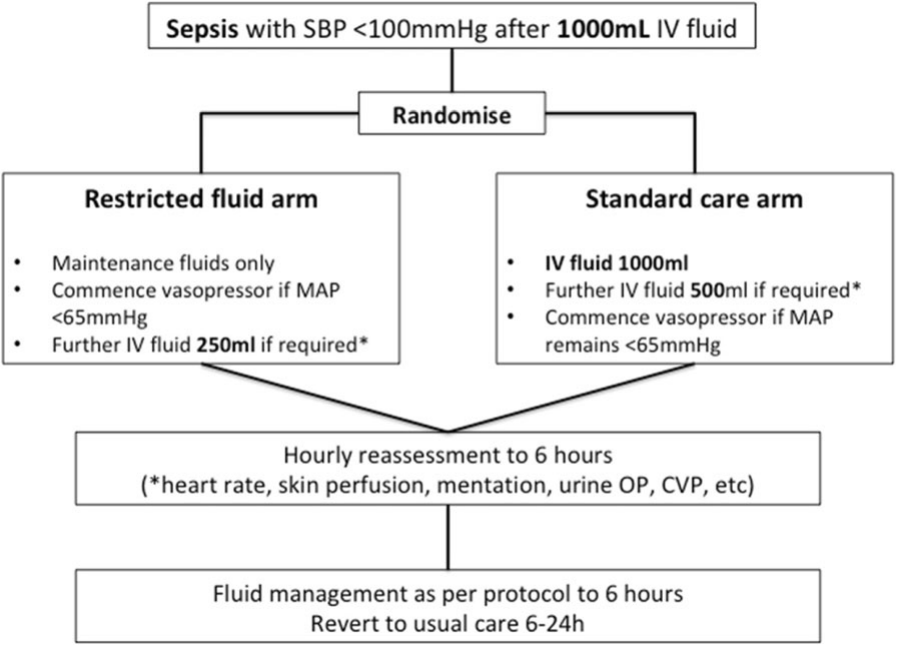
Summary of trial interventions

**Fig. 2 (abstract P21).**
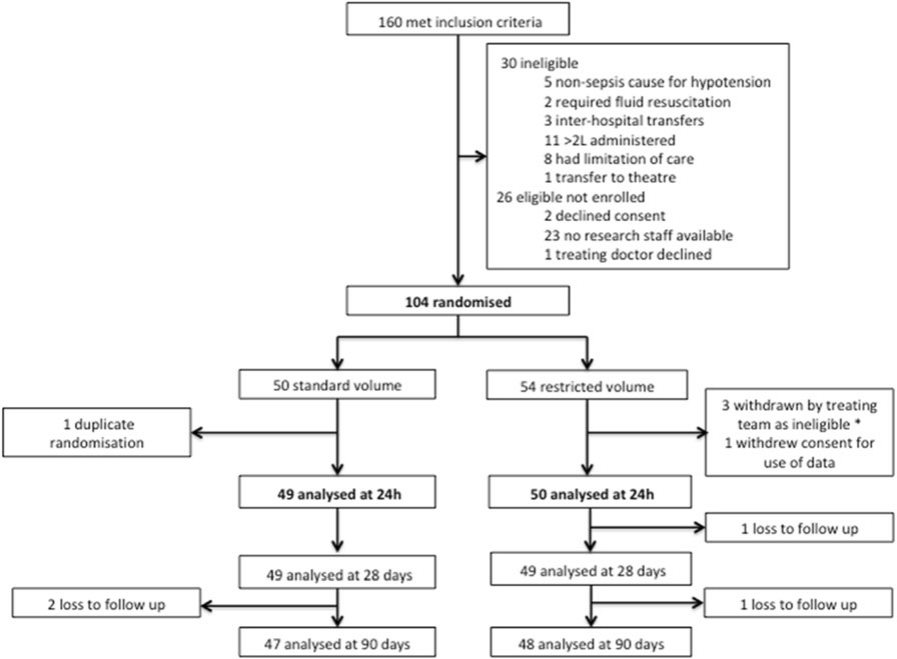
Consort flowchart of participants through the trial. *Met exclusion criteria on basis of limitation-of-care order

## P22 Practice of hemosorption with CytoSorb adsorber for sepsis in cancer patients

### Natalia Dmytrievna Ushakova (ndu2000@rambler.ru)

#### Department of Anesthesiology and Reanimation, Rostov Scientific Research Oncology Institute of the Ministry of Health of Russia, Rostov-on-Don, Russia

Background

Numerous studies in the animal experiment, as well as preliminary data on treatment of septic patients have demonstrated that a decrease in cytokine levels in blood can effectively reduce the inflammatory response in sepsis and possibly improve clinical outcome. The aim of the study was to evaluate the effect of hemosorption using the CytoSorb column on the course of generalized inflammation in cancer patients.

Materials and methods

11 patients with sepsis were examined. All patients gave voluntary informed consent for the research and publication of the results. Microbiological identification was obtained in 7 cases. The severity of the initial condition on the APACHE-II scale was 24.2 ± 3.3 points, SOFA − 9.2 ± 2.3 points. Organ abnormalities requiring organ-replacement treatment were not recorded. Blood PCT was > 2 ng/ml, EAA did not exceed 0.5. In all cases, hemosorption using the CytoSorb column was included in the treatment package. Indications for its use was IL-6 blood concentration of more than 800 pg/ml, as an indicator of the severity of hypercytokinemia, which, as previously identified, correlated with a high risk of of generalized inflammation progression and MOF. Hemosorption was performed by the Multifiltrate machine (Fresenius Medical Care, Germany) for a period of 12 - 24 hours. Vascular access was v.subclavia/v.femoralis. Perfusion rate was 150 ml/min. Heparinization was performed using unfractionated heparin. PCT, CRP, IL-6, IL-10 in blood serum were studied. Measurements were taken before hemosorption, 1 and 6 hours after its completion. The statistical processing of the results was performed using "Statistica 6.0" program with the calculation of the Student's reliability criterion (t). The difference was considered significant for p<0.05.

Results

After hemosorption using the CytoSorb column, in 8 out of 11 cases the regress of generalized inflammation activity was noted. PCT reduction was recorded from 27.3 ± 6.7 ng/ml to 4.3 ± 1.1 ng/ml; CRP - from 467.2 ± 98.6 mg/ml to 105.1 ± 54.3 mg/ml; IL-6 - from 1461.3 ± 102.3 pg/ml to 346.5 ± 91.1 pg/ml; IL-10 - from 46,1 ± 4,3 pg/ml to 2,1 ± 0,4 pg/ml; blood lactate - from 5.9 ± 1.7 mg/ml to 1.9 ± 1.2 mg/ml (p<0.05). The SOFA index in these patients decreased by 4.1 ± 1.2 points (p<0.05). In 3 patients inflammatory reaction activity and organ disorders progression was diagnosed, which required the use of other extracorporeal detoxification methods.

Conclusions

Extracorporeal removal of excessively produced inflammatory mediators is an effective and safe auxiliary "tool" in the treatment of sepsis in cancer patients. However, in order to determine the criteria for indications, timing and tactics of combined use with other methods of extracorporeal detoxification, further in-depth studies are needed.

## P23 Simple Clinical Score: Predicts mortality in sepsis to early warning score of the emergency room

### Manaporn Chatchumni^1^, Sangrawee Maneesri^1^, Prapapun Singto^2^, Karn Yongsiriwit^3^

#### ^1^School of Nursing, Rangsit University, Pathum Thani, Thailand; ^2^Department of Medical, Singburi Hospital, Sing Buri, Thailand; ^3^College of Digital Innovation and Information Technology, Rangsit University, Pathum Thani, Thailand

##### **Correspondence:** Manaporn Chatchumni (manaporn@rsu.ac.th)

Background

Raising mortality rate of septic patients has been brought to the emergency room and transfer admission to a medical department. Acute Physiology and Chronic Health Evaluation (APACHE), Simplified Acute Physiology Score (SAPS II) and Simple Clinical Score (SCS) have been used to assess both of acutely and critically ill in the intensive care units and an acute medical unit[1, 2,3]. A limited of tools has been used to assess the early warning score in the emergency room.

Materials and methods

A retrospective case was used designed to describe SCS[2,3] predictive abilities of mortality rate in the patient with sepsis and to compare variables of SCS predictive abilities of different parameters (a status without coma, intoxication or overdose, and aged ≥50 years, new stroke on presentation including age, systolic blood pressure (mmHg), pulse rate > systolic blood pressure, temperature, respiratory rate (per min), oxygen saturation, breathless on presentation, abnormal ECG, diabetes (type I or II), coma without intoxication or overdose, altered mental status, unable to stand unaided, or a nursing home resident, prior to current illness, spent some part of daytime in bed. Those lead to mortality. A retrospective analysis of the patient chart record data from all patients diagnosed such as sepsis, severe sepsis or septic shock admitted to the emergency room from December 2016 - November 2017. Local Hospital Research Ethics Committee approval was obtained under code: สห 0032.2052/80.

Results

Based on the dataset of 225 patients obtained from the emergency room, we discover that 133 (59.1%) patients are dead, 85 (37.8%) are discharged and 7 (3.1%) are transferred to another hospital as shown in Fig. 1. To describe SCS predictive abilities of mortality rate in the patient with sepsis. Firstly, we use the predictive SCS model to compute the level of SCS for all patients divided by their status after the admission. The results can be summarized in Table 1.

Therefore, the mortality rate for each level of SCS are computed and described in Table 2. The results show that the patients with high and very high level of SCS are dead at the percentage of 26 and 74, respectively.

The second objective is to compare variables of SCS predictive abilities of different parameters lead to mortality. Firstly, we separate our dataset to trained and test data. The trained data consists of 75% of the dataset (167 patient records) and the test data consist of 25% of the dataset (58 patient records). We create a logistic regression model from trained data using the patient’s status ‘dead’ as outcome, with all the variables as independent predictors. From the created model, Table 3 show the important variables for predicting deadly outcome. Oxygen saturation, prior to current illness, spent some part of daytime in bed, coma without intoxication or overdose, respiratory rate (per min) and abnormal ECG are the most important variable for predicting deadly outcome, respectively. We measure the performance of our model using the sensitivity and specificity analysis that are shown sensitivity of 86.7% and specificity of 92.9% for SCS predicts mortality.

We test our model by prediction and comparing with the train data. The result is plotted as the Receiver Operator Characteristic (ROC) curve in Fig. 2. We also calculate the Area Under the Curve (AUC) as AUC = 0.6954657 meaning that the prediction accuracy is quite poor which could be due to the limitation of our dataset.

Conclusions

SCS are not sufficiently accurate to predict mortality in patients with sepsis, which is quite poor quality. However, the outcomes of this analysis known the important variable identification tool for deteriorating patients to early warning sign for detection and prevent adverse events in the patient with sepsis.

References

1. Hamilton F, Arnold D, Baird A, Albur M, Whiting P: Early Warning Scores do not accurately predict mortality in sepsis: A meta-analysis and systematic review of the literature. Journal of Infection 2018,76(3):241-8.

2. Kellett J, Deane B: The Simple Clinical Score predicts mortality for 30 days after admission to an acute medical unit. Journal of the Association of Physicians. 2006,99(11):771-81.

3. Hamilton F, Arnold D, Baird A, Albur M, Whiting P: Early warning scores do not accurately predict mortality in sepsis: a meta-analysis and systematic review of the literature. Journal of Infection. 2018, 11.

**Fig. 1 (abstract P23).**
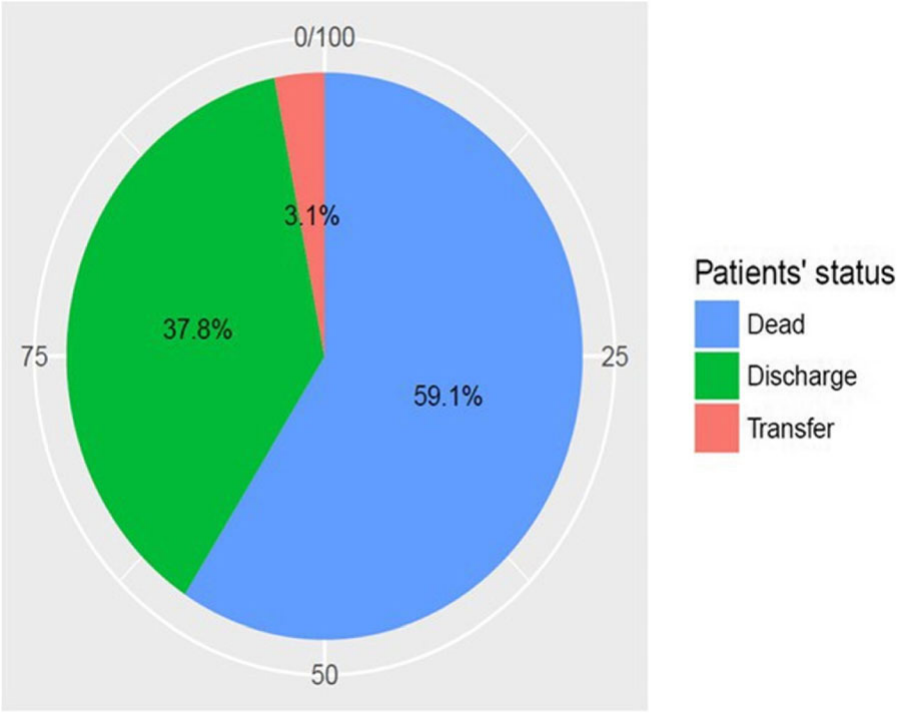
The status of SEPSIS patients after admitted to the emergency room

**Table 1 (abstract P23).** The level of SCS for all patients

Level of SCS	Dead	Discharge	Transfer
Very low	0	3	0
Low	0	7	0
Average	0	9	0
High	9	26	0
Very high	124	40	4

**Table 2 (abstract P23).** The mortality rate for each level of SCS

Level of SCS	Mortality rate
Very low	0.0
Low	0.0
Average	0.0
High	25.7
Very high	73.8

**Table 3 (abstract P23).** The variable importance for logistic regression model

Variable	Estimate	Std. Error	z value	Pr(>|z|)	Importance
Oxygen saturation					
≥95%	1.573306	0.4167	3.775611	0.00016	3.775611
≥90% and <95%					
<90%					
Prior to current illness, spent some part of daytime in bed	0.87259	0.29709	2.937088	0.00331	2.93709
Coma without intoxication or overdose	0.45074	0.16175	2.78673	0.00532	2.78673
Respiratory rate (per min)					
≤20	1.141778	0.44229	2.581525	0.00984	2.581525
>20 and ≤30					
>30					
Abnormal ECG	0.575612	0.32636	1.763741	0.07778	1.763741
Pulse rate>systolic blood pressure	0.374006	0.27959	1.337682	0.181	1.337682
Diabetes (type I or II)	0.511602	0.41016	1.24733	0.21228	1.24733
Altered mental status without coma, intoxication or overdose, and aged ≥50 years	0.281089	0.30121	0.933204	0.35071	0.933204
Breathless on presentation	-0.88704	0.98829	-0.89756	0.36942	0.897558
Systolic blood pressure (mmHg)					
>100	0.218143	0.24314	0.897179	0.36962	0.897179
>80 and ≤100					
≥70 and ≤80					
<70					
Age (years)					
<50 for men or <55 for women	-0.11581	0.21299	-0.54376	0.58661	0.543756
≥50 for men and ≥55 for women but ≤ 75 for either					
>75 for both men and women					
Temperature <35 °C or ≥39 °C	-0.07154	0.29291	-0.24423	0.80705	0.244228
Unable to stand unaided, or a nursing home resident	9.354837	811.949	0.011521	0.99081	0.011521
New stroke on presentation	5.68584	1066.55	0.005331	0.99575	0.005331

**Fig. 2 (abstract P23).**
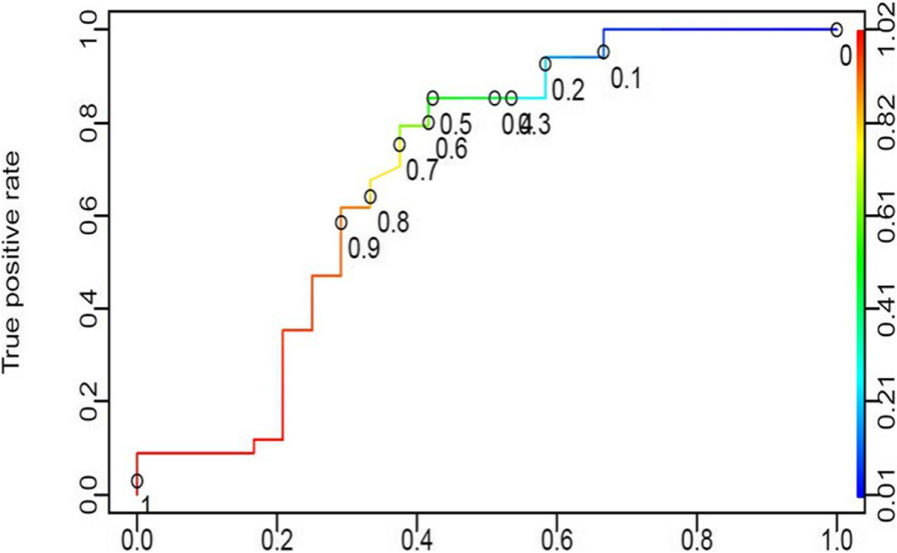
The ROC curve

## P24 The efficiency of the Holographic glasses (HoloLens) with Holographic technology for Tele-ICU consultation

### Ratapum Champunot^1^, Paisarn Muneesawang^2^, Jessada Methrujpanont^3^, Sirikasem Sirilak^4^

#### ^1^Phitsanulok Provincial Public Health Office, Phitsanulok, Thailand; ^2^Faculty of Engineering, Naresuan University, Muang Phitsanulok, Thailand; ^3^Buddhachinaraj Phitsanulok Hospital, Phitsanulok, Thailand; ^4^Faculty of Medicine, Naresuan University, Muang Phitsanulok, Thailand

##### **Correspondence:** Ratapum Champunot (mr.sepsis@gmail.com)

Background

In many countries, critically ill patients in the ICU will not match with the number of available intensivists. And, as with other specialties, many hospitals struggle due to a growing shortage of intensivists. Tele-ICU intensivists provide real-time services to multiple care centers regardless of their locations and are most helpful in reducing the time needed to consult an intensivist with populated areas lacking in critical care professionals. HoloLens opens up radically new ways for medical treatment and education. This study explores the use of the HoloLens by intensivists to provide advice to non-ICU physicians and medical personnel in the ICU.

Materials and methods

A Microsoft HoloLens with Hologram technology consulting application (Nu-med Consult application) that designed and developed to use for the consulting system was used in this study. This study operated in 4 ICUs (1 case-mix ICU, 1 Surgical ICU, 1 Cardiovascular Thoracic Surgical ICU and 1 Trauma ICU) in one tertiary care hospitals and one private hospital during 11 April – 11 May 2018. The main purpose of this study was to evaluate the efficiency of the HoloLens for the consulting system in the ICU.

Results

There are totally 17 patients that admitted in ICU were consulted through HoloLens in the period of this study. The topics of consultation were problems in ventilator settings (9 patients), change of symptoms and laboratory results notification (7 patients) and making a decision for ventilator support (1 patient). The questionnaire was used to evaluate the satisfaction of hologram technology in 3 aspects (the system, the utility and the development team) by 1 CVT physician, 1 intensivist and 14 ICU nurses who used HoloLens for the consulting system in the ICU. The mean (SD) of the satisfaction of hologram technology in 3 aspects were 4.37 (0.62), 4.50(0.63) and 3.87(0.50) respectively (score out of 5). When compared with the old-fashioned consultation through LINE application, HoloLens did not reach expectations from the user’s perspective.

Conclusions

The HoloLens introduces novel opportunities that highly skilled staff trained in critical care able to deliver timely, quality care service to patients admitted to ICU in the areas that lack healthcare professional. In addition, it can be used as another effective way of bedside training for non-ICU physicians and nurses in ICU with limited experience. However, there are some limitations of HoloLens that should be developed to be better for more convenient use in the future.

Acknowledgements

Piya Sirilak, MD (Director of Phitsanulok provincial public health office).

## P25 Diagnosis of sepsis at the point of care

### Riya Palchaudhuri^1,2^, Suzanne Crowe^1,2,3^, Steve McGloughlin^5^, Mary Garcia^1^, Clovis Palmer^1,2^, David Anderson^1,4*^

#### ^1^Burnet Institute, Melbourne, VIC, Australia; ^2^Monash University, Department of Medicine, Melbourne, Australia; ^3^The Alfred Hospital, Infectious Diseases Unit, Melbourne, Australia; ^4^The University of Melbourne, Department of Microbiology and Immunology, Melbourne, Australia; ^5^The Alfred Hospital, Intensive Care, Melbourne, Australia

##### **Correspondence:** David Anderson (David.anderson@burnet.edu.au)

Background

Rapid diagnosis is essential in the case of sepsis. Current methods for diagnosis of sepsis have insufficient specificity causing delay in clinical intervention. Neutrophil CD64 index, measured by Flow cytometry, shows great promise as a sensitive and specific marker of neonatal sepsis, but its use is severely constrained due to lack of access to high quality Flow cytometry. Our hypothesis was that use of immunoassays for simultaneous measurement of the total of two proteins, neutrophil CD64 and a neutrophil-specific protein in whole blood, which together yields the nCD64 index as measured by Flow Cytometry.

Methods

We are recruiting adult ICU patients (n=50) with clinically suspected sepsis (Alfred ICU) and age-matched controls from the ICU (n=50), and healthy individuals (n=50). Thus far 40 patients with clinically suspected sepsis, 18 ICU controls and 50 healthy volunteers have been recruited. Levels of a selected neutrophil specific marker, neutrophil elastase (NE), and neutrophil activation marker (CD64) are measured by commercial sandwich ELISA kits. Samples were also tested using Leuko64^TM^ assay, procalcitonin electrochemiluminescence immunoassay (PCT ECLIA) and further examined by flow cytometry with intracellular staining, and microscopy.

Results

Analysis of total CD64 and NE in whole blood demonstrated non-linear correlation in healthy controls, allowing us to define two separate methods for assigning assay cut-offs based on either (1) total CD64 (Fig. 1A), or (2) elevated CD64 relative to NE for samples with NE < 2.5 μg/ml (Fig. 1B), representing neutropenic patients. The combination of both these methods allowed detection of 33/34 sepsis patients versus 6/61 controls (Sensitivity 97%, Specificity 90%). Conversely, in sepsis patients, the Leuko64 kit (flow cytometry) was positive in 26/34 (76%) patients, suggesting that the expected upregulation of neutrophil-CD64 during sepsis includes intracellular CD64 that is detected by ELISA but not by the Leuko64 staining method. PCT ECLIA detected 24/30 sepsis patients (80%; 95% CI 62.3 to 90.9%).

Conclusion

Measurement of both total CD64, i.e. both surface and intracellular levels of CD64, and NE levels in whole blood appears to be a strong candidate biomarker for diagnosis of sepsis. Further studies in larger cohorts of patients and controls are planned, along with the development of a point-of-care version of the test using methods we have previously used for CD4 T-cells (Visitect® CD4, Omega Diagnostics).

**Fig. 1 (abstract P25).**
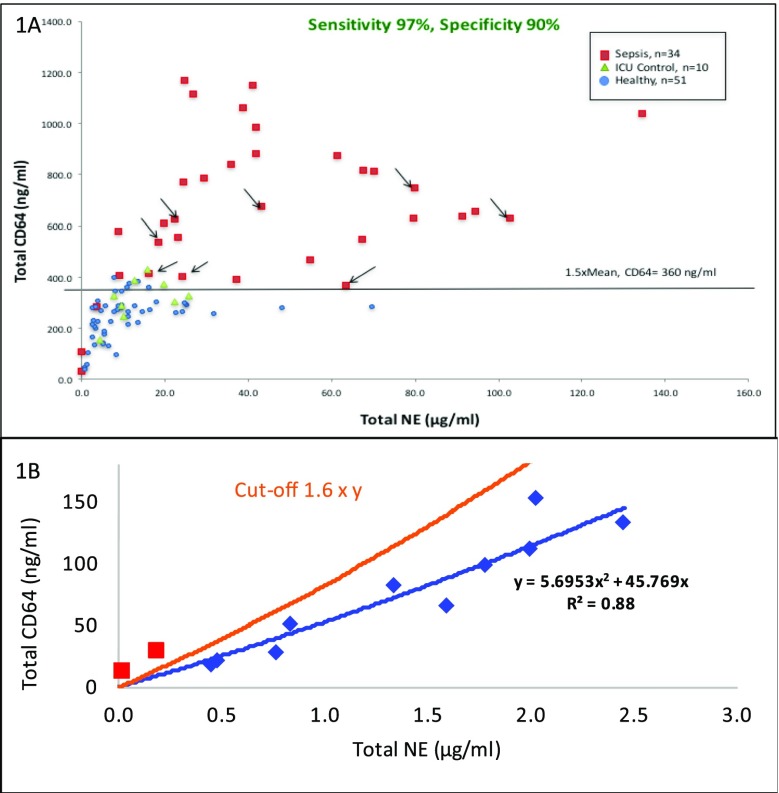
CD64 and NE ELISA for whole blood from patients with suspected sepsis (n=34, red squares) or ICU controls (n=9, green triangles) or healthy (n=51), blue circles). (A) Assigned cut-off for total CD64 (1.5 x Mean); 31/34 sepsis patients have levels of CD64 above these cut-offs. Arrows: These eight patients negative by Leuko64^TM^ flow cytometric assay were positive for Total CD64. (B) Expanded data for samples with levels of total NE <2.5 μg/ml, including the 2 neutropenic sepsis samples (red) that have elevated levels of CD64 compared to an assigned cut-off of 1.6 x the trendline for healthy controls.

## P26 Rapid identification of pathogens from whole blood clinical samples in critically septic patients by technology of Magnetic Resonance

### Cinzia Peronace, Angela Quirino, Aida Giancotti, Giorgio Settimo Barreca, Luisa Galati, Elena Colosimo, Valentina Tancrè, Giovanni Matera, Maria Carla Liberto, Alfredo Focà

#### Institute of Microbiology, Department of Health Sciences, “Magna Graecia” University, Catanzaro, Italy

##### **Correspondence:** C. Peronace (cinziaperonace@hotmail.it)

Background

Rapid and accurate profiling of infection-causing pathogens is crucial for critically septic patient management. During our previous investigations, we evaluated both the performance of some syndromic tests [1,2] and the most successful sepsis biomarkers [3]. Despite advances in molecular diagnostic techniques, blood culture analysis remains the gold standard for diagnosing sepsis. A rapid molecular non-cultural method was recently developed for identification of Candida and bacterial bloodstream infections from whole blood samples. The aim of this study was to evaluate the performance of an automated assay that utilize proprietary T2 Magnetic Resonance technology to enable faster and potentially accurate identification for pathogens that cause sepsis directly from whole blood clinical samples.

Materials and methods

Thirty-six whole blood samples, obtained from critical patients admitted to “Magna Graecia” Hospital of Catanzaro (Italy), were analyzed by innovative technology of Magnetic Resonance (T2 Biosystems, Inc., USA) and compared to a conventional laboratory method (Vitek2, bioMèrieux, France). T2MR combines the nuclear magnetic resonance and PCR molecular assay to directly detect six bacteria species (*Enterococcus faecium*, *S. aureus*, *A. baumannii, E. coli*, *P. aeruginosa* and *K. pneumoniae* ) and the five most common Candida species (*C. albicans*, *C. tropicalis, C. glabrata*, *C. parapsilosis*, *C. krusei*) directly from freshly obtained whole blood samples, in approximately 3.5-5 h. Specifically, T2 system lyses red blood cells, concentrates microbial cells and cellular debris by mechanical bead-beating, amplifies the DNA using a thermostable polymerase and target-specific primers. The amplified DNA is detected by hybridization to the superparamagnetic nanoparticles, which causes large changes in the sample’s T2MR signal. A synthetic heterologous DNA target used as an internal control, is processed with each clinical specimen, to monitor the integrity of the T2MR results. The limit of detection of T2MR is 1 colony-forming unit (CFU)/mL.

Results

Of the overall 36 whole blood samples tested for candida and bacteria panels, four specimens were not included in the analysis because the internal control was invalid, as well as the samples whose blood cultures were missing (seven specimens). Data obtained by T2MR was confirmed in 100% (11/11 cases) for T2Candida panel when compared to Vitek2 method as well as antigen mannan. Concerning T2Bacteria panel, the assay was able to identify 12/14 (86%) specimens when compared to conventional laboratory method respectively. Furthermore, in one case, the novel T2MR assay was capable to detect *A. baumannii* species after 3.5 h, while the positivity of the conventional blood culture was obtained only six days before.

Conclusions

The data of this study demonstrated that T2MR represents a highly promising molecular diagnostic method for the rapid identification of the most important pathogens causing-sepsis. The use of such technology may warrant a place in the diagnostic pathway of septic syndrome.

Acknowledgements

Authors wish to thank Diagnostic International Distribution (DID, Italy) for the kindly provided T2MR reagents.

References

1. C. Peronace, G. Matera, R. Puccio, V. Marano, G.S. Barreca, A.G. Lamberti, A. Giancotti, A.Quirino1, M.C. Liberto1, A. Foca Evaluation of a multiplex PCR assay for the rapid identification of bacteria and yeasts in positive blood cultures. ECCMID 2014

2. A. Focà, C. Peronace, A. Quirino, L. Galati, G.S. Barreca, L. Gallo, P. Morelli, G. Cortese, M.C. Reale, P. Settembre, I.

Addolorato,G. Matera, M.C. Liberto, P. Minchella Evaluation of an automated microscopy-based multiplex FISH assay for

bacteria and yeasts identification in positive blood cultures Intensive Care Medicine Experimental 2017, 5(Suppl 1):P41

3.Matera G, Quirino A, Peronace C, Settembre P, Marano V, Loria MT, Marascio N, Galati L, Barreca GS, Giancotti A, Amantea B, Liberto MC, Focà A. Soluble CD14 Subtype-A New Biomarker in Predicting the Outcome of Critically Ill Septic Patients. Am J Med Sci. 2017, 353(6):543-551

## P27 Intriguing behavior of cytokines and specific markers in sepsis and septic shock

### Cinzia Peronace, Giovanni Matera, Luisa Galati, Aida Giancotti, Angela Quirino, Concetta Zangari, Orietta Eramo, Maria Carla Liberto, Alfredo Focà

#### Institute of Microbiology, Department of Health Sciences, “Magna Graecia” University, Catanzaro, Italy

##### **Correspondence:** C. Peronace (cinziaperonace@hotmail.it)

Background

Our previous studies dealing with pathophysiology of sepsis stressed the main diagnostic role of procalcitonin (PCT) and the major prognostic role of soluble CD14-subtype (presepsin) [1,2]. The aim of the present investigation was the assessment of the potential prognostic role of the principal cytokines of Th1, Th2 and Treg cascades.

Materials and methods

In this observational study, we consecutively enrolled critical patients admitted to the Unit of Intensive Care (ICU) of the University Hospital of Catanzaro, Italy. For such patients admission SOFA scores were calculated. Based on 28 days survival subjects were stratified in survivors and nonsurvivors, while a group of healthy volunteers were also included as negative controls. All enrolled patients were subjected to blood cultures. Serum samples were tested for presepsin (PATHFAST Presepsin assay) and procalcitonin (VIDAS B.R.A.H.M.S PCT, bioMérieux, Italy). For all such patients, cytokines levels were evaluated by biochip immunoassay with high sensitivity panel (Randox,) at the time of hospital admission. Data were subjected to statistical analysis by ANOVA plus post-hoc PLSD Fisher’s test.

Results

Our data confirmed that presepsin is a valuable prognostic biomarker for the studied patients (p=0.0169). However, PCT did not behave as an affordable prognostic marker. The Treg cytokine, IL-10 seemed to exhibit a trend toward increased values in dead vs. alive patients. Th1 cytokines did not show any significant difference between dead and alive patients at admission time. The Th2 cytokine, IL-4 followed the expected behavior of a lack of any significance in such very early sepsis phase.

Conclusions

In conclusion lack of significance between different groups of cytokines evaluated, further support the value of presepsin as an excellent prognostic biomarker. Although a trend toward significancy was found in dead vs. alive patients for IL-10, reflecting the basal higher level of such immunosuppressive mediator even in early sepsis pathophysiology. An unexpected result was the lack of significant difference between the same groups regarding Th1 cytokines, which should be quite higher in such early sepsis stage, particularly in non-survivors. Both presepsin and cytokine prognostic role warrants to be assessed in larger cohorts of septic patients.

References

1.Cinzia Peronace, Giovanni Matera, Luisa Galati, Aida Giancotti, Giorgio Settimo Barreca, Angela Quirino, Maria Carla Liberto, Alfredo Focà

IL-35 and soluble CD14 subtype might be useful prognostic markers in critical septic patients Critical Care 2016, 20(Suppl 3):P24

2.Matera G, Quirino A, Peronace C, Settembre P, Marano V, Loria MT, Marascio N, Galati L, Barreca GS, Giancotti A, Amantea B, Liberto MC, Focà A. Soluble CD14 Subtype-A New Biomarker in Predicting the Outcome of Critically Ill Septic Patients. Am J Med Sci. 2017, 353(6):543-551

## P28 To reduce the mortality in septic shock could require not only Polimyxin B immobilized fiber column direct hemoperfusion therapy also other treatment

### Masasih Osaka, Kazuma Shibahara, Makiko Saito, Mihoko Tsunoda, Miri Kang, Takuya Oshiro, Noriko Saito, Munekazu Takeda, Arino Yaguchi

#### Department of Critical Care and Emergency Medicine, Tokyo Women’s Medical University, Tokyo, Japan

##### **Correspondence:** Arino Yaguchi (ayaguchi@twmu.ac.jp)

Background

Polimyxin B immobilized fiber column direct hemoperfusion (PMX-DHP) (ToraymixinTM, Toray Medical Co., Ltd., Tokyo, Japan) treatment has recently been shown to reduce their mortality in some population in septic shock as a result of a randomized placebo-controlled trials (such as EUPHRATES trial), although did not reach statistical significance of primary endpoint.

Objectives

Our hypothesis is not only to PMX-DHP therapy might also other therapies might need to improve the mortality in septic shock.

Methods

From November 2014 to December 2017, all adult patients treated with PMX-DHP and EAA (Endtoxin Activity Assay) showed ≥ 0.60 EAA units at pre-treatment of PMX-DHP in our medico-surgical ICU of university hospital were included in this retrospective study. Patients were divided into two groups, 1) patient with ≥ 10 MOD score and 2) patient with < 10 MOD score. EAA between before and after PMX-DHP therapy, SOFA score, P/F ratio, mean arterial pressure (MAP) at pre-treatment of PMX-DHP, platelet counts, total bilirubin, creatinine, age, sex 28 day mortality and 90 day mortality were compared between two groups. Patients’ data were collected from medical archives. Values were expressed as mean ± SD. Data was analyzed by chi-square test and Mann-Whitney U test. P values less than 0.05 were considered significant.

Results

Twenty-three patients (12 men, 11 women; age [mean 66.5 ± 13.0]) were included. SOFA score, MAP, P/F ratio, platelet counts, creatinine, 28-day mortality and 90-day mortality were significantly differences between patient s with ≥ 10 and with < 10 MOD score (16.3 ± 2.0 vs. 7.8 ± 3.5, 55.1 ± 3.9 vs. 70.7 ± 15.9, 127.1 ± 56.3 vs. 293.6 ± 157.6, 7.3 ± 4.8 vs. 16.3 ± 11.6, 3.4 ± 1.7 vs. 1.1 ± 1.4, 75% vs. 27%, 75 % vs. 53 %, p < 0.05, respectively). EAA units between before and after PMX-DHP therapy, total bilirubin, age, and sex did not show significant differences (0.75 ± 0.1 vs. 0.72 ± 0.1, 0.56 ± 0.26 vs. 0.44 ± 0.17, 2.0 ± 2.4 vs. 1.5 ± 2.1, 58.6 ± 12.0 vs. 66.5 ± 13.0, 75% vs. 40%, p > 0.05, respectively).

Conclusions

The present study shows PMX-DHP therapy decreased EAA units after that treatment. However, severe organ dysfunctions, such as coagulopathy, AKI or ARDS, are complicated, only removal of endotoxins does not contribute to the mortality. Not only PMX-DHP therapy but also other treatment could be required to improve the mortality.

## P29 Malnutrition and its clinical outcomes among sepsis patients admitted at Kigali university teaching hospital- Rwanda

### Mushuru Evariste^1^, Cameron Page^2^, Tim Walker^3^

#### ^1^Department of internal medicine, University teaching hospital/school of medicine and health sciences/university of Rwanda, Kigali, Rwanda; ^2^University Hospital of Brooklyn, SUNY Downstate Medical Center,450 Lenox Road, Brooklyn, NY 11226, USA; ^3^Calvary Mater Hospital, University of Newcastle, New South Wales, Australia, 2298

##### **Correspondence:** Mushuru Evariste (museva15@yahoo.fr)

Background

Malnutrition has been associated with high mortality and prolonged hospital stay in other populations [1,2]. The impact of malnutrition among sepsis patients in Rwanda has not yet been studied. This study aimed to study malnutrition associated outcomes in sepsis patients admitted at Kigali university teaching hospital (CHUK)- Rwanda.

Materials and methods

We screened 239 consecutive patients with sepsis who were admitted at CHUK from January 2016 through February 2016, 2 subjects were excluded (one withdrew consent and another was found to be pregnant). Using the Subjective Global Assessment (SGA) tool, we classified patients as well-nourished or as suffering from moderate or severe malnutrition. Patients’ anthropometric data, socio-economic and clinical data on sepsis were collected using a pre-established questionnaire. Clinical data were collected using patient’s files whereas data on weight loss, appetite, functional status, were completed through patient’s interview or next of kin for patients who were unable to communicate at the time of data collection.

Results

Of 237 total patients, 90(38%) patients were malnourished, with 64(27%) having moderate malnutrition and 26(11%) having severe malnutrition. The prevalence of malnutrition was higher in males as compared to females (47.3% vs 32.2%, p=0.020). 47 (19%) patients had known positive HIV serology and this was associated with malnutrition (p=0.005), 31(34.4%) patients were HIV negative. Malnourished patients lost on average 1.1kg during their hospital stay and well-nourished patients lost 0.6kg on average and the difference was statistically significant (p=0.003). Malnourished patients had longer hospital stay when compared to well-nourished patients, with a difference of 12.3days (28.7days for a malnourished patient vs 16.4days for a well-nourished patient (p<0.001). The likelihood of dying in malnourished patients is 2.3 times higher, as compared to normal patients (OR = 2.3, 95%CI: 1.25-2.49, p= 0.008).

Conclusions

Malnutrition among sepsis patients is common and is associated with prolonged hospital stay and high mortality. Further research is necessary to determine if early screening and improved management can improve these outcomes

Acknowledgements

1.University of Utah which partially financed the study

2.Kigali University Teaching Hospital where the study was conducted

References

1. Allison SP. Malnutrition, disease, and outcome. Nutrition 2000 Jul; 16(7-8): 590–3

2. Kristina N., Claude P., Herbert L., Matthias P.Prognostic impact of disease-related malnutrition.

Clinical Nutrition (2008)27,5–15

## P30 CYP1A1-12(S)HETE-JNK-AP-1 signaling axis strengthens inflammatory responses in overactivated macrophages of sepsis

### Lixing Tian, Xiaoyuan Ma, Xiaoying Zhou, Qi Huang, Junyu Zhu, Li Luo, Wanqi Tang, Wei Ma, Jing Yu, Xue Yang, Jun Yan, Huaping Liang

#### State Key Laboratory of Trauma, Burn and Combined Injury, Research Institute of Surgery, The Army Medical University, Chongqing, People’s Republic of China

##### **Correspondence:** Lixing Tian (tlxtmmu@163.com)

Background

Cytochrome P450 1A1 (CYP1A1), a kind of hydroxylase, plays a critical role in oxidative stress injury and mycoplasma infection. However, the relationship between CYP1A1 and inflammation, especially sepsis, remains poorly understood.

Materials and methods

(1) *Ex vivo* experiments, peritoneal macrophages isolated from wild type or Ahr-deficient (Ahr-/-) mice were treated with lipopolysaccharide(LPS) or heat-killed *E.coli*, and the mRNA and protein levels of CYP1A1 were determined.

(2) CYP1A1-overexpression and CYP1A1-loss-enzymatic-function macrophages were adaptively transferred to wild type mice challenged by LPS and CLP to observe the survival and illustrate the molecular mechanism.

(3) The level of CYP1A1 in human peripheral blood monocytes (PBMCs), isolated from healthy and septic individuals, were also detected by qRT-PCR and LSCM.

Results

CYP1A1 was highly expressed in mice peritoneal macrophages challenged by both LPS and heat-killed *E.coli*. Moreover, CYP1A1 and inflammatory factors were aberrant highly expressed in LPS challenged peritoneal macrophages from Ahr-/- mice compared with that from Ahr+/+ mice. Using CYP1A1-overexpression RAW264.7 cells (CYP1A1/RAW), we found that CYP1A1 could intensify macrophages inflammatory responses by up-regulating TNF-α and IL-6 through enhancing JNK/AP-1(c-fos and c-jun) phosphorylation rather than NF-κB (p65 and p50) phosphorylation. We discovered that CYP1A1/RAW secreted more 12(S)-HETE, while 12(S)-HETE antibody could attenuate intensified inflammatory responses caused by CYP1A1 overexpression. Administrating 12(S)-HETE alone into normal RAW264.7 cells could also reinforce inflammatory responses even without LPS stimulation. Furthermore, we showed that loss-of-enzymatic-function of CYP1A1 in macrophages impeded LPS induced JNK/AP-1 phosphorylation and inflammatory factors secretion due to impaired generation of 12(S)-HETE. Mice transferred with CYP1A1-overexpression but not with CYP1A1-loss-enzymatic-function macrophages were highly susceptible to LPS and CLP challenge. In addition, the CYP1A1 expression and 12(S)-HETE concentration levels were elevated in PBMCs from septic patients and had a positive correlation with Sequential Organ Failure Assessment (SOFA) score.

Conclusions

Altogether, these results revealed a novel signaling axis, namely CYP1A1-12(S)-HETE-JNK-AP-1, regulating macrophages inflammatory responses in sepsis, which could be a promising target for preventing and treating sepsis.

## P31 12(S)-HETE: a potential biomarker for sepsis?

### Qi Huang, Jun Yan, Liping Yang, Xingyu Wang, Junyu Zhu, Lixing Tian, Xiaoyuan Ma, Yu Sun, Kuan Liu, Xue Yang, Wei Ma, Huaping Liang

#### State Key Laboratory of Trauma, Burn and Combined Injury, Research Institute of Surgery, The Army Medical University, Chongqing, People’s Republic of China

##### **Correspondence:** Huaping Liang (13638356728@163.com)

Background

12(S)-HETE is the metabolite of arachidonic acid catalyzed by 12-lipoxygenase or cytochromeP*450*, which is implicated in hepatic ischemia-reperfusion injury, tumor metastasis and diabetes etc. However, whether or not it is correlated with sepsis remains unclear.

Materials and methods

1. 12(S)-HETE levels were determined in the supernatant of LPS-stimulated macrophages and in the plasma from mice injected intraperitoneally with LPS or faeces.
2. Inflammatory mediator TNF-α, IL-6 were determined in the supernatant of macrophages and mice plasma stimulated with 12(S)-HETE, and the in vivo pro-inflammatory response of 12(S)-HETE was observed when it was simultaneously used with LPS (i.p) inducing septic mice.
3. 12(S)-HETE, TNF-α, IL-6 plasma levels were detected in septic patients and healthy volunteers and their relationship with Sequential Organ Failure Assessment (SOFA) score was investigated.

Results

Our study showed that 12(S)-HETE production in LPS-activated macrophages was increased and its plasma level was elevated in septic mice induced by LPS or faeces (i.p) respectively. When added into macrophages culture system without LPS stimulation, 12(S)-HETE significantly enhanced the production of TNF-α, IL-6 in a dose-dependent manner. In vivo administration of 12(S)-HETE (i.p) also strengthened pro-inflammatory response and organs injury in septic mice, which is evidenced by increased TNF-α, IL-6 levels and more serious lung and liver pathological injury and lower survival rate. When compared with healthy control, the plasma levels of 12(S)-HETE, TNF-α, IL-6 were elevated in septic patients, and 12(S)-HETE concentrations of septic patients showed a significant positive correlation with SOFA score, (*r*=0.850, *P*<0.002).

Conclusion

It is suggested that 12(S)-HETE possesses pro-inflammatory effect and is positively correlated to SOFA score in septic patients, which might be a potential biomarker for the assessment of sepsis outcomes and the possible therapeutic target for the treatment of sepsis.

## P32 Personalized approach to early prediction of infectious complications in patients with severe trauma

### Viktor V. Moroz^1,3^, Artem N. Kuzovlev^1,3^, Ekaterina A. Boeva^3^, Alij K. Zhanataev^2^, Andrey D. Durnev^2^, Vasiliy I. Reshetnyak^1^

#### ^1^V.A. Negovsky Research Institute of General Reanimatology of the Federal Research and Clinical Center of Intensive Care Medicine and Rehabilitology, Moscow, Russia; ^2^V.V. Zakusov Research Institute of Pharmacology, Moscow, Russia; ^3^A.I.Evdokimov Moscow State University of Medicine and Dentistry, Moscow, Russia.

##### **Correspondence:** Artem Kuzovlev (artem_kuzovlev@mail.ru)

Background

The aim of the study was to develop a method for early personalized prognostication of the appearance of infection complications in patients with severe trauma and blood loss.

Materials and methods

28 patients with severe trauma were examined (15 men, 13 women; 35.3±13.2 y.o.). To assess the effect of hypoxia on DNA damage and processes of white blood cell (WBC) death victims were split into two groups, taking into account the values of 4 indices: capillary pO2, lactate, pH, BE. Group 1 - "Hypoxia" + "- 18 victims (4 indexes represented hypoxia on admission); group 2 - "Hypoxia" - "- 10 victims (4 indexes within the normal range). Two subgroups were distinguished in each group: infection "+" (13 patients in group 1 and 9 in group 2) and infection "-" (5 patients in group 1 and 1 in group 2). DNA damage in WBC as well as indirect apoptosis and necrosis of leukocytes were evaluated by DNA comet method. Analysis was done by epifluorescence microscope (Lomo, Russia, *200-400). Images of DNA comets were analyzed using the CASP 1.2.2 software. Percentage of DNA in the tail of the total amount of DNA in the comet was used as a quantitative indicator of DNA damage. Statistics was done by Microsoft Excel and Statistica 6.0; logistic regression was used.

Results

In trauma patients and hypoxia there was an increase in leukocytes death via apoptosis and necrosis, accompanied by an increase in single-, double-strand DNA breaks (DSB). On the 3rd day after trauma change in these indicators in group 1 differed between the infection "-" and infection "+" by the rate of infection complications developed on the 5th-7th day of follow-up. On the 3rd day after the trauma level of necrotic [8.7 (2.3; 14.7)%], apoptotic DNA comets [4.3 (2.5; 7.2)%] and single-, DSB [27.3 (21.4; 32.5)%] in patients with the hypoxia and infection "-" was higher than in group with the hypoxia "+" and infection "+" [6.7 (4.5; 12.4)%; 4.3 (2.6, 7.2)%; 13.4 (10.2, 18.6)%, respectively]. ROC analysis of the total score (necrotic DNA comets + apoptotic DNA comets + single-, DSB) obtained on the 3rd day after trauma in the group hypoxia "+" revealed that area under the ROC curve was 0.923 (sensitivity 100%, specificity 75,0%; cut-off value 47.3%). Values of the total indicator below 47.3% indicated a high risk of development of infection complications.

Conclusions

Method of early personalized prognostication of the development of infection complications in patients with severe trauma and hypoxia was proposed. It included determination of the index summing up the percentage of DNA-apoptotic comets, DNA-necrotic comets and single-, double-strand DNA breaks of leukocyte.

## P33 Identification of endogenous bacteriophages in patients with bacteremia and sepsis - an observational study

### Sergey S. Petrikov^1^, Aslan K. Shabanov^1,2^, Tatiana V. Chernenkaya^1^, Elena B. Lazareva^1^, Natalia V. Evdokimova^1^, Evgeny L. Zhilenkov^3^, Artem N. Kuzovlev^2^, Arkady M. Goloubev 2, Andrey Grechko^2^

#### ^1^N.V. Sklifosofsky Research Institute of Emergency Medicine, Moscow, Russia; ^2^V.A. Negovsky Research Institute of General Reanimatology of the Federal Research and Clinical Center of Intensive Care Medicine and Rehabilitology, Moscow, Russia; ^3^OOO SPC "MicroMir", Moscow, Russia

##### **Correspondence:** Artem Kuzovlev (artem_kuzovlev@mail.ru)

Background

There are still limited data on the role of human endogenous bacteriophages in the prevention and treatment of infectious complications and sepsis in critically ill patients. The aim of this study was to investigate into the influence of endogenous bacteriophages on the mortality is intensive care unit patients with bacteremia and sepsis.

Materials and methods

25 intensive care unit patients with severe combined trauma were enrolled in this observational single-center study (8 women, 17 men; 48.3±18.3 y.o.). 28 blood samples were collected. Blood cultures were performed using an automated Bactec-9050 blood culture analyzer (Becton Dickinson, USA). Identification of the isolated microorganisms was carried out using an automatic analyzer WalkAway-40 (Beckman Coulter, USA). In order to isolate endogenous bacteriophages patients were sampled for blood and urine cultures. Proceeding of bacteriophages was performed by means of traditional virological methods. Bacteriophages extracted from the lysis zones after spot testing were examined on an electron microscope JEOL-1011 (Japan). Statistics was done by Microsoft Excel and Statistica 6.0, X2 criterion was used.

Results

Out of 25 patient’s bacteriophages were isolated in 10. Blood microbiology was: in 4 cases *Klebsiella pneumoniae*, in 3 - Acinetobacter sp., in 2 - Staphylococcus spp., 1 case of *S. aureus* and *Enterococcus faecalis*. In the group of patients who had their own endogenous bacteriophages 4 people died (40%). At the same time 2 patients had their own phages identical to the isolated microorganisms (*Staphylococcus aureus* and *S. epidermidis*) which were then eliminated. Later on these patients isolated strains of *K. pneumonia* to which endogenous phages were absent and patients died due to this infection. Out of 15 patients who did not have endogenous bacteriophages 9 (60%) died (p <0.002). Blood cultures in these 15 patients were: 5 cases - *S. aureus*, 2 - Staphylococcus sp., 5 - *K. pneumonia*, 2 - Acinetobacter sp. and in 1- *Enterobacter aerogenes*.

Conclusions

Endogenous bacteriophages influence the mortality of trauma patients with bacteremia and sepsis: mortality was significantly higher in the absence of endogenous bacteriophages (p <0.002).

## P34 Inhibition of sphingosine kinase 1 for protection of gut barrier function in sepsis

### Jeffery Ho, Stephen Liang, Xiaodong Liu, Lin Zhang, William KK Wu, Matthew TV Chan, Czarina CH Leung

#### Department of Anaesthesia and Intensive Care, Prince of Wales Hospital, The Chinese University of Hong Kong, Hong Kong

##### **Correspondence:** Czarina CH Leung (czarinaleung@cuhk.edu.hk)

Background

The intestinal epithelium is a continuous single-cell layer separating the sterile bloodstream from the gut microbes. Accumulating evidence suggests that this barrier could be substantially impaired during severe systemic inflammation. Importantly, the severity of derangement of intestinal permeability in critically ill patients predicts subsequent development of multiple organ failure and its regulation by host factors remains unclear. Finding ways to maintain the intestinal epithelial integrity is one of the major directions to improve the clinical outcome of sepsis

Materials and methods

We performed cecal-ligation and puncture on C57BL/6 mice to induce polymicrobial sepsis. Animals undertook either sham surgery with saline injection, CLP with N,N-dimethylsingosine (a potent inhibitor of sphingosine kinase 1), or CLP with saline injection. At 20h, the animals were orally fed with 4kDa fluorescein-dextran (FITC). At 24h after surgery, we collected blood and harvested ileal tissues for molecular characterization, including transcriptomic analysis.

Results

Of septic mice, intestine exhibited remarkable pathological changes, including increased epithelial apoptosis, reduced expression of tight junction proteins, and elevated serum FITC level. Transcriptomic profiling revealed that sphingosine kinase 1 increased more than six-fold after sepsis-induction. Pathway enrichment analysis revealed significant pathways pertinent to pro-inflammatory response. Animals with pharmacological inhibition using N,N-dimethylsphingosine had lower level of serum FITC, less severe sepsis symptoms and lower total bacterial DNA in the blood.

Conclusions

Inhibition of sphingosine kinase 1 may be a therapeutic strategy for preventing gut barrier dysfunction in sepsis. Further preclinical studies will be needed to confirm the beneficial effect in prior to clinical trials.

Acknowledgements

This study was funded by Health and Medical Research Fund of Hong Kong SAR (05160746; 16151002) and Australian and New Zealand College of Anaesthetists (18/019).

## P35 Prognostic modeling of inflammatory correlates of mortality and persistence in complicated *S. aureus* bacteremia

### Alessander O. Guimaraes^1^†, Yi Cao^1^†, Kyu Hong^1^, Oleg Mayba^1^, Melicent Peck^1^, Johnny Gutierrez^1^, Felicia Ruffin^3^, Montserrat Carrasco-Triguero^1^, Jason B. Dinoso^1^, Angelo Clemenzi-Allen^2^, Catherine A. Koss^2^, Stacey Maskarinec^3^, Henry F. Chambers^2^, Vance G. Fowler, Jr.^3^, Amos Baruch^1^, Carrie M. Rosenberger^1^

#### ^1^Department of Biomarker Discovery, Genentech, Inc., South San Francisco, CA 94080, USA; ^2^Division of HIV, Infectious Diseases, and Global Medicine, University of California San Francisco, San Francisco, CA, USA; ^3^Division of Infectious Diseases, Duke University, Durham, NC, USA.

†These authors contributed equally to this work.

##### **Correspondence:** Carrie Rosenberger (rosenberger.carrie@gene.com)

Background

Dysregulated inflammatory responses can lead to host-driven sequelae such as sepsis, as well as an immune response not optimally tailored for bacterial clearance. Heterogeneity in the host response to invasive bacterial infection suggests that specific biomarker signatures could be utilized to differentiate patients prone to severe disease, thereby facilitating earlier implementation of more aggressive therapies. To further elucidate the inflammatory correlates of poor clinical outcomes in bacteremic patients, we evaluated the association between a large panel of blood proteins at initial presentation of bacteremia and disease severity outcomes.

Materials and methods

We conducted an observational study (n=32) to evaluate the prognostic value of 64 circulating protein biomarkers for mortality or persistent bacteremia (defined as positive blood cultures for >5 days) in patients with *S. aureus* bloodstream infections. Prioritized biomarkers were evaluated in a case-control study of 156 patients with *S. aureus* or Gram-negative bloodstreams infections using serum samples collected within 1-3 days of empiric antibiotic therapy.

Results

We identified 8 candidate biomarkers of inflammation and endothelial activation that were prognostic for mortality and relevant to both Gram-positive and Gram-negative bacteremia. Statistical modeling identified that the combination of the chemokines IL-8 and CCL2 had the best power to classify fatal outcome and was superior to routine clinical laboratory and demographic metrics. A distinct set of 3 serum proteins was elevated early in patients with delayed clearance of bloodstream infection, with high baseline IL-17A showing the strongest correlation with the duration of positive blood cultures and endovascular infection foci.

Conclusions

These results identify immune correlates of delayed clearance of bloodstream infection and mortality in patients treated with appropriate antibiotics. These biomarkers are being evaluated in a validation cohort to evaluate their clinical utility for distinguishing patients with the highest risk for treatment failure and potential for selecting patients for clinical trials.

## P36 Pellino3 impacts sepsis-mediated IL1b secretion in peritoneal macrophages

### Laura Kuchler, Annika K Giegerich, Bernhard Brüne and Andreas von Knethen

#### Institute of Biochemistry I – Pathobiochemistry, Faculty of Medicine, Goethe–University Frankfurt, Theodor–Stern–Kai 7, 60590 Frankfurt, Germany

##### **Correspondence:** Laura Kuchler (kuchler@biochem.uni-frankfurt.de)

Background

Autophagy plays a central role in controlling innate immune responses by degradation of proteins. Autophagic factors like p62/SQSTM1 directly interact with molecules of the Toll-like receptor 4 (TLR4) pathway [1], which is activated in macrophages in response to lipopolysaccharide (LPS) [2]. In this signaling cascade Pellino3 serves as an IRAK E3 ligase and scaffold protein [3]. Its autophagy-dependent degradation impairs the hyperinflammatory phase during LPS challenge by inhibiting TLR4 signaling and IL1b expression [4]. Based on these *in vitro* data, we want to translate this setting in an *in vivo* cecal ligation and puncture (CLP) mouse model to substantiate the modulation of Pellino3 expression during sepsis as a new strategy for the development of a therapy approach.

Materials and methods

We used cecal ligation and puncture (CLP) to induce polymicrobial sepsis in wildtype mice to monitor Pellino3 expression. 6h/24h/48h following sepsis induction peritoneal macrophages and peritoneal fluid were harvested. Pellino3 and Il1b mRNA were analysed by RT-PCR. Pellino3 as well as LC3, p62 and pro-IL1b protein expression was elucidated by western analysis. IL1b levels in the peritoneal fluid were determined by cytometric bead array (CBA) via FACS analysis.

Results

We observed an initial increase of Pellino3 protein expression in peritoneal macrophages of wildtype mice after 24 h following CLP. This is accompanied by an increase of LC3-II, suggesting autophagy inhibition, and an increase in pro-IL1b. Concomitantly, IL1b secretion in the peritoneal fluid is elevated. 48h after CLP Pellino3 is degraded, most likely due to increased autophagy as shown by simultaneously degradation of LC3-II. In lysates of peritoneal macrophages pro-IL1b protein expression is decreased and consequently in supernatants of these cells IL1b is attenuated.

Conclusions

Our *in vivo* data confirmed our in vitro findings of a correlation of Pellino3 and its degradation, impairing IL1b expression and secretion. The proof that autophagic processes are involved in Pellino3 degradation is still elusive in the mouse *in vivo* model. Therefore, experiments in autophagy-deficient mice using a p62-knockout model are planned for the near future. Moreover, the induction of autophagy at different timepoints during sepsis progression will help to confirm the mechanism. Since an overwhelming proinflammatory response is a main problem during sepsis, targeting Pellino3 by considering the knowledge of autophagy induction should be approached and extended in the future as a new therapy regime.

References

1. Levine B, Mizushima N, Virgin HW: Autophagy in immunity and inflammation. Nature. 2011; 469(7330):323-335.

2. Jiankun Zhu, Chandra Mohan: Toll-like Receptor Signaling Pathways – Therapeutic Opportunities. Mediators Inflamm. 2010; 2010:781235.

3. Schauvliege R, Janssens S, Beyaert R. Pellino proteins are more than scaffold proteins in TLR/IL-1R signalling: a role as novel RING E3-ubiquitin-ligases. FEBS Lett. 2006; 580(19):4697-4702.

4. Giegerich AK, Kuchler L, Sha LK, Knape T, Heide H, Wittig I, Behrend C, Brüne B, and von Knethen A Autophagy-dependent PELI3 degradation inhibits proinflammatory IL1B expression. Autophagy 2014; 10(11): 1937–1952.

## P37 Improved selective peroxisome proliferator-activated receptor gamma modulators (SPPARγMs) for treatment of sepsis

### Tilo Knape^1^, Victor Hernandez-Olmos^1^, Daniel Flesch^2^, Mario Wurglics^2^, Eugen Proschak^1, 2^, Jan Heering^1^, Mukul Ashtikar^1^, Matthias G Wacker^1, 3^, Manfred Schubert Zsilavecz^2^, Dieter Steinhilber^1, 2^, Bernhard Brüne^1, 4^, Nerea Ferreiros-Bouzas^1, 5^, Monica Villa Nova^3^, Sandra Labocha^5^, Michael J Parnham^1^, Andreas von Knethen^1, 4^

#### ^1^Branch for Translational Medicine and Pharmacology TMP, Fraunhofer Institute for Molecular Biology and Applied Ecology IME, Frankfurt, Germany; ^2^Institute of Pharmaceutical Chemistry, Goethe University Frankfurt, Frankfurt, Germany; ^3^Institute of Pharmaceutical Technology, Goethe University Frankfurt, Frankfurt, Germany; ^4^Institute of Biochemistry I - Pathobiochemistry, Goethe University Frankfurt, Germany; ^5^Institute of Clinical Pharmacology, Goethe University Frankfurt, Frankfurt, Germany

##### **Correspondence:** Tilo Knape (tilo.knape@ime.fraunhofer.de)

Background

Peroxisome proliferator-activated receptor gamma (PPARγ) has been shown to play a crucial role in immunosuppression during sepsis. Activated PPARγ is upregulated in T cells of septic patients, sensitizing these cells to PPARγ dependent apoptotic depletion [1]. In the mouse cecal ligation and puncture (CLP) sepsis model, both T cell-specific PPARγ gene knockout and systemic pharmacological PPARγ antagonism by GW9662 improved survival [2, 3]. As GW9662 it is not suitable for therapeutic use in humans, we developed and improved a new class of selective PPARγ modulators (SPPARγMs) for use in the treatment of sepsis in humans [4].

Materials and methods

To design improved SPPARγMs, initial three-dimensional computational modeling was performed. Potential candidates were synthesized and kinetically characterized in vitro using PPARγ-dependent transactivation assays in human cell lines. Cytotoxic effects of promising compounds were tested in cell viability assays. The intracellular accumulation of suitable compounds in the same cell lines was assessed by mass spectrometry. To verify the potential modulating effects of compounds on PPARγ target genes, gene expression was studied using quantitative real-time polymerase chain reaction (qPCR). Based on their in vitro properties, promising compounds were further investigated for their in vivo pharmacokinetic properties. Compounds fulfilling in silico, *in vitro* and *in vivo* requirements were finally tested in the mouse CLP sepsis model.

Results

In an initial screen, T-10017 was identified, from structurally related chemical compounds, as a potential lead compound for a new class of competitive, reversible PPARγ antagonists. In the mouse CLP sepsis model, administration of T-10017 nanoparticles led after 48 h to a significant improvement in the survival of septic mice, compared with control mice, treated with unloaded nanoparticles. Starting from T-10017, we further optimized the compound properties by chemical modification, including agonistic and antagonist characteristics, solubility and stability, resulting in two derivatives with markedly improved *in vitro* and *in vivo* properties.

Conclusions

Compounds from our new class of SPPARγMs are significantly effective in the treatment of murine sepsis. These compounds competitively inhibit PPARγ dependent T cell depletion, help to maintain immune competence, facilitate the controlled therapeutic regulation of hypoinflammation and thus, reduce severity and mortality of sepsis.

Acknowledgements

This research was supported by the research funding program Landes-Offensive zur Entwicklung wissenschaftlich-ökonomischer Exzellenz (LOEWE) of the State of Hesse, Germany, the Else Kröner-Fresenius foundation training program “Translational Research Innovation – Pharma” (TRIP) and the Collaborative Research Center 815 “Redox-Regulation” (SFB815).

References

1. Soller M, Tautenhahn A, Brüne B, Zacharowski K, John S, Link H, von Knethen A. Peroxisome proliferator-activated receptor gamma contributes to T lymphocyte apoptosis during sepsis. J Leukoc Biol 2006, 79:235-243.

2. Schmidt MV, Paulus P, Kuhn AM, Weigert A, Morbitzer V, Zacharowski K, Kempf VA, Brüne B, von Knethen A. Peroxisome proliferator-activated receptor γ-induced T cell apoptosis reduces survival during polymicrobial sepsis. Am J Respir Crit Care Med 2011, 184:64-74.

3. Leesnitzer LM, Parks DJ, Bledsoe RK, Cobb JE, Collins JL, Consler TG, Davis RG, Hull-Ryde EA, Lenhard JM, Patel L et al. Functional consequences of cysteine modification in the ligand binding sites of peroxisome proliferator activated receptors by GW9662. Biochemistry 2002, 41:6640-6650.

4. Knape T, Flesch D, Kuchler L, Sha LK, Giegerich AK, Labocha S, Ferreirós N, Schmid T, Wurglics M, Schubert-Zsilavecz M, Proschak E, Brüne B, Parnham MJ, von Knethen A. Identification and characterisation of a prototype for a new class of competitive PPARγ antagonists. Eur J Pharmacol 2015, 755:16-26.

## P38 The process of septic shock is attenuated by the intravenous administration of peptide VSAK: A FDG-PET study

### Ismael Luna-Reyes^1^, Eréndira Pérez-Hernández^1^, Miguel Ángel Ávila-Rodríguez^2^, Jaime Mas-Oliva^1^

#### ^1^Instituto de Fisiología Celular, Universidad Nacional Autónoma de México, Mexico City, México; ^2^Facultad de Medicina, Universidad Nacional Autónoma de México, Mexico City, México

##### **Correspondence:** Jaime Mas-Oliva (jmas@ifc.unam.mx)

Background

The development of an uncontrolled inflammatory response is considered the main responsible of the most deleterious effects during severe sepsis and septic shock, that finally can lead to the condition known as Multiorgan Dysfunction Syndrome (MODS). The inflammatory response is triggered by pathogen derived molecules, mainly lipopolysaccharides (LPS). LPS and other pathogen molecules are released in early stages of sepsis and recognized by Pattern Recognition Receptors (PRR), directly involved in the initiation of the inflammatory response. In previous work, we have demonstrated in vitro and in vivo that VSAK, a peptide derived from the last eighteen aminoacids of the carboxy-end segment of CETPI, considered an isoform of the cholesteryl-ester transfer protein (CETP) [1], is able to bind LPS and therefore, neutralize their harmful effects [2]. The present study provides additional evidence supporting the protective effects of VSAK administration in rabbits induced to a septic-shock-like state employing Positron Emission Tomography (PET).

Materials and methods

During the experiment, we used ten male Dutch dwarf rabbits provided by a local certified farm. Rabbits were randomly assigned to four experimental groups: 1) Control, 2) VSAK treatment, 3) LPS treatment, and 4) LPS+VSAK treatment. Immediately after, rabbits were administered 2-[18F]fluoro-2-deoxy-D-glucose (FDG) as the radiotracer and monitored by PET. Each scan was performed during 70 minutes and PET data analysis carried out using spherical areas to define Volume of Interest (VOI) in order to reduce slight variations associated with differences in animal size.

Results

Figure 1 shows PET images from four representative rabbits, corresponding to each experimental group. In general, a marked decrease of glucose uptake can be observed in the LPS group. Results obtained from the normalized Standard Uptake Value (SUV) taking into account a spherical area of 5.5 mm in all four experimental groups are shown in Fig. 2. In all cases, SUV estimated in the first ten minutes was considered as 100% SUV and used to normalize individual data sets. A negative correlation between time and %SUV is observed in all experimental groups. The negative correlation is slightly more pronounced in the LPS group, decreasing up to 73.5% ±2.7 SEM at 70 minutes, while in the control group (83.43% ± 1.3 SEM), VSAK (83.99% ± 6.55 SEM) and VSAK-LPS (89.15% ± 2.09 SEM).

Conclusions

This study supports the potential in vivo use of peptide VSAK in order to neutralize the harmful effects of LPS and therefore avoid a metabolic dysfunction pattern during sepsis and septic shock [3].

Acknowledgements

We thank Blanca Delgado-Coello for expert technical assistance. We thank Conacyt for grant 255778 awarded to J.M-O.

References:

1) Alonso AL, Zentella-Dehesa A, Mas-Oliva J. Characterization of a naturally occurring new version of the cholesterol ester transfer protein (CETP) from small intestine. Mol Cellular Biochem 245: 173-182 (2003).

2) García-González V, Guitiérrez-Quintanar N, Mas-Oliva J. The C-terminus domain of CETPI defines its function as a new plasma lipopolysaccharide-binding protein. Sci Rep, 5; 2015 (doi:10.1038/srep16091).

3) International Patent Application PCT/MX2014/00087

**Fig. 1 (abstract P38).**
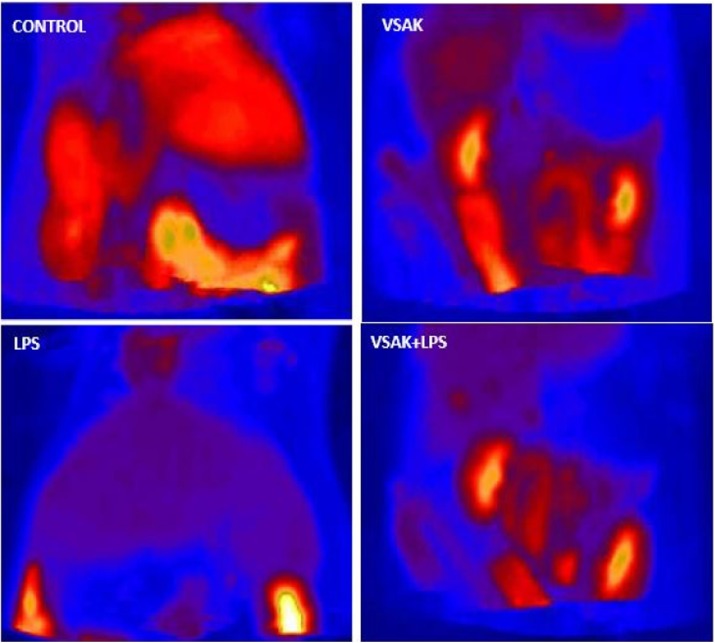
PET representative images of the abdominal cavity of each of the four experimental animal groups

**Fig. 2 (abstract P38).**
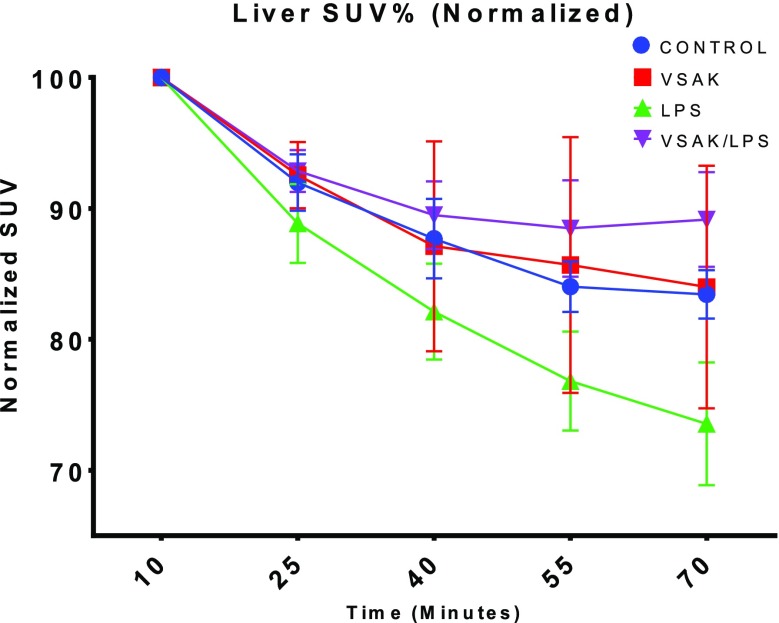
Standard Uptake FDG Values (SUV) from the four representative experimental animal groups

## P39 microRNA 330-3p controls permeability of the vascular endothelium by targeting essential junction proteins in sepsis related infection

### Rebecca L. Watkin^1^, Olga Piskareva^2^, Sudipto Das^2^, Stephen Madden^3^, Steve W. Kerrigan^1^

#### ^1^Cardiovascular Infection Research Group, Irish Centre for Vascular Biology, RCSI, Dublin 2, Ireland; ^2^Cancer Genetics Research Group, RCSI, Dublin 2, Ireland; ^3^Data Sciences Centre, RCSI, Dublin 2, Ireland

##### **Correspondence:** Rebecca L. Watkin (rebeccawatkin@rcsi.com)

Background

Sepsis patients commonly present with features including progressive subcutaneous and body cavity oedema, typically caused by permeabilization of the vascular endothelial monolayer. Strict endothelial barrier control is mediated by structures located at cell-to-cell contact sites known as endothelial tight junctions and adherens junctions. Of these junctions, VE-Cadherin is arguably the most important functional component. Previously we have demonstrated that bacterial attachment to the endothelium decreases VE-cadherin expression resulting in increased vascular permeability [1-3]. MicroRNAs (miRNAs) play a key role in maintaining normal endothelial cell function and their dysregulation has been linked to a number of clinically important diseases including cancer, myocardial infarction, neurodegeneration and infection [4]. This study aims to investigate the role of miRNAs in controlling VE-cadherin during infection, and to uncover their influence on barrier integrity and permeabilization of the vascular endothelial monolayer.

Materials and methods

Human endothelial cells were subjected to shear forces (10dynes/cm2), mimicking physiological conditions experienced in the vasculature[1-3]. Via *S. aureus* administration, miRNA alterations were uncovered and determined by Taqman array cards, generating miRNA profiles of both uninfected and infected cells (RQ = 2-ΔΔCt). miRNA candidates of interest were selected bioinformatically and confirmed via individual miRNA assays. Potential mRNA targets for the candidate miRNA chosen were established bioinformatically and confirmed by Next Generation RNAseq, western blots, flow cytometry, immunostaining and qPCR. Mirmimetics, antagomirs and siRNAs were applied to establish the signalling pathway triggered during the infection [1].

Results

Following *S. aureus* infection, 58 endothelial miRNA were significantly down- and 35 significantly up-regulated, including the target of interest, miR-330 (p<0.05). Bioinformatic analysis of Next Generation RNAseq data identified 102 potential miR-330 targets that were down-regulated following infection (p<0.005). Of interest were genes required for endothelial barrier integrity, including ADAM19 and ZO-1. During infection, miR-330 expression was inversely correlated with ADAM19 expression but not ZO-1 expression. Both *S. aureus* infection (p<0.05) and miR-330 overexpression (via miRmimetic transfection) in uninfected endothelial cells caused increased vascular permeability (p<0.005). siRNA analyses revealed that the increase in permeability was caused by an ADAM19 mediated VE-Cadherin depletion, initially triggered by the increase in miR330 expression.

Conclusions

Our results suggest that S. aureus infection leads to rapid increase in endothelial miR330, contributing to the loss of barrier integrity through down-regulation of the metalloproteinase ADAM19, which in turn leads to a decrease in essential junction protein VE-Cadherin. Preventing or blocking dysregulated miRNAs may hold promise as future therapeutics in the treatment of sepsis.

Acknowledgements

Prof. Steve Kerrigan, Prof. Ray Stallings, Dr. Olga Piskereva, Dr. Sudipto Das,

Dr. Stephen Madden, Dr. Carolina Garciarena, Dr. John Nolan, Dr. Shane O'Grady , Mr. Tony McHale, Mr. Glenn Fitzpatrick, Ms. Unwana Umagha, Ms. Fajer Yousef.

References

1. McDonnell C.J., Garciarena C.D., Watkin R.L., McHale T.M., McLoughlin A., Claes J., Verhamme P., Cummins P.M., Kerrigan S.W. Inhibition of major integrin alphaV beta3 reduces Staphylococcus aureus attachment to sheared human endothelial cells. J Thromb Haemost, 2016. 14(12): p. 2536-2547.

2. McHale, T.M., Garciarena C.D., Fagan R.P., Smith S.G.J., Martin-Loches I., Curley G.F., Fitzpatrick F., Kerrigan S.W. Inhibition of Vascular Endothelial Cell Leak Following Escherichia coli Attachment in an Experimental Model of Sepsis. Crit Care Med, 2018.

3. Garciarena, C.D., McHale T.M., Martin-Loeches I., and Kerrigan S.W. Pre-emptive and therapeutic value of blocking bacterial attachment to the endothelial alphaVbeta3 integrin with cilengitide in sepsis. Crit Care, 2017. 21(1): p. 246.

4. Watkin R.L., Fitzpatrick G.G., and Kerrigan S.W. The Evolving Role of MicroRNAs in Endothelial Cell Dysfunction in Response to Infection. Semin Thromb Hemost, 2018. 44(3): p. 216-223.

## P40 Stabilization of the vascular endothelium prevents bacterial spread in a rat pneumonia model of sepsis

### Sinéad Hurley^1^, Eric Rogers^1^, Fidelma Fitzpatrick^2^, Ger Curley^3^, Steve Kerrigan^1^

#### ^1^Cardiovascular Infection Research Group, Irish Centre for Vascular Biology, Royal College of Surgeons in Ireland, Dublin, Ireland; ^2^Department of Microbiology, RCSI, Beaumont Hospital, Dublin, Ireland; ^3^Anaesthesia and Critical Care, RCSI, Beaumont Hospital, Dublin, Ireland

##### **Correspondence:** Sinéad Hurley (sineadhurley@rcsi.com)

Background

Sepsis is associated with severe vascular endothelial cell dysfunction which rapidly leads to dysregulation of normal systemic hemostasis and vascular reactivity triggering widespread tissue oedema. Although this process is considered central to the progression to organ failure during sepsis there are no therapeutic options available to treat or prevent endothelial dysfunction. Using an ex-vivo model of sepsis, we recently demonstrated that inhibition of major vascular endothelial cell integrin aVb3 prevented endothelial dysfunction induced by a number of sepsis pathogens by stabilising the vascular endothelium [1,2,3].

Aims

This study investigated the therapeutic usefulness of blocking aVb3 to control vascular endothelial cell dysfunction and stabilize the endothelium in an Escherichia coli induced rat pneumonia model of sepsis.

Materials and methods

*Escherichia coli* (CFT073) was delivered directly into the lungs, via a 14G cannula, of anaesthetized Sprague Dawley rats. Cilengitide, a specific aVb3 inhibitor, was administered intravenously (IV). Following progression to sepsis animals were sacrificed, organs were harvested and homogenised. Bacterial load in the spleen, liver, kidney, lungs and blood was assessed by viable count assay. Biomarker lactate was measured by ELISA. Vascular permeability was assessed using Evans blue dye.

Results

All animals were sacrificed at 24hrs. Bacterial counts (CFU/ml) in the blood (Fig. 1) and all major organs including the liver (Fig. 2A), kidney (Fig. 2B), spleen (Fig. 2C) and lung (Fig. 2D) were significantly lower in animals treated with cilengitide (control vs cilengitide treated, P<0.05). We next investigated if cilengitide was stabilizing the vascular endothelium thus preventing the dissemination of bacteria from the lung into the bloodstream. To test this, control non-infected animals were injected with Evan blue dye. These animals did not leak dye from their blood vessels as they maintain vascular integrity. In contrast animals that developed sepsis from the pneumonia displayed significant leakage from their blood vessels. Most strikingly, animals that developed pneumonia and were treated with cilengitide, failed to leak dye from their blood vessels suggesting that their endothelium was stabilised by the cilengitide thus reducing vascular permeability (Fig. 3) (untreated vs cilengitide treated, P<0.01). Finally and most critically, there was a significant reduction in the serum biomarker lactate in the cilengitide treated animals versus control animals (P<0.01) (Fig. 4), suggesting recovery.

Conclusions

Our results suggest that stabilizing the vascular endothelium by targeting the major endothelial cell integrin aVb3, may prevent the dissemination of bacteria to the blood and all major organs thus preventing progression to sepsis.

References

1. McDonnell CJ, Garciarena CD, Watkin RL, McHale TM, McLoughlin A, Claes J, et al. Inhibition of major integrin alphaV beta3 reduces Staphylococcus aureus attachment to sheared human endothelial cells. J Thromb Haemost, 2016.14(12):2536-2547.

2. McHale TM., Garciarena CD, Fagan RP, Smith SG.J, Loeches IM, Curley GF., Fitzpatrick F, Kerrigan SW. Inhibition of Vascular Endothelial Cell Leak Following Escherichia coli Attachment in an Experimental Model of Sepsis .Crit Care Med, April 2018.

3. Garciarena CD, McHale TM, Loeches IM, et al: Pre-emptive and therapeutic value of blocking bacterial attachment to the endothelial alphaVbeta3 integrin with cilengitide in sepsis. Crit Care 2017.21(1):246–247

**Fig. 1 (abstract P40).**
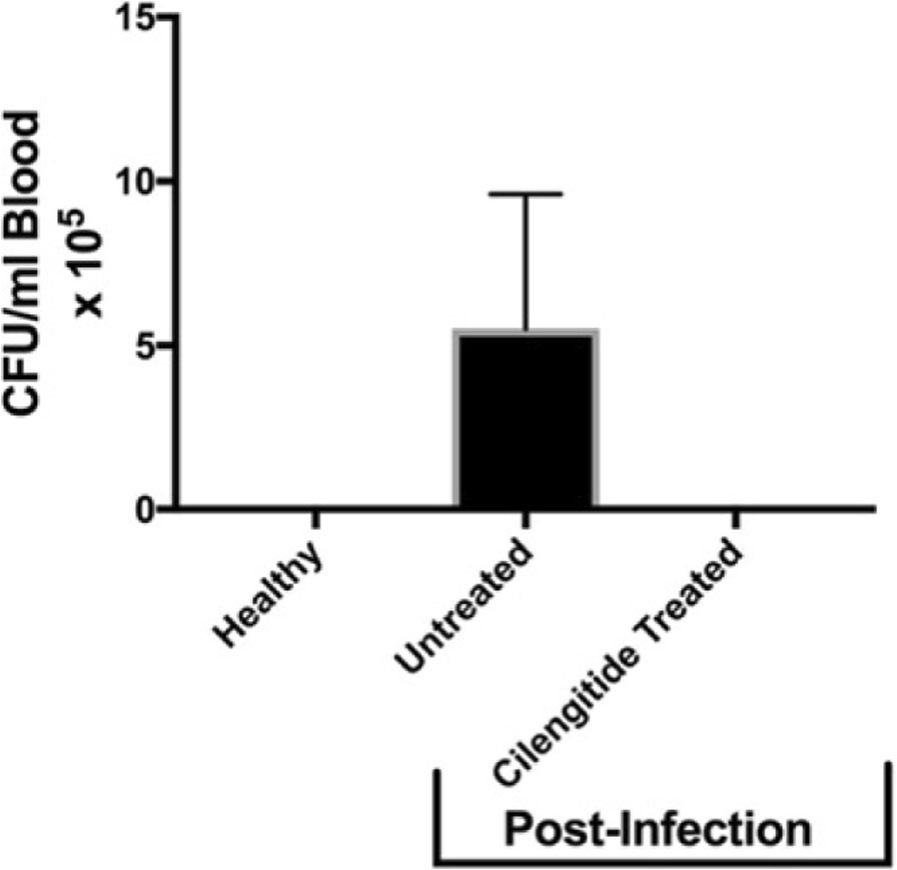
See text for description

**Fig. 2 (abstract P40).**
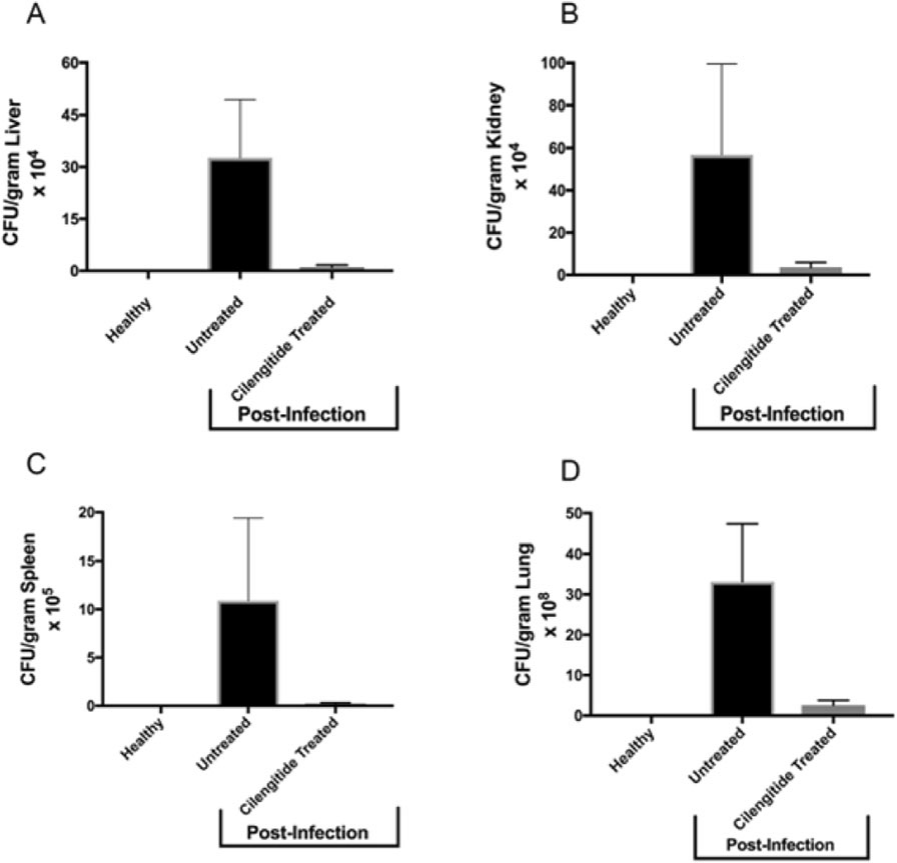
See text for description

**Fig. 3 (abstract P40).**
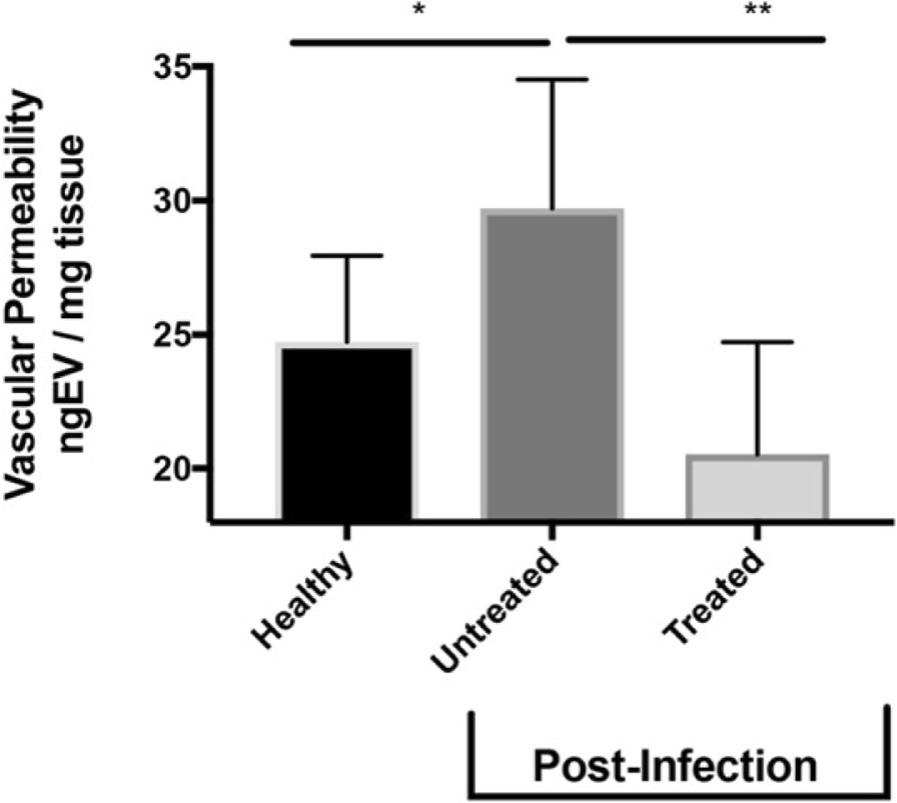
See text for description

**Fig. 4 (abstract P40).**
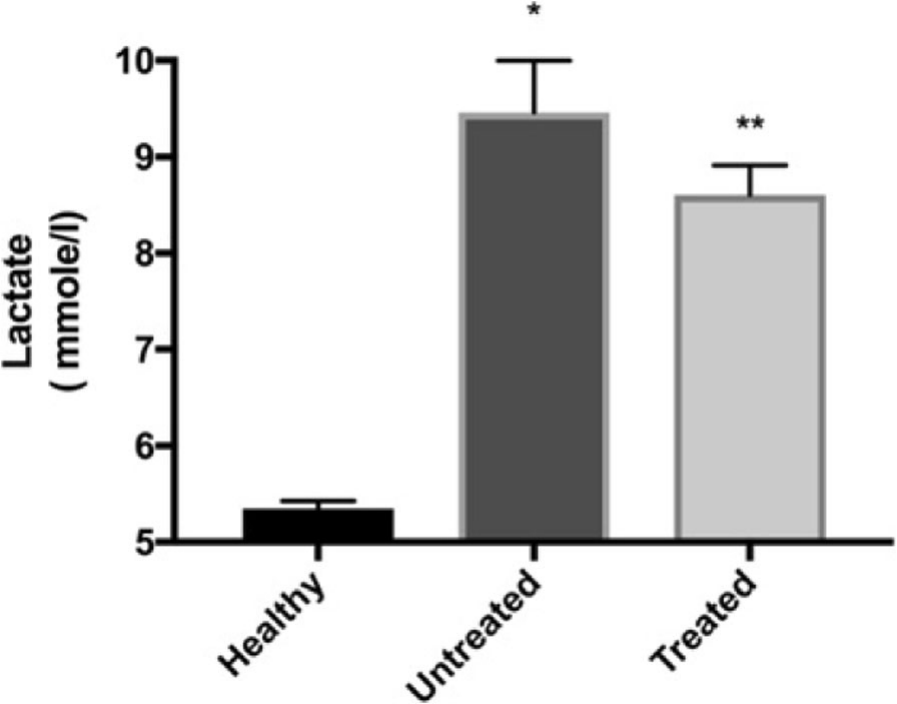
See text for description

## P41 Potential contribution of aromatic microbial metabolites to platelet aggregation disturbance

### Ekaterina Chernevskaya^1^, Natalia Beloborodova^1^, Alexander Bulychev^2^

#### ^1^Federal Research and Clinical Center of Intensive Care Medicine and Rehabilitology, Moscow, Russia; ^2^Federal Research Center "Computer Science and Control" of Russian Academy of Sciences (Institute of Systems Analysis)

##### **Correspondence:** Ekaterina Chernevskaya (chea05@inbox.ru)

Background

In patients with sepsis general platelet hypo-responsiveness to exogenous agonists is associated with mitochondrial dysfunction [1], but the mechanisms of this are not yet known. As shown previously, aromatic microbial metabolites (AMM) may influence on some mitochondrial functions [2]. The aim of this work is to evaluate the potential effect of aromatic metabolites on platelets aggregation in whole blood.

Materials and methods

We evaluated platelet aggregation (native and ASPItest) using the Mulitplate platelet function analyzer (Roche), a total of 112 tests in whole blood of healthy volunteers (n = 11). Additives (20 μl) of AMM were selected in clinically significant dosage as shown early in patients with bacterial infection and sepsis [3]. The following AMM: p-hydroxyphenyllactic (HPLA), p-hydroxyphenylacetic (HPAA), phenyllactic (PLA), phenylpropionic (PPA), benzoic (BA) acids were used at the first stage of the experiment (I). Complex mixtures of AMM were used at the second stage of the experiment (II): ∑PLA,HPAA,HPLA = 8,2 μM (II-A) ; ∑PLA, HPAA, HPLA = 17,5 μM (II-B), ∑PLA,HPAA,HPLA 8,2 μM +PPA 1,4 μM (II-C) versus lipopolysaccharide (LPS). To compare the data, the Sign Test was used for the median.

Results

There were no statistically significant dose-dependent effects of AMM on native platelet aggregation. A significant decrease of median ASPItest under the influence of HPAA 15 μM and HPLA 30 μM were identified on 27% and 19,5% respectively, that presumably allows to regard these acids as cyclooxygenase (COX) and IIb / IIIa inhibitors antagonists. Complex mixtures had a more pronounced antiaggregation effect, especially the mixture II-C, comparable to the impact of LPS.

Conclusions

Aromatic microbial metabolites have the potential to affect platelet aggregation *in vitro*, which requires further study.

Acknowledgements

Supported by Russian Science Foundation Grant №15-15-00110.

References

1. Protti A. et al. Platelet mitochondrial dysfunction in critically ill patients: comparison between sepsis and cardiogenic shock. Crit Care. 2015 Feb 11;19:39.

2.Fedotcheva N. et al.Toxic effects of microbial phenolic acids on the functions of mitochondria.Toxicol Lett. 2008 Aug 28;180(3):182-8.

3.Beloborodova N et.al.Normal level of sepsis-associated phenylcarboxylic acids in human serum. Biochemistry (Mosc). 2015 Mar;80(3):374-8.

## P42 Quantifying multi-dimensional time-dependent abnormality of the host response in a cohort of patients suspected of sepsis

### Ishan Taneja ^1,2^, Sihai Dave Zhao ^1^, Ruoqing Zhu ^1^, Zhizhen Zhao ^1^, Sarah Formentini ^1^, Gregory Damhorst ^1,2,3^, Lucas Quinlan ^2^, James Kumar ^3^, Brad Weir ^3^, Karen White ^3^, Bobby Reddy Jr ^1,2^

#### ^1^University of Illinois at Urbana Champaign, Champaign, IL, USA; ^2^Prenosis Inc., Champaign, IL, USA; ^3^Carle Foundation Hospital, Urbana Champaign, Champaign, IL, USA

##### **Correspondence:** Ishan Taneja (itaneja2@illinois.edu)

Background

Sepsis is a syndrome defined as life-threatening organ dysfunction caused by a dysregulated host response triggered by an infection [1]. To date, numerous studies have captured aspects of the host response by measuring biomarkers pertinent to sepsis in human subjects [2,3]. Historically, the aim of these studies has been to demonstrate potential diagnostic or prognostic value of a set of biomarkers as opposed to specifically characterizing the degree of dysregulation of the host response. In our study, we sought to quantify the degree of dysregulation of the host response in a clinically relevant population in an intuitive and unbiased manner.

Materials and methods

In a cohort of 252 patients (Table 1) suspected of infection, we measured 15 biomarkers spanning various aspects of the host response as a function of time per patient. We specifically hypothesized that a multi-dimensional measure of time-dependent abnormality (termed the ‘personalized immune score’) could serve as a useful surrogate for quantifying dysregulation. We realize this may not capture the spirit of “dysregulation” but believe it could be a useful step forward toward that goal. We quantified multi-dimensional time-dependent abnormality through the formula$\sqrt{{\left(x-y\right)}^T{S}^{-1}\left(x-y\right)}$, where *x* referred to a set/subset of 15 biomarker measurements at a given time point for a given patient, *S* referred to the covariance matrix of all biomarker measurements, and *y* referred to a set/subset of 15 biomarker measurements at discharge for a given patient (Fig. 1). We tested the association of relevant features/variables with respect to the following sepsis-related outcomes: ICD9/10 codes (sepsis/severe sepsis/septic shock), the sepsis-3 definition, clinically significant bacteremia, and hospital length of stay.

Results

We constructed three variants of the personalized immune score: one using all the biomarkers, one using only ‘low signal’ biomarkers, and one using only ‘high signal’ biomarkers. We assessed the associations of each of the variants of the personalized immune score and the respective biomarkers that composed each of the immune scores with respect to our outcomes of interest (Fig. 2A-C). We also compared the optimized personalized immune score with a patient’s SIRS and SOFA score. (Fig. 2D). Raw p-values for each entry in the heatmap are reported in Tables 2, 3, 4 and 5, with the worst p-value (termed the ‘bottleneck p-value’) highlighted in red. In Fig. 3, we visualized the optimized personalized immune score as a function of time stratified by outcome.

Overall, there was a greater number of stronger associations between each outcome and all variants of the personalized immune score compared to that of any individual biomarker or EMR feature. Furthermore, the optimized immune score yielded stronger associations than any individual biomarker for three out of the four sepsis-related outcomes. Finally, when examining the bottleneck p-value in each table, all variants of the personalized immune score achieved the lowest value.

Conclusion

This study shows that a multi-dimensional measure of time-dependent abnormality of biomarkers that span various aspects of the host response could be a useful metric in evaluating the health state of a septic patient. Ultimately, we believe our work lays a foundation for quantifying the dysregulation of the host response in an unbiased manner.

References

1. Singer M, Deutschman CS, Seymour CW, Shankar-Hari M, Annane D, Bauer M, et al. The Third International Consensus Definitions for Sepsis and Septic Shock (Sepsis-3). JAMA. 2016;315:801.

2. Pierrakos C, Vincent J-L. Sepsis biomarkers: a review. Crit Care [Internet]. BioMed Central; 2010 [cited 2018 Feb 8];14:R15. Available from: http://ccforum.biomedcentral.com/articles/10.1186/cc8872

3. Biron BM, Ayala A, Lomas-Neira JL. Biomarkers for sepsis: What is and what might be? Biomark Insights. 2015;10:7–17.

**Table 1 (abstract P42).** See text for description

Characteristic		All Patients (N = 252)
Age, SD		56, 18
Gender		43% male, 57% female
Comorbid Conditions	CKD	16%
	COPD	18%
	Diabetes	30%
	Cancer	26%
Positive Blood Culture		14%
SOFA score, SD		2.65, 2.59
SIRS score, SD		1.89, .94
Sepsis-3		42%
ICD9/10 code for sepsis/severe sepsis/septic shock		33%

**Table 2 (abstract P42).** See text for description

	IL-6	nCD64	Pentraxin3	PCT	Immune Score (Optimal)
Sepsis	**0.027289**	0.001975	0.002794	0.00149	**0.000585**
Sepsis-3	0.000248	**0.010245**	**0.004557**	**0.013857**	1.43E-05
Bacteremia	4.24E-05	3.28E-05	8.72E-05	0.000136	5.17E-07
Length of Stay	3.58E-09	4.42E-10	6.10E-10	1.44E-15	1.73E-11

**Table 3 (abstract P42).** See text for description

	IL-6	MIP-1a	G-CSF	MCP-1	IP-10	IL-1ra
Sepsis	**0.028914**	0.292497	**0.105219**	0.036001	0.079042	0.066835
Sepsis-3	0.000267	**0.915809**	0.070411	**0.080203**	0.037564	0.438506
Bacteremia	4.50E-05	0.082711	0.000267	1.99E-08	**0.101414**	**0.646896**
Length of Stay	3.81E-09	0.000714	4.68E-05	2.02E-05	2.84E-09	0.000179
	nCD64	TREM-1	RAGE	TRAIL	C5a	Protein-C
Sepsis	0.002049	0.023998	**0.260936**	0.246075	**0.822951**	0.284126
Sepsis-3	**0.010829**	0.039883	0.157752	0.302052	0.103703	0.401148
Bacteremia	3.43E-05	**0.167446**	0.199506	**0.338874**	0.291458	**0.496485**
Length of Stay	4.89E-10	1.44E-05	1.80E-07	2.50E-05	7.90E-13	0.000712
	Pentraxin3	PCT	MMP-9	Immune Score		
Sepsis	0.002939	0.001571	**0.822951**	**0.004303**		
Sepsis-3	**0.004842**	**0.014454**	0.620514	0.000152		
Bacteremia	9.54E-05	0.00015	0.70316	1.10E-05		
Length of Stay	6.26E-10	1.29E-15	0.019903	1.23E-11		

**Table 4 (abstract P42).** See text for description

	MIP-1a	G-CSF	MCP-1	IP-10	IL-1ra	TREM-1
Sepsis	0.292497	**0.105219**	0.036001	**0.079042**	0.066835	0.023998
Sepsis-3	**0.915809**	0.070411	**0.080203**	0.037564	0.438506	0.039883
Bacteremia	0.082711	0.000267	1.99E-08	0.101414	**0.646896**	**0.167446**
Length of Stay	0.000714	4.68E-05	2.02E-05	2.84E-09	0.000179	1.44E-05
	RAGE	TRAIL	C5a	Protein C	MMP-9	Immune Score (Non-Optimal)
Sepsis	**0.260936**	0.246075	**0.822951**	0.284126	**0.822951**	**0.007219**
Sepsis-3	0.157752	0.302052	0.103703	0.401148	0.620514	0.00702
Bacteremia	0.199506	**0.338874**	0.291458	**0.496485**	0.70316	3.89E-05
Length of Stay	1.80E-07	2.50E-05	7.90E-13	0.000712	0.019903	2.99E-11

**Table 5 (abstract P42).** See text for description

	SIRS (Initial)	SIRS (Initial-Final)	SOFA (Initial)	SOFA (Initial-Final)	Immune Score (Optimal)
Sepsis	0.000121	0.000498	**0.0083**	**0.0141**	**0.000585**
Sepsis-3	**0.016297**	0.015438	2.38E-22	4.11E-07	1.43E-05
Bacteremia	0.119417	**0.023998**	0.00342	0.013683	5.17E-07
Length of Stay	0.006232	4.72E-07	0.000764	3.47E-10	1.73E-11

**Fig. 1 (abstract P42).**
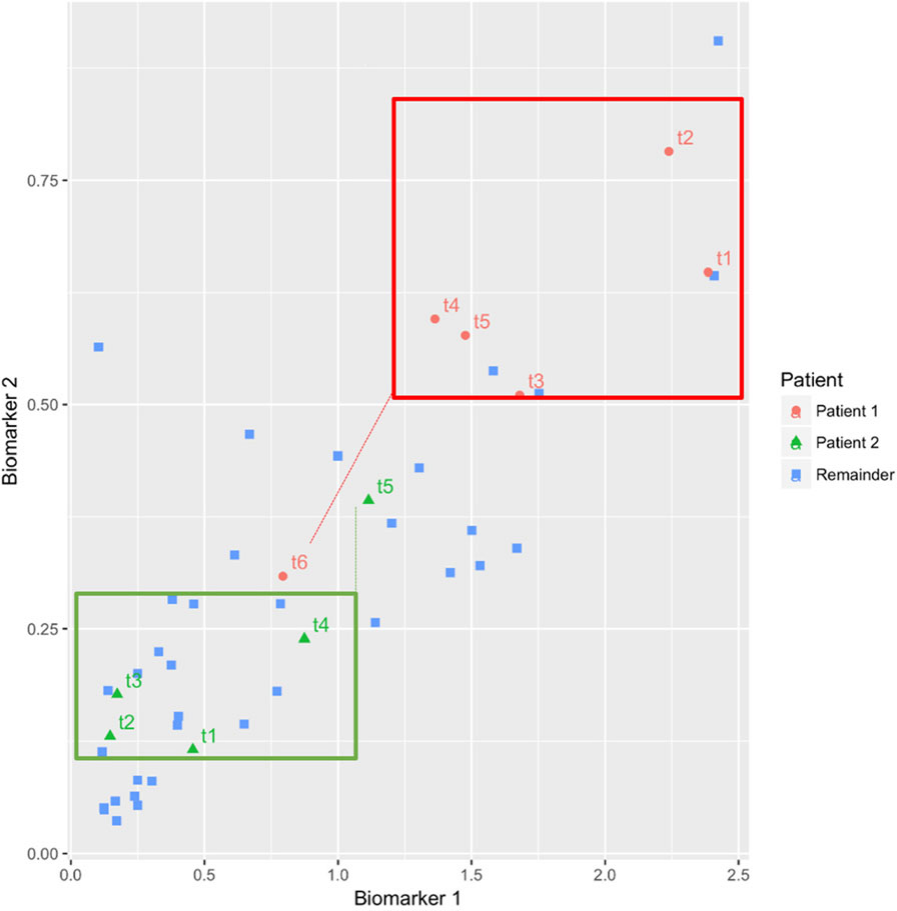
See text for description

**Fig. 2 (abstract P42).**
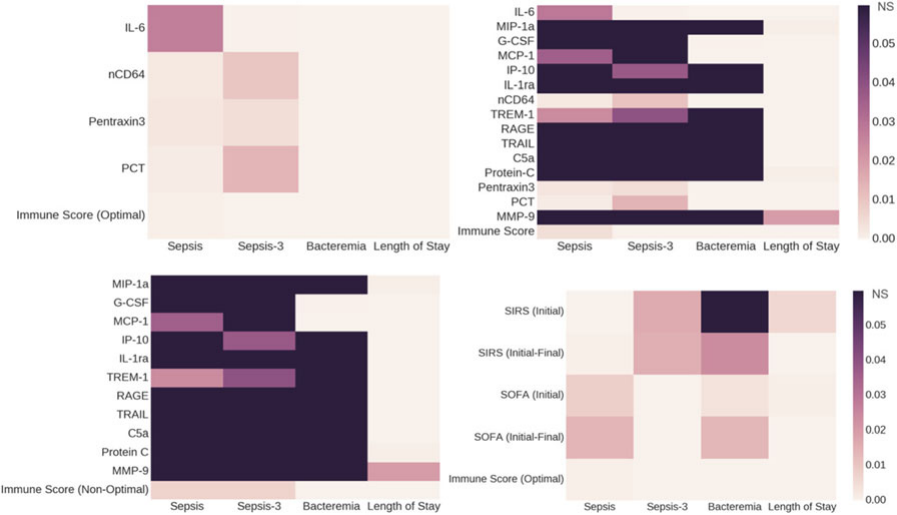
A) top left, B) top right, C) bottom left, D) bottom right

**Fig. 3 (abstract P42).**
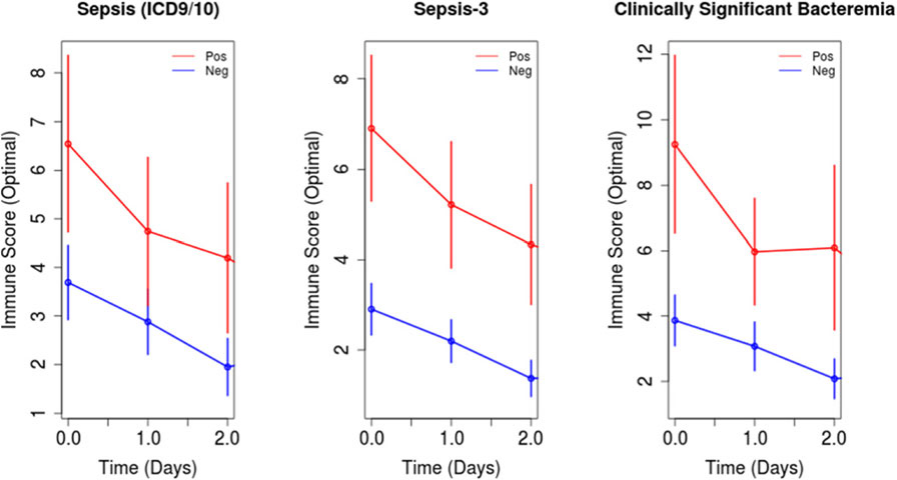
See text for description

## P43 Withdrawn

## P44 Independent validation of a novel 12-mRNA score for sepsis prognosis

### Timothy E Sweeney^1^, Melissa Remmel^1^, David Rawling^1^, Mark Eshoo^1^, Oliver Liesenfeld^1^, Charalambos Gogos^2^, Evangelos Giamarellos-Bourboulis^3^

#### ^1^Inflammatix, Inc., Burlingame, CA, USA; ^2^Department of Internal Medicine, University of Patras, Patras, Greece; ^3^4th Department of Internal Medicine, National and Kapodistrian University of Athens, Athens, Greece

##### **Correspondence:** Timothy Sweeney (tim@inflammatix.com)

Background

Improved prognostic and risk-stratification tools for patients with sepsis are needed both for improved clinical resource allocation and for prognostic enrichment in clinical trials [1]. We recently described a 12-mRNA host-response signature that, when measured quantitatively in whole blood, can accurately predict 28-day mortality [2]. In particular, we previously showed that the 12-gene ‘Stanford’ score has prognostic power both alone (AUROC mean 0.89, range 0.68-1.0 across 9 validation cohorts) and in combination with standard risk-stratification tools (such as SOFA) when linearly combined (mean boost in AUROC 0.10).

Materials and methods

Patient with sepsis according to Sepsis-2 criteria were enrolled at admission across multiple Greek ICUs. Whole blood was drawn into PAXgene RNA tubes at admission and frozen. For a pilot subset of 29 patients, RNA extraction (Qiagen QIAcube) was followed by measuring the 12-mRNAs using NanoString nCounter with a custom codeset. We combined expression levels of the 12 mRNAs into a single score as previously described [2]. Scores were used to directly calculate AUROCs, and also combined with clinical risk scores using linear regression trained locally.

Results

The 29 patients had a mean age of 68 +/- 13 years. By Sepsis-3 criteria, 9 had suspected infection but not sepsis; 11 had sepsis; 9 had septic shock. There were 20 survivors and 9 deaths. The AUROCs for predicting 28-day mortality were: qSOFA, 0.68 (95%CI 0.57-0.78); SOFA, 0.92 (95%CI 0.86-0.99); 12-mRNA score, 0.82 (95%CI 0.73-0.91). The AUROCs for linear combinations of qSOFA+12-mRNA score and SOFA+12-mRNA score were 0.86 (95%CI 0.78-0.94) and 0.96 (95%CI 0.91-1.0). 12-mRNA scores thus boosted AUROCs by 0.18 and 0.04, respectively, versus qSOFA and SOFA alone.

Conclusions

The 12-mRNA host-response signature continues to show validity for the prognosis of 28-day mortality in patients with sepsis in independent validation. Its ability to improve on standard prognostic scores such as qSOFA and SOFA is reconfirmed. A larger set of samples from the described cohort will be analyzed to increase the statistical power and overall validity of the results.

References

1. Opal SM, Dellinger RP, Vincent JL, Masur H, Angus DC. The next generation of sepsis clinical trial designs: what is next after the demise of recombinant human activated protein C?*. Crit Care Med. 2014;42(7):1714-21. doi: 10.1097/CCM.0000000000000325. PubMed PMID: 24717456.

2. Sweeney TE, Perumal TM, Henao R, Nichols M, Howrylak JA, Choi AM, et al. A community approach to mortality prediction in sepsis via gene expression analysis. Nat Commun. 2018;9(1):694. Epub 2018/02/15. doi: 10.1038/s41467-018-03078-2. PubMed PMID: 29449546; PubMed Central PMCID: PMCPMC5814463.

## P45 Cost effectiveness of emergency department use of a novel multi-mRNA assay for diagnosis and risk assessment of acute respiratory tract infections and sepsis

### Ivana Stojanovic^1^, Jonathan Romanowsky^2^, Philippe Schuetz^3,4,^ John Schneider^1^, Oliver Liesenfeld^2^, Timothy E Sweeney^2^

#### ^1^Avalon Health Economics, LLC, Morristown, New Jersey, 07960, USA; ^2^Inflammatix Inc., Burlingame, California 94010, USA; ^3^Division of General and Emergency Medicine, University Department of Medicine, Kantonsspital Aarau, Tellstrasse, 5001 Aarau, Switzerland; ^4^Medical Faculty of the University of Basel, Basel, Switzerland

##### **Correspondence:** Timothy Sweeney (tim@inflammatix.com)

Background

Emergency physicians currently rely on multiple imperfect tests to diagnose acute respiratory tract infections (ARTIs) and sepsis, leading to excess morbidity and mortality. A novel 30-host-gene PCR test (HostDx™ Sepsis by Inflammatix) identifies i) the likelihood of a bacterial infection, ii) the likelihood of a viral infection, and iii) the likelihood of sepsis and disease progression, in <60 minutes. This work examines the cost effectiveness of the HostDx Sepsis test for use in US healthcare system emergency departments (ED) for the assessment of ARTIs and sepsis.

Materials and methods

A decision-analytic simulation model compares the standard of care for patients assessed in the ED for ARTIs and suspected sepsis to a hypothetical care pathway with the HostDx Sepsis test. Analysis informed on expected costs and outcomes occurring during an ED visit for a hypothetical cohort of patients. Selected input outcomes were based on review of peer-review articles. The 30-day outcomes considered in the study were expected cost per person presenting with ARTI or suspected sepsis to an ED, incremental cost per life-year saved, antibiotics days and hospital length of stay (HLOS).

Results

In the base-case scenario, across a cohort of 1,000, the HostDx Sepsis arm saves 10 lives/1,000 person-years and results in expected costs of $1,404/person less than the standard of care arm, reduced total antibiotics exposure by 1,097 days and 341 days of hospital stays comparing to the standard of care arm. Standard of care relied on empirical treatment, clinical judgment and multiple testing for bacterial and viral infections, with accuracy for bacterial, viral and mortality risk differentiation lower than that of HostDx Sepsis. In one- and two-way sensitivity analysis, model results were most sensitive to the HLOS, followed by the HostDx Sepsis test price, and length of antibiotics treatment.

Conclusions

The HostDx Sepsis arm demonstrated clinical utility and cost effectiveness versus the current standard of care arm. Improved care is reflected by fewer unnecessary antibiotic prescriptions and side effects, fewer admissions and shorter HLOS. Interventional studies are necessary to compare actual clinical and costs outcomes between current practice and that which incorporates HostDx Sepsis.

## P46 Value of creatinine, prothrombin time and procalcitonin for predicting sepsis in patients with severe trauma

### Xiao-yuan Ma, Huai-jian Jin, Qi Huang, Wei Ma, Hua-ping Liang

#### State Key Laboratory of Trauma, Burns and Combined Injury, Research Institute of Surgery, The Army Medical University, Chongqing 400042, People’s Republic of China

##### **Correspondence:** Xiao-Yuan MA (M13658372627@163.com)

Background

The main biomarkers for diagnosis and prognosis of sepsis are the following three categories: acute phase proteins, vascular related biomarkers, cytokines. However, it is still controversial whether these large numbers of biomarkers improves prediction of sepsis in trauma population. The aim of this study was to explore the predictive efficiency of new model based on creatinine, prothrombin time and procalcitonin in severe trauma patients.

Materials and methods

This was a retrospective observational analysis (reviewing the medical records) of 593 patients with severe trauma hospitalized in ICU of the Daping Hospital of Army Medical University from January 1, 2012 to December 31, 2017. Two models were established: Model-1, a logistic regression model with sepsis as the response value and mechanical ventilation, shock and APACHE-II at ICU admission as explanatory variables; and Model-2, the same variables as in Model-1, with creatinine, prothrombin time and procalcitonin added as explanatory variables. Data were processed with t test, chi-square test, and receiver operating characteristic (ROC) curves were determined for both two models. The discrimination indexes (C-index and Nagelkerke's R2) and calibration curves for predicted sepsis versus observed sepsis were calculated for each model.

Results

There were 18527 trauma patients admitted at Daping Hospital of Army Medical University from January 1, 2012 to December 31, 2017. The study population of 593 cases with severe trauma hospitalized in ICU were ≥16 years old, incoming ICU within 24 hours after injury, the length of ICU stay ≥48 hours, ISS≥16, perfect clinical data, and without coexisting illness. 252 cases (42.50%) of 593 patients examined sepsis in hospital. In Model-2, creatinine, prothrombin time and procalcitonin were each a significant independent risk factor for sepsis diagnosis. The LR test comparing between Model-1 and Model-2 indicated significant improvement in Model-2 (P<0.001). Model-2 resulted in better values than Model-1 for discrimination indexes (C-index: 0.989 VS 0.941, Nagelkerke's R2: 0.694 VS 0.570). ROC curves for Model-1 and Model-2 were 0.793 and 0.879 (P<0.001). Calibration improved the model fit: the mean absolute errors were 0.027 and 0.005 and the mean squared errors were 0.00069 and 0.00020 for Model-1 and Model-2, respectively.

Conclusions

In conclusion, the ability of a new model to predict sepsis in severe trauma patients improved following the addition of creatinine, prothrombin time and procalcitonin measured on ICU arrival within 24 hours as explanatory variables.

Acknowledgements

The author thanks ICU of Chongqing Daping Hospital for providing valuable information.

Sources of Funding This study was supported by the National Nature Science Foundation of China (NSFC, No. 81671906).

## P47 Simplified sepsis-3 criteria using modified SOFA (MSOFA) score: A proposed sepsis diagnosis criteria in low-middle income country

### Robert Sinto^1^, Suhendro Suwarto^1^, Khie Chen Lie^1^, Kuntjoro Harimurti^2^, William Djauhari^3^, Djoko Widodo^1^, Herdiman T. Pohan^1^

#### ^1^Division of Tropical and Infectious Diseases, Department of Internal Medicine, Faculty of Medicine Universitas Indonesia, Cipto Mangunkusumo National Hospital, Jakarta, Indonesia; ^2^Center for Clinical Epidemiology and Evidence Based Medicine, Faculty of Medicine Universitas Indonesia, Cipto Mangunkusumo National Hospital, Jakarta, Indonesia; ^3^Faculty of Medicine, Atma Jaya Catholic University of Indonesia, Jakarta, Indonesia

##### **Correspondence:** Robert Sinto (rsinto@yahoo.com)

Background

The complexity of sepsis-3 criteria based on Sequential Organ Failure Assessment (SOFA) score is underlined by the need to perform routine laboratory tests for fulfillment of the diagnosis criteria [1,2]. Modified SOFA (MSOFA) has been previously proposed as an alternative of SOFA score in predicting mortality of critical ill patients [3]. This study investigated the potential role of MSOFA score as a substitute of SOFA score in sepsis diagnosis criteria in hospital of low-middle income country, in comparison with quick SOFA (qSOFA) criteria.

Materials and methods

Prospective cohort study was conducted consecutively between April and November 2017 in suspected infection adult (aged 18 years and older) patients visiting Emergency Room of Cipto Mangunkusumo National Hospital, Indonesia. All variables from SOFA, MSOFA, qSOFA were collected. Patients were followed up until hospital discharge or death. Area under the receiver operating characteristic curve (AUROC) of modified criteria using increment of MSOFA, sepsis-3 diagnosis criteria (i.e. increment of two or more SOFA score consequent to the infection [1]) and qSOFA criteria were evaluated.

Results

Of 2695 patients screened, 1078 met the inclusion criteria and were included in the analysis. Mean age was 50 years (standard deviation 15.01 years). The two most common comorbidities were malignancy (29.5%) and diabetes mellitus (23.3%). Pneumonia (66.4%) and skin, soft tissue infection (20.4%) were two most common sources of infection. In-hospital mortality was 35.2%. The modified criteria using increment of MSOFA score showed good performance in predicting in-hospital mortality with an AUROC of 0.733 (95% CI 0.702-0.764). Sepsis-3 diagnosis criteria showed an AUROC of 0.752 (95% CI 0.723-0.782), while the AUROC of qSOFA criteria was 0.696 (95% CI 0.662-0.73) (Fig. 1). Using cut off >=2, modified criteria using increment of MSOFA showed sensitivity 74.7% and specificity 57.4%, while the sepsis-3 criteria showed sensitivity 75.5% and specificity 63.7%.

Conclusions

Simplified sepsis-3 criteria using increment of MSOFA score as a substitute of SOFA score shows a good performance and could potentially be applied in hospital of low-middle income country.

Acknowledgements

This research was funded by Cipto Mangunkusumo National Hospital Operational Research Grant 2017.

References

1. Singer M, et al: The third international consensus definitions for sepsis and septic shock (sepsis-3). JAMA 2016, 315:801-810.

2. Seymour CW, et al: Assessment of clinical criteria for sepsis: for the third international consensus definitions for sepsis and septic shock (sepsis-3). JAMA 2016, 315:762-774.

3. Grissom CK, Brown SM, Kuttler KG, Boltax JP, Jones J, Jephson AR, Orme Jr JF: A modified sequential organ failure assessment score for critical care triage. Disaster Med Public Health Prep 2010, 4:277-284.

**Fig. 1 (abstract P47).**
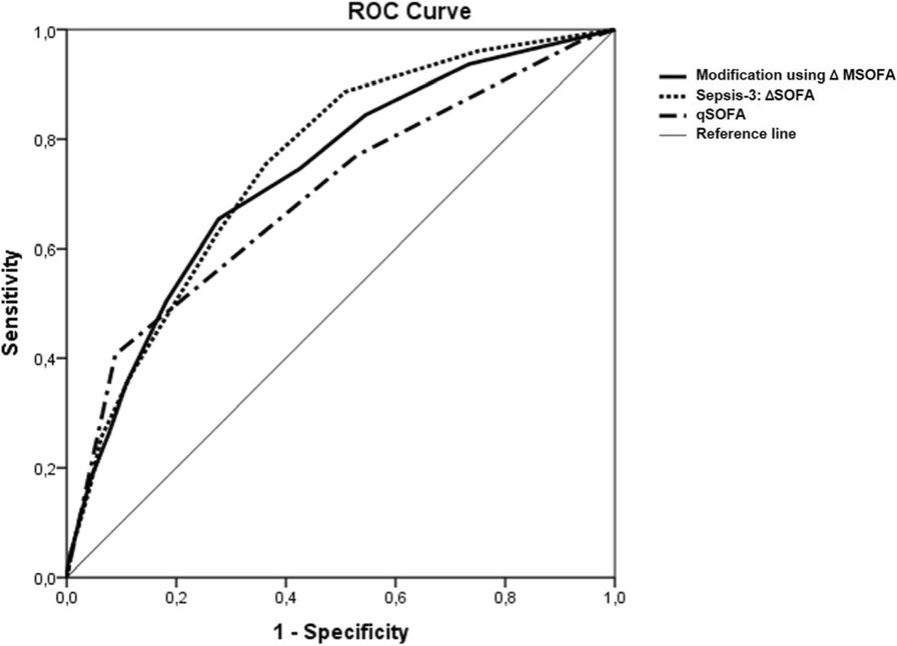
See text for description

## P48 Accuracy of qSOFA score to predict sepsis-related mortality in 99 studies consisting of 588,883 patients: a systematic review and meta-analysis

### Velma Herwanto^1,2^, Amith Shetty^3^, Guy Eslick^4^, Benjamin Tang^1,2,4^

#### ^1^Department of Intensive Care Medicine, Nepean Hospital, Sydney, NSW, Australia; ^2^Centre for Immunology and Allergy Research, Westmead Institute for Medical Research, University of Sydney, Sydney, NSW, Australia; ^3^Department of Emergency Medicine, Westmead Hospital, Sydney, NSW, Australia; ^4^Nepean Clinical School, University of Sydney, Sydney, NSW, Australia

##### **Correspondence:** Velma Herwanto (vher6289@uni.sydney.edu.au)

Background

Early recognition of sepsis can reduce mortality. Sequential Organ Failure score (qSOFA) is recently introduced to facilitate early sepsis detection. Here, we performed a meta-analysis to assess whether qSOFA is better than systematic inflammatory response syndrome (SIRS) in identifying patients at risk of dying from sepsis.

Materials and methods

We searched (without any language restriction) multiple electronic databases including PubMed, the Cochrane Library, Embase, Web of Science and Google Scholar to identify observational studies (up to 15 June 2018) that assessed predictive performance of qSOFA and SIRS, two of the most widely used outcome prediction scores in sepsis. Studies were included if the outcome measures were mortality, organ dysfunction or admission to intensive care unit (ICU) and if prediction performance were reported as either area under the curve (AUC), sensitivity or specificity. We analysed the predictive performance of both qSOFA and SIRS using a random effect model and we explored heterogeneity by performing pre-specified subgroup analyses.

Results

Ninety-nine studies consisting of 588,883 patients were included in the analysis (Table 1). As expected, qSOFA showed a lower sensitivity 52.8% (95% confidence interval 40.5-65.1%) and a higher specificity 74.6% (95% confidence interval 69.7-79.5%), compared to SIRS which showed a higher sensitivity 85.4% (95% confidence interval 80-90.8%) and a lower specificity 35.2% (95% confidence interval 6.3-64.1%). The overall prediction accuracy of qSOFA was higher than SIRS, as measured by area-under-the-curve (AUC) in all prediction models. For mortality prediction, qSOFA (AUC 0.694) was better than SIRS (AUC 0.598) (qSOFA vs. SIRS; p<0.01) (Fig. 1). Similarly, for prediction of composite outcomes (organ dysfunction or admission to ICU), qSOFA (AUC 0.694) was better than SIRS (AUC 0.637) (qSOFA vs. SIRS; p<0.01) (Fig. 2). The higher performance of qSOFA was consistent in all subgroup analyses; qSOFA outperformed SIRS (in predicting mortality, organ dysfunctions or ICU admission) irrespective of study design (prospective vs. retrospective), patients populations (ER vs. ICU), case mix (infection vs. any conditions) or countries (developed vs. resource-limited).

Conclusions

We have conducted the largest meta-analysis of qSOFA and SIRS to date. Our findings showed that these two scores have complimentary performance (qSOFA more specific vs. SIRS more sensitive). However, for overall prediction accuracy (mortality, organ dysfunction or ICU admission), qSOFA has a higher performance compared to SIRS and this difference is consistent throughout most studies and across a diverse range of clinical settings.

Acknowledgements

This research was partly supported by Indonesian Endowment Fund for Education (LPDP).

**Table 1 (abstract P48).** Characteristics of Included Studies

		Studies	Patients
Total		99	588,883
Study design	Prospective	29	84,947
	Retrospective	70	506,258
Population	ED	58	394,346
	Any (including ED and ICU)	20	172,466
	ICU	10	17,765
	Prehospital	5	2,641
	Non-ICU	5	1,445
	Outpatient	1	220
Case mix	Infection/sepsis	91	563,470
	Respiratory tract infection	7	25,272
	Urinary tract infection	1	141
Setting	Developed countries	89	572,643
	Resource-limited countries	10	16,240
Baseline condition/ comorbidity	Unspecified	90	584,898
	Elderly	3	2,043
	Malignancy	3	1,277
	Cirrhosis	1	259
	On hemodialysis	1	220
	Hematology cases	1	186
Outcome measures	Hospital mortality	63	361,019
	28/ 30 days mortality	35	221,001
	Early (48 hours – 7 day) mortality	3	6,164
	90 day – 6 months mortality	1	1,784
	Any mortality	1	37
	ICU admission	24	124,004
	Ventilator support	3	33,705
	Prolonged ICU stay	9	29,935
	Organ dysfunction	4	16,634
Publications type	Full text articles	69	435,553
	Conference abstracts	30	153,330
Prediction score	Both SIRS and qSOFA	59	383,290
	qSOFA only	39	196,869
	SIRS only	1	8,724
Inclusion criteria	Fever	1	519
	Infection	54	362,440
	Sepsis-2	20	168,165
	Sepsis-3	7	2,237
	Either sepsis-2 or 3	16	55,041
	Positive blood culture	1	481

**Fig. 1 (abstract P48).**
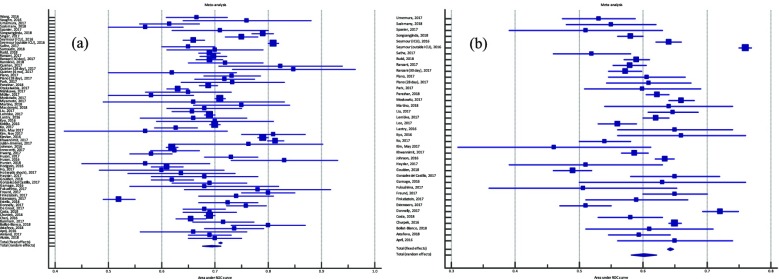
Forest plots of AUC for (a) qSOFA and (b) SIRS in predicting mortality

**Fig. 2 (abstract P48).**
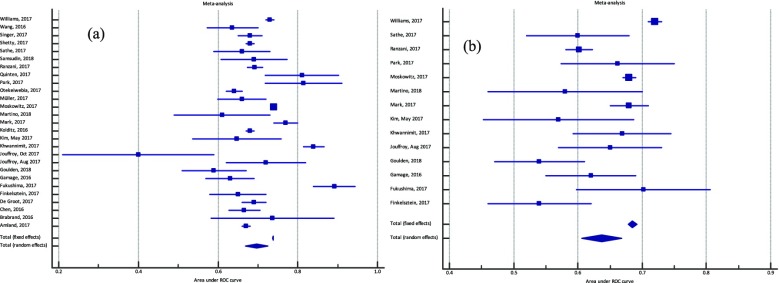
Forest plots of AUC for (a) qSOFA and (b) SIRS in predicting composite outcomes (organ dysfunction or admission to ICU)

## P49 Metabolic profile of peripheral blood mononuclear cells in patients who are at risk of developing sepsis

### Velma Herwanto^1^, Ya Wang^1^, Maryam Shojaei^1^, Kevin Lai^2^, Amith Shetty^2^, Benjamin Tang^1,3^, Anthony McLean^3^, David Booth^1^

#### ^1^Centre for Immunology and Allergy Research, Westmead Institute for Medical Research, Westmead, NSW, Australia; ^2^Department of Emergency Medicine, Westmead Hospital, Westmead, NSW, Australia; ^3^Department of Intensive Care Medicine, Nepean Hospital, Kingswood, NSW, Australia

##### **Correspondence:** Velma Herwanto (vher6289@uni.sydney.edu.au)

Background

Sepsis (infection complicated by organ dysfunction) is associated with a dysregulated immune response. The mechanism of immune dysregulation is not well understood but impaired energy metabolism in circulating leukocytes is thought to play a role [1,2]. Here, to gain mechanistic insight into immune dysregulation, we measured leukocyte metabolism and apoptosis in infected patients who were at risk of developing sepsis.

Materials and methods

Peripheral blood mononuclear cells (PBMC) were isolated from whole blood of healthy controls (n=8), low-risk (n = 6) and high-risk infection patients (n = 10). Low risk infection was defined as clinical suspicion of infection with a normal qSOFA score (qSOFA is a prediction index for the subsequent development of sepsis). High risk infection was defined as clinical suspicion of infection plus an increase of 2 points in qSOFA score. The PBMC metabolic profiles, total cellular reactive oxygen species (ROS) and the number of apoptosis cells were measured by Agilent Seahorse XF analyser (Agilent Technologies), DCFDA Cellular ROS Detection Assay (abcam) and Annexin V-FITC Apoptosis Detection kit (abcam), respectively. Total cellular ROS and apoptosis detection were further quantified by flow cytometry.

Results

The mitochondrial respiration in PBMC was significantly reduced in high risk infection patients as compared to healthy controls, especially in reserve capacity (maximal respiration and spare respiratory capacity). There was also a trend towards reduced mitochondrial respiration in low risk infection patients as compared to healthy controls (Fig. 1).

Conclusions

The metabolic switch to glycolysis, a normal compensatory response to mitochondrial suppression in infection, was not observed in the circulating leukocytes of patients with a higher risk for sepsis. This metabolic derangement was not related to the oxidative stress in the leukocytes. This novel finding reveals an unexpected level of complexity in the metabolic dysregulation of circulating leukocytes, thereby opening up a potential new avenue for further investigation.

References

1. Cheng SC, Scicluna BP, Arts RJW, Gresnigt MS, Lachmandas E, Giamarellos-Bourboulis EM, et al. Broad defects in the energy metabolism of leukocytes underlie immunoparalysis in sepsis. Nat Immunol 2016;17(4):406-13.

2. Park DW, Zmijewski JW. Mitochondrial dysfunction and immune cells metabolism in sepsis. Infect Chemother 2017;49(1):10-21.

**Fig. 1 (abstract P49).**
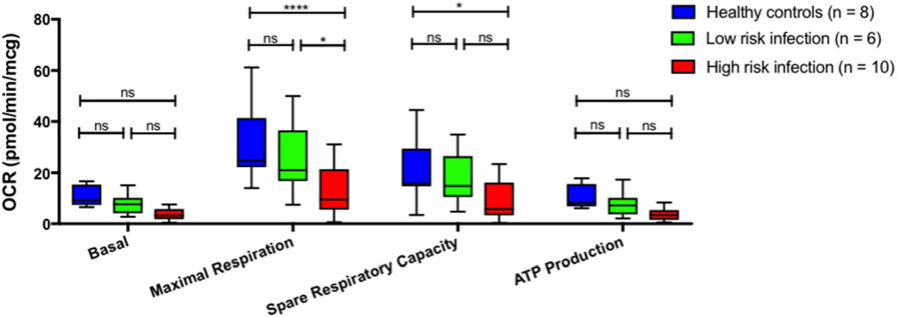
Seahorse assay parameters in healthy controls, low and high risk infection groups, measured in oxygen consumption rate (OCR). OCR represents cellular metabolism through mitochondrial respiration. Basal denotes baseline energetic demand of the cell; maximal respiration shows the maximum rate of respiration the cell can achieve; spare respiratory capacity denotes the capability of the cell to respond to energetic demand; ATP production shows ATP produced by the mitochondria to meet the cellular energetic need. p value (2-way ANOVA): ns >0.05, *0.01-0.05, ****<0.0001. Notably, this reduced mitochondrial respiration was not accompanied by a compensatory increase in glycolysis (Fig. 2)

**Fig. 2 (abstract P49).**
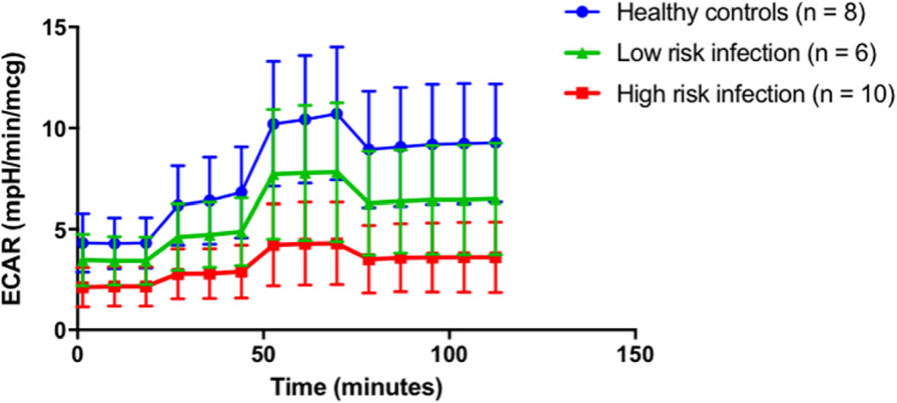
Extracellular acidification rate (ECAR)-time plot as measured by Seahorse assay in healthy controls, low and high risk infection group. ECAR represents cellular metabolism through glycolysis. Seeing no difference in total ROS production across the three groups (Fig. 3), indicating that oxidative stress did not play a role in reducing mitochondrial respiration in PBMC of high risk infection groups.

**Fig. 3 (abstract P49).**
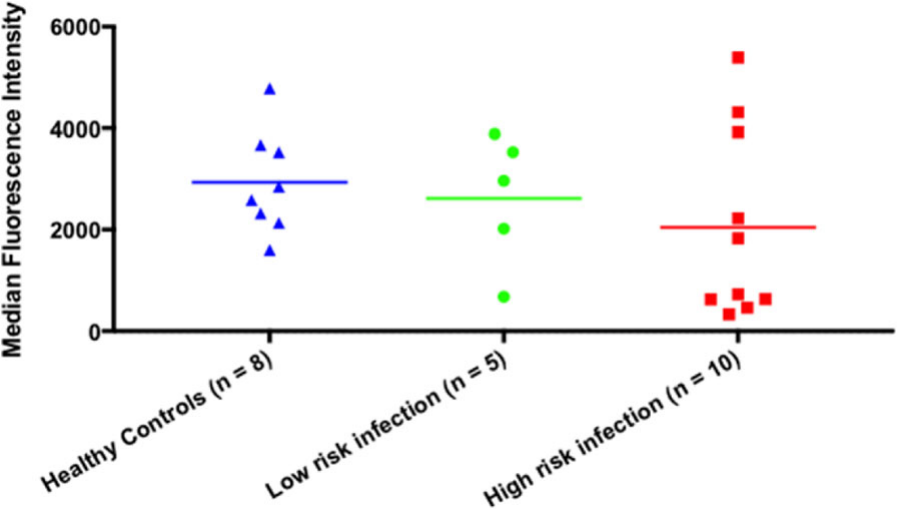
Total cellular ROS as measured by DCFDA with flow cytometry analysis (p = 0.4038, Kruskal-Wallis test). Fewer number of samples in low risk infection group was due to inadequate cell number. Apoptosis, a common sequela of reduced mitochondrial function, did not differ between groups; however, the high-risk group did show some signs of early apoptosis (Fig. 4).

**Fig. 4 (abstract P49).**
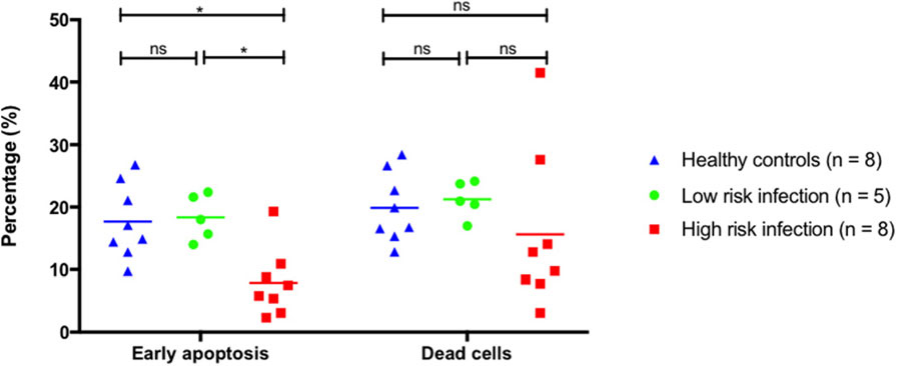
Number of apoptotic and dead cells as measured by Annexin V and Propidium Iodide (Pl) with flow cytometry analysis. Dead cells include the total of early apoptotic (Annexin V+ Pl-) and necrotic cells (Annexin V+ Pl+). p value (2-way ANOVA): ns >0.05, *0.01-0.05. Fewer number of samples in low and high risk infection groups were due to inadequate cell number

## P50 The development of an online scenario-based module to improve the identification and treatment of sepsis in a large NHS Trust

### Emma Mewse^1^, Katie McFaul^1^, Bhaveet Radia^2^

#### ^1^Infection Department, Guy’s and St Thomas’ NHS Foundation Trust, London, United Kingdom; ^2^Education, Training and Development Department, Guy’s and St Thomas’ NHS Foundation Trust, London, United Kingdom

##### **Correspondence:** Emma Mewse (Emma.Mewse@gstt.nhs.uk)

Background

Sepsis causes morbidity and mortality. In the UK, sepsis causes 250 000 cases and 44 000 deaths annually [1]. Better diagnosis and treatment could save 11,000 lives and £160 million each year [2]. Sepsis 6 are interventions [1,3-4] which can reduce mortality by 50% if all components are implemented within 60 minutes [5]. Local audit of patients with suspected and confirmed sepsis reveals low compliance with these interventions. We sought to develop a teaching strategy to improve sepsis recognition and management. Traditional classroom-based teaching (TCBT) methods and an online module were developed and staff sepsis training evaluated. These teaching methods included a case-study approach encompassing all facets of sepsis recognition and management. We sought to evaluate the acceptability and effectiveness of each intervention.

Materials and methods

Current local, national and international guidelines informed development and subject matter of both TCBT and an online module. This module is available to all hospital employees via local intranet and mobile devices (Fig. 1). Both teaching methods were departmentally peer-reviewed and trialled in pilot clinical areas.

Defined outcomes were specified:

• Acceptability and effectiveness of TCBT and online teaching

• Definition of sepsis, septic shock, sepsis red flags, Sepsis 6 components, desired time to management of sepsis

• Individuals’ confidence in sepsis management in a post-teaching survey

• Ongoing collection of qualitative feedback

Trust clinical staff members will be allocated either face-to-face training and/or an online module and asked to evaluate their experience and recall of key components

Results

1. TCBT- pre and post teaching results were recorded. There was a marked improvement in all areas specified including sepsis knowledge and confidence in sepsis management (Fig. 2).

2. Online module was well received by staff and was considered an effective and enjoyable way to learn. Data collection is ongoing, including the specified outcomes.

Conclusions

We have demonstrated benefits to staff sepsis knowledge from TCBT. Advantages of online teaching include availability, minimal disruption to clinical care, a standardised approach and recordable outcomes. Online learning modules and TCBT can improve sepsis care and management. High-quality online modules can deliver sepsis training and ensure all staff have access to learning resources. Our data provides insights into the learning experience. Future work will compare TCBT and online teaching.

Acknowledgements

We thank the staff at Guy’s and St Thomas’ hospital for their participation.

References

1. Daniels R and Nutbeam T eds. The Sepsis Manual 4th edition 2017-2018. https://sepsistrust.org/. Accessed 7th February 2018.

2. Department of Health and Social Care. New action to reduce sepsis. London, 2015. https://www.gov.uk/government/news/new-action-to-reduce-sepsis. Accessed 5th January 2018.

3. Sepsis: recognition, diagnosis and early management [NG51]: National Institute Clinical Excellence, London. 2016. https://www.nice.org.uk/guidance/ng51. Accessed 27th June 2018.

4. Levy M.M, Evans L.E and Rhodes A. The Surviving Sepsis Campaign Bundle: 2018 update. Intensive Care Med. 2018; https://doi.org/10.1007/s00134-018-5085-0. Accessed 21st June 2018.

5. Daniels R, Nutbeam T, McNamara G, and Galvin C. The sepsis six and the severe sepsis resuscitation bundle: a prospective observational cohort study. Emerg Med J 2011; 28:507e512. doi:10.1136/emj.2010.095067. Accessed 14th December 2017

**Fig. 1 (abstract P50).**
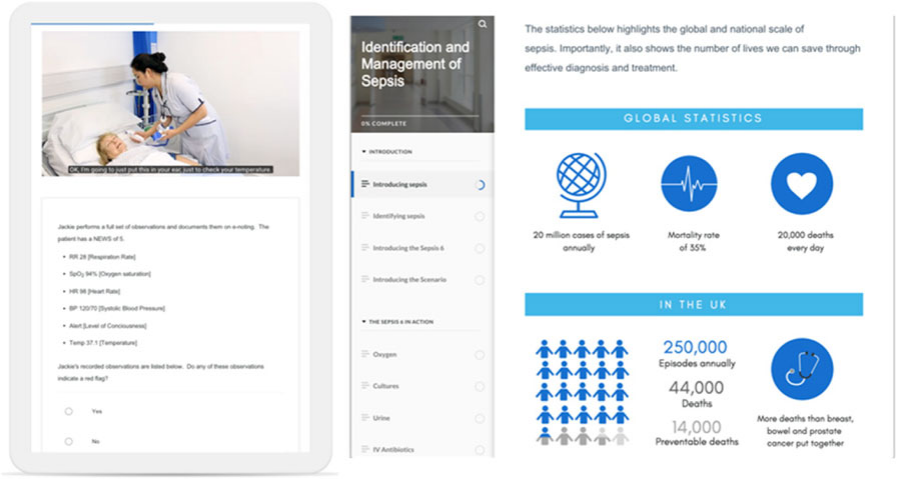
Screenshot of module on mobile device and desktop

**Fig. 2 (abstract P50).**
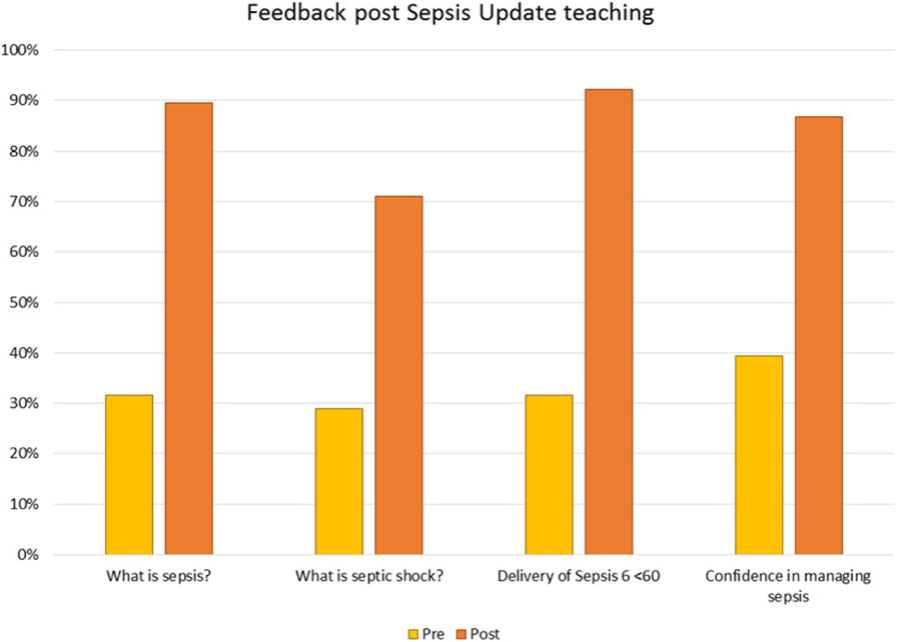
Questionnaire results pre/post teaching

## P51 Withdrawn

## P52 Withdrawn

